# Anticancer-active 3,4-diarylthiolated maleimides synthesis *via* three-component radical diarylthiolation

**DOI:** 10.3389/fchem.2022.1089860

**Published:** 2022-11-25

**Authors:** Limei Wang, Zhuo Li, Zhehan Ma, Kedi Xia, Wenyu Wang, Wenchang Yu

**Affiliations:** ^1^ Department of Clinical Pharmacy, Jilin Province FAW General Hospital, Changchun, China; ^2^ Department of Pharmacy, Affiliated Hospital of Changchun University of Chinese Medicine, Changchun, China; ^3^ Department of Otolaryngology, Jilin Province FAW General Hospital, Changchun, China; ^4^ Department of Pharmacy, Jilin Province FAW General Hospital, Changchun, China; ^5^ Department of Thoracic Surgery, Jilin Province FAW General Hospital, Changchun, China

**Keywords:** radical thiolation, copper-catalyzed, sulfur powder, maleimides, bisthiolation

## Abstract

Herein, we report an efficient and simple copper-catalyzed oxidative diarylthiolation of maleimides with sulfur powder and aryl boronic acids, in which S powder was used as a substrate and internal oxidant. The corresponding double C-S bonds coupling products were obtained in moderate to high yields under a simple catalytic system. Mechanistic studies indicated that copper-catalyzed radical thiolation of aryl boronic acids with S powder, and the resulting arylthiyl underwent radical addition with double bonds of maleimides.

## 1 Introduction

**SCHEME 1:**
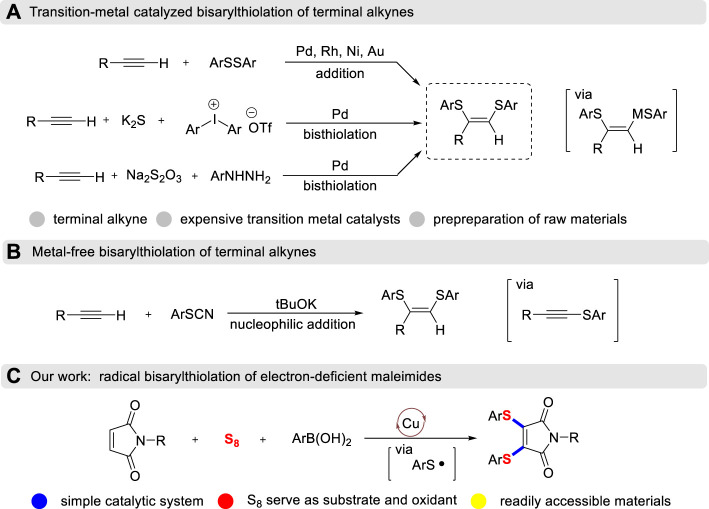
Strategies for diarylthiolated alkenes.

From frequently-used chelating ligands and promising bioactive compounds to wonderful organic electrode materials, vicinal diarylthiolated alkenes all play extremely prominent roles in different scientific fields (Gerken et al., 2020; Ryu et al., 2007). Transition-metal catalyzed cis-addition of disulfides to terminal alkynes is a reliable and efficient method for the construction of these prevalent skeletons with the formation of double C-S bonds (Ananikov et al., 2004; Ananikov et al., 2008; Wei et al., 2020; Arisawa et al., 2001; Cai et al., 2007; Kuniyasu et al., 1991; Sartori et al., 2013; Gao et al., 2022; Yang, 2019i) (Scheme 1A). Recently, a three-component cascade diarylthiolation using the methods of inputting S elemental was developed. Jiang has demonstrated palladium-catalyzed cascade diarylthiolation of terminal alkynes, K_2_S and diaryliodonium salts (Li et al., 2018). Arylhydrazines could be converted into functionalized diarylthiolated alkenes by palladium-catalyzed oxidative coupling method and Na_2_S_2_O_3_ reagent was employed as a sulfur source (Lai et al., 2021). Furthermore, a facile metal-free preparation of 1,2-diarylthiolated styrenes by the reaction of thiocyanate with terminal alkynes has also been developed (Scheme 1B). Although a handful of strategies have been developed toward molecular diversification, however, expensive transition-metal catalysts, pre-preparation of raw materials and stoichiometric amounts of H_2_O_2_ oxidant are required (Li et al., 2013). To the best of our knowledge, oxidative diarylthiolation of internal alkenes is still an important but unresolved challenge (Kodama et al., 2007), which is due to the steric hindrance environment between internal C=C bonds.

Maleimide is widely employed to conjugate with thiol groups through *S*-Michael addition reactions. The resulting thiolated maleimide has unique biological activities and is commonly used as a useful handle in organic synthesis (Morais et al., 2017). Recently, Zhao has developed copper-catalyzed sulfenylation of maleimides with thiols to give 3-thiomaleimides with the addition of fluoroboric acid (Yang et al., 2016). However, this transformation could not be utilized to access 3,4-diarylthiolated maleimides, even in the presence of excess thiols or a step-by-step strategy. The main intrinsic reason is that the introduction of the thio group would dramatically reduce the electrophilicity of maleimide, thus preventing the further *S*-Michael addition with another nucleophilic thiol. Inspired by the free radical cyclization of maleimides (Zhu et al., 2019), we envisioned the radical cascade diarylthiolation, that is, the *in-situ* generated arylthiyl radical intermediates which initiated from the copper-catalyzed thiolation of aryl boronic acids with sulfur powder. Then, the resulting arylthiyl went through radical nucleophilic addition/radical-radical cross-coupling/oxidative dehydrogenation steps with maleimides to achieve alkene diarylthiolation (Scheme 1C). This three-component radical diarylthiolation protocol not only provides a concise pathway to important 3,4-diarylthiolated maleimides (Palanki et al., 2013; Jones et al., 2012), but also explores the chemical reactivity of S powder.

## 2 Results and discussion

To test our assumptions, we set out to study the radical cascade reaction of N-phenyl maleimide with sulfur powder and phenylboronic acid (Table 1). After screening the common reaction parameters, when the reaction was stirred in the presence of 10% CuI in DMSO, at 100°C under air atmosphere, desired product **3a** was obtained with exclusive diarylthiolation and promising chemoselectivity. In addition, no byproducts of mono-arylthiolated or *S*-Michael addition byproducts were observed (entry 1). Other copper salts such as CuCl and Cu(OAc)_2_ gave relatively lower yields (entries 2, 3). Next, we tried to explore the effect of ligands, which have been well demonstrated in copper-catalyzed radical reaction systems (Su et al., 2015). But it is regrettable that the use of nitrogen-based ligands such as phen and trans-(1R, 2R) N,N′-dimethyl-cyclohexane-1,2-diamine, or bidentate phosphine ligands such as XantPhos and Binap, maleimide diarylthiolation was completely inhibited (entries 4-7). Fortunately, in the case of PCy_3_ as a ligand, the yield of the diarylthiolation product increased (entry 8). Therefore, we examined other monodentate phosphines. To our delight, XPhos could give promising reactivity, while DavePhos and SPhos show slightly lower performance (entries 9-11). The wise choice of solvent had a significant influence on the transformation. Only DMSO could give the desired product. In contrast, the reactions were completely shut down when other solvents were used (entries 12-14). Notably, lowering the reaction temperature to 90°C dramatically reduced the yield of **3a** (entry 15)**.** Finally, when the reaction was exposed to O_2_ atmosphere, the reaction efficiency was improved, and 77% yield of **3a** could be obtained (entry 16). It is worth noting that there’s no reaction at all under nitrogen atmosphere (entry 17).

**TABLE 1:** Reaction optimization^a^.

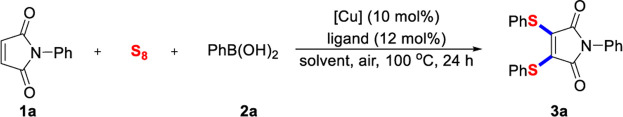				
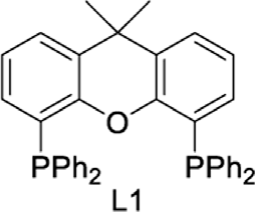	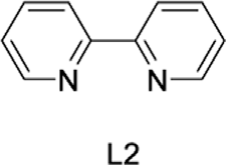	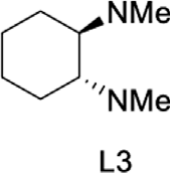	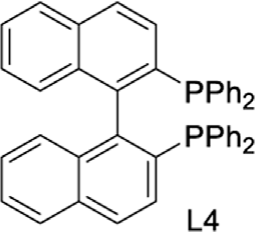
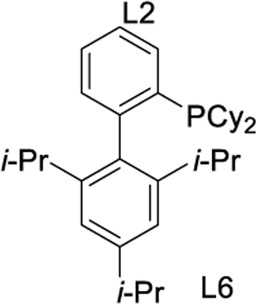	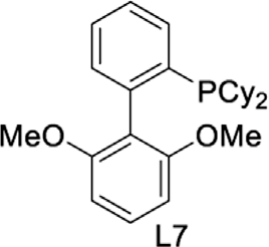	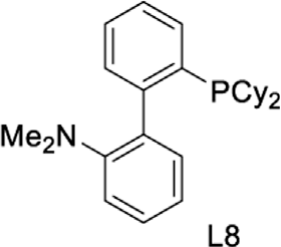		
Entry	Catalyst	Ligand	Solvent	Yield (%)^b^
1	CuI	—	DMSO	45
2	CuCl	—	DMSO	41
3	Cu(OAc)_2_	—	DMSO	6
4	CuI	L1	DMSO	trace
5	CuI	L2	DMSO	trace
6	CuI	L3	DMSO	trace
7	CuI	L4	DMSO	trace
8	CuI	L5	DMSO	60
9	CuI	L6	DMSO	72
10	CuI	L7	DMSO	45
11	CuI	L8	DMSO	49
12	CuI	L6	DMF	0
13	CuI	L6	toluene	0
14	CuI	L6	THF	0
15^c^	CuI	L6	DMSO	51
16^d^	CuI	L6	DMSO	77
17^e^	CuI	L6	DMSO	0

^a^ Reaction conditions unless specified otherwise: **1a** (0.2 mmol), sulfur powder (0.6 mmol), **2a** (0.6 mmol), copper catalyst (10 mol%) and ligand (12 mol%) in solvent (2.0 ml) were stirred at 100°C under air for 24 h.

^b^ Isolated yield.

^c^ At 90°C.

^d^ Under O_2_ atmosphere.

^e^ Under N_2_ atmosphere.

**SCHEME 2:**
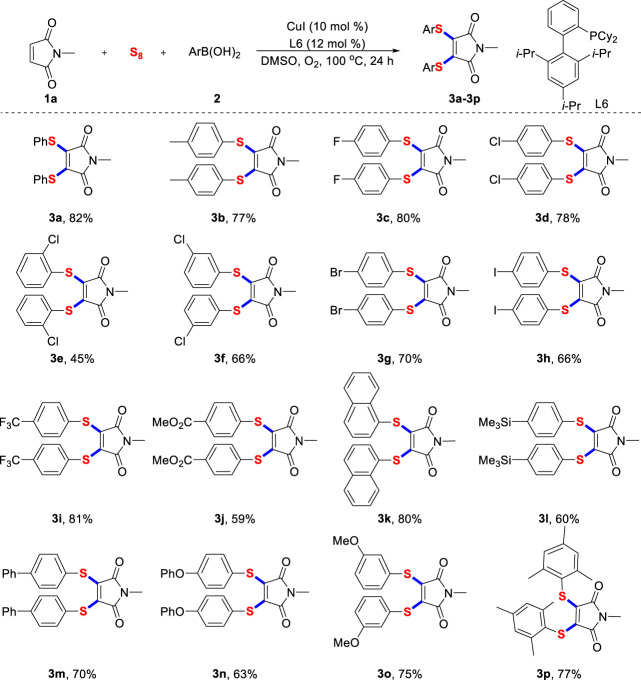
Scope of arylboronic acids^a^. ^a)^ Reaction conditions unless specified otherwise: **1a** (0.2 mmol), sulfur powder (0.6 mmol), **2** (0.6 mmol), CuI (10 mol%) and XPhos (12 mol%) in DMSO (2.0 ml) were stirred at 100°C under O_2_ atmosphere for 24 h, isolated yields are given.

Firstly, under the optimal reaction conditions, we synthesized various diarylthiolated maleimides with a variety of aryl boronic acids (Scheme 2). Generally, aryl boronic acids with different critical functional groups, whether electron-donating methyl (**3b**), phenoxyl (**3n**) and methoxyl (**3o**), or electron-deficient fluoro (**3c**), trifluoromethyl (**3i**) and ester (**3j**) were all competent substrates and facilely converted into the expected products. Surprisingly, the iodo and silicon functional groups (**3h**, **3L**) frequently applied in copper-catalyzed cross-coupling reactions are also compatible with our standard reaction conditions, which provide a great opportunity for further derivations of the target product. Interestingly, mesityl boronic acid (**3p**) with large steric hindrance also successfully participated in multi-component reactions and gave the corresponding product. This experimental phenomenon is consistent with the tendency of chloro atom at different positions of aryl boronic acid (**3d**-**3f**).

**SCHEME 3:**
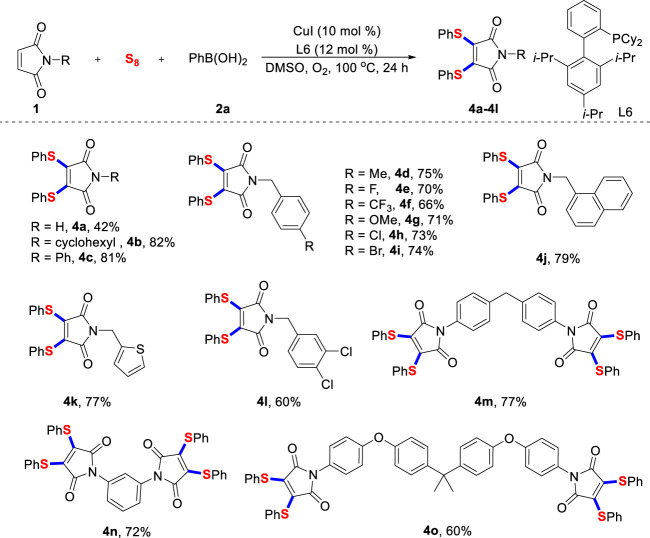
Scope of maleimides^a^. ^a)^ Reaction conditions unless specified otherwise: **1** (0.2 mmol), sulfur powder (0.6 mmol), **2a** (0.6 mmol), CuI (10 mol%) and XPhos (12 mol%) in DMSO (2.0 ml) were stirred at 100°C under O_2_ atmosphere for 24 h, isolated yields are given.

Then, significant efforts were dedicated to expand the range of maleimides with different structures (Scheme 3). On the whole, unprotected and *N*-benzylated maleimides effectively participated in this transformation and the desired products were accessed in good yields. Although the yield of **4a** was slightly lower, it confirmed that maleimide was tolerant under current reaction conditions. The established reaction conditions do not influence usually important functional groups, thus leaving an important space for further late-stage functionalization through mature cross-coupling reactions. It was found that thiophene-substituted maleimide was feasible, and the expected product **4k** could be isolated in good yield.

Unsurprisingly, multiple halogenated maleimide derivatives worked well using this protocol, providing **4L** with 60% yield. Most importantly, the newly developed radical diarylthiolation strategy was well proved in two-fold reactions, **4m**, **4n** and **4o** were provided in good yield. The above results demonstrated the distinctive advantage of our double C-S bonds formation protocol. Unfortunately, when we extended the substrates to other alkenes, such as styrene and methyl acrylate, no products were observed. We also screened copper catalysts, ligands, reaction solvents and reaction temperatures, but no beneficial results were obtained. These results in turn confirmed the unique chemical reactivity of maleimide.

**SCHEME 4:**
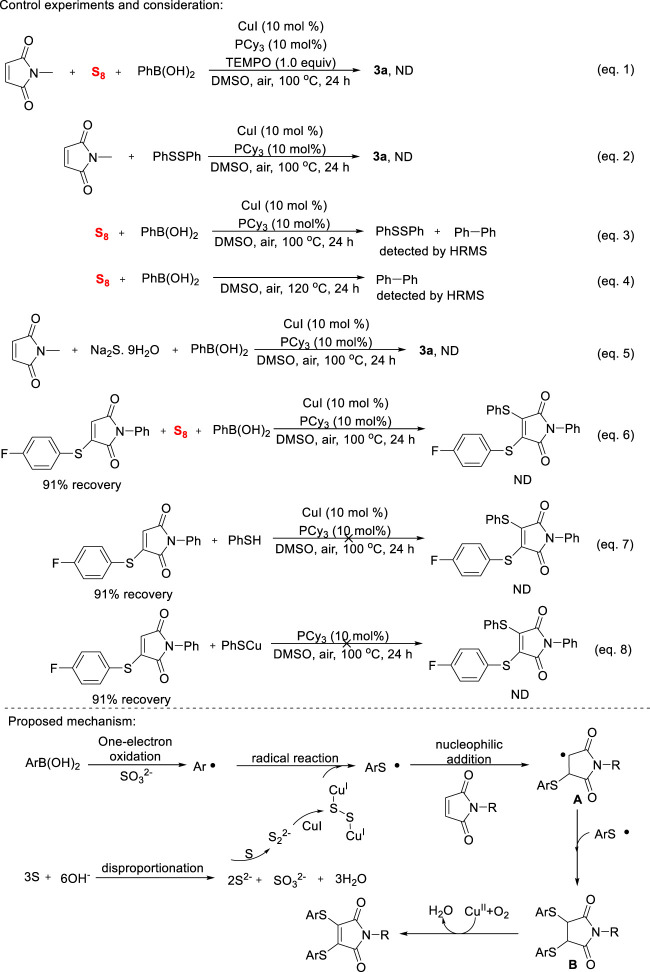
Mechanism of study and proposal.

Control experiments were performed to elucidate the detailed mechanism of copper-catalyzed diarylthiolation of maleimides (Scheme 4). As expected, the addition of TEMPO promptly extinguished the transformation (Eq. 1), which indicates that a multi-component reaction occurs through a radical reaction. The mixture of *N*-methyl maleimide with diphenyl disulfide did not provide the oxidative thiolated product (Eq. 2), which suggests that disulfides were not competent intermediate. In the reaction of sulfur powder and phenyl boronic acid under the optimized reaction conditions (Eq. 3), a mixture of diphenyl disulfide and biphenyl were detected by HRMS, this result showed that aryl and thiyl radical were formed during the reaction progress. Surprisingly, the reaction mentioned above was conducted in the absence of copper salt (Eq. 4), a large amount of biphenyl is still obtained and confirmed by HRMS. This reaction shows that phenylboronic acid was oxidized by sulfur powder to form phenyl radicals. In general, sulfide anions and high-valence sulfates could be formed by the disproportionation of sulfur powder under base conditions. However, our reaction conditions are almost neutral. When Na_2_S was used as a sulfur source in reaction mixture (Eq. 5), **3a** product was not observed on TLC. Although the exact mechanism is not clear at the present research stage, S powder does play the role of substrate and internal oxidant in this transformation. Finally, the reaction did not occur when a mixture of 3-((4-fluorophenyl)thio)-1-phenyl-1H-pyrrole-2,5-dione, S power and phenylboronic acid (Eq. 6), the step-by-step oxidation thiolation of maleimides could be excluded. We also tried to use ArSH or ArSCu as substrate, however, no arylthiolation reaction was observed in both cases (Eqs 7, 8). According to this mechanistic investigation and previous related literature, a reasonable mechanism was proposed in Scheme 4. S powder undergoes disproportionation to produce sulfide and sulfate (Zhang et al., 2014), the former coordinated with copper salts to deliver sulfur copper species (Ravi et al., 2016), the latter oxidizes the aryl boronic acids to obtain aryl radicals (Dutta et al., 2020). The formed aryl radicals would preferentially react with sulfur copper to give arylthiyl radical (Jiang et al., 2009), and then nucleophilic addition with maleimide provided the carbon radical **A**. Subsequently, the highly selective capture reaction between A and arylthiyl radical afforded intermediate **B**, followed by copper-catalyzed oxidative dehydrogenation step to give the anticipated product.

**FIGURE 1:**
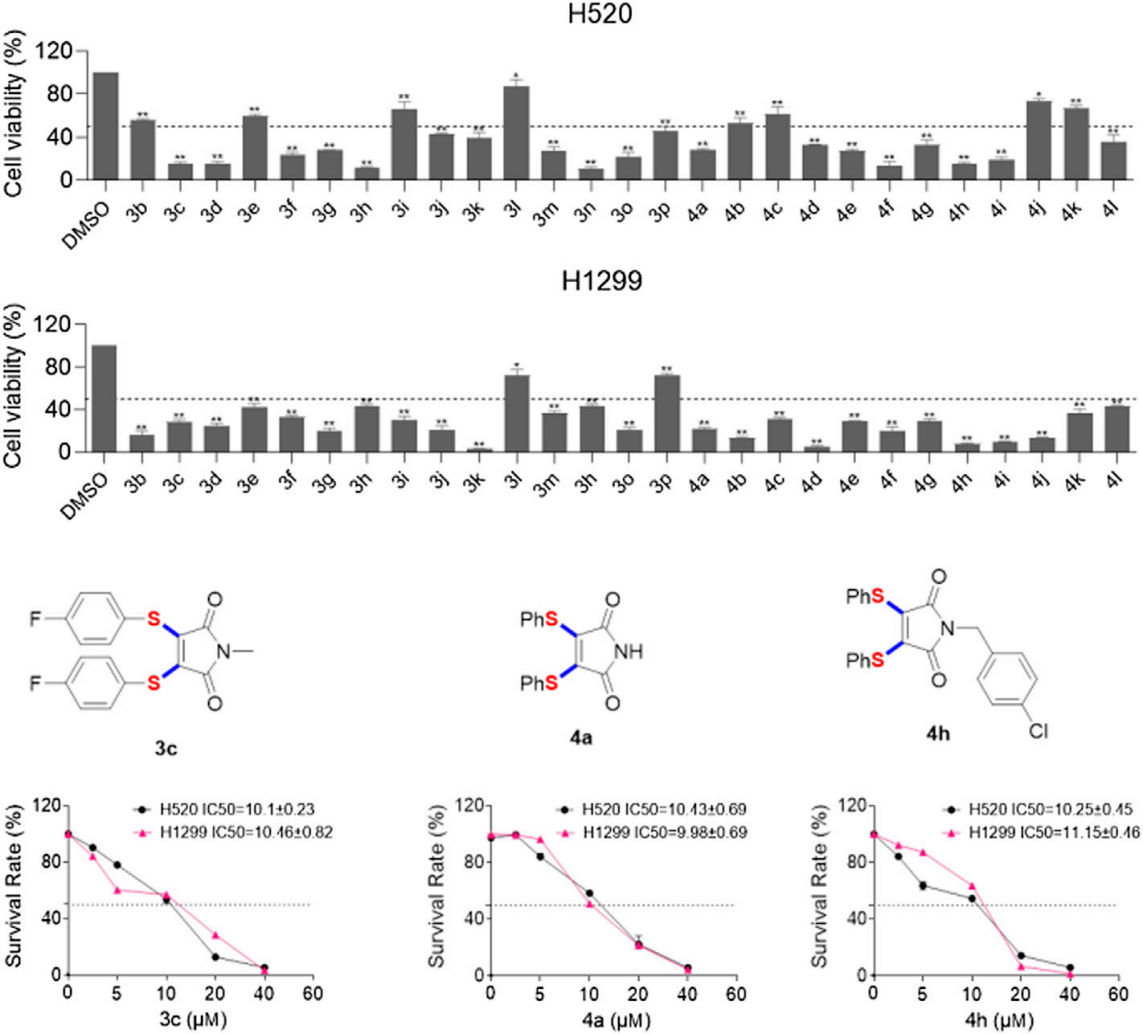
Anticancer activity of 3,4-diarylthiolated maleimides.

In addition, we further determined the cytotoxicity of these 27 compounds on human lung cancer cells (Figure 1), H520 and H1299 were treated with these compounds at the same concentration (20 μM) for 48 h. As shown in Figure 1, 17 of these compounds can reduce cell viability of H520 cells to less than fifty percent. As for H1299 cells, there are 25 compounds can reduce cell viability to below 50%. Remarkably, **4c** (H520 IC_50_ = 10.1μM; H1299 IC_50_ = 10.5 μM), **4a** (H520 IC_50_ = 10.4μM; H1299 IC_50_ = 9.98 μM) and **4h** (H520 IC_50_ = 10.2μM; H1299 IC_50_ = 11.1 μM) displayed potent cell growth inhibition activity of H520 and H1299. Then, we explored the relationship between toxicity and concentration of these three compounds. The results showed that treatment with these compounds caused a dose-dependent increase in cytotoxicity.

## 3 Conclusion

In conclusion, we have established a concise and feasible protocol to prepare diarylthiolated maleimides through copper-catalyzed radical cascade reaction of maleimides with S powder and aryl boronic acids. This novel transformation consists of single-electron oxidation of aryl boronic acids by sulfur powder, radical diarylthiolation and oxidative dehydrogenation steps. Sulfur powder is not only used as a substrate of multi-component thiolation reaction, but also work as an internal oxidant. The prepared 3,4-diarylthiolated maleimides, such as **3c**, **4a** and **4h** showed excellent anticancer of human lung cancer cells (H520 and H1299).

## 4 Materials and methods

### 4.1 Cell culture and reagents

Typan blue was purchased from Solarbio (Beijing, China), the human lung cancer H520 and H1299 cell lines were purchased from the Cell Bank of Type Culture Collection of the Chinese Academy of Sciences, Shanghai, China. The cells were routinely cultured in RPMI 1640 medium (Gibco, Eggenstein, Germany) containing 10% heat-inactivated fetal bovine serum. Cells were propagated in a humidified cell incubator with an atmosphere of 5% CO_2_ at 37°C.

### 4.2 Cell viability assay

H520 and H1299 cell lines were seeded in 6-well plates and incubated overnight in the incubator with an atmosphere of 5% CO_2_ at 37°C. The cells were then treated with different compounds for 48 h. After that, the cell viability was determined using trypan blue exclusion and then indicated the percentage of viable cells relative to DMSO-treated cells.

### 4.3 Colony formation assay

The cells were seeded in 6-well plates and incubated overnight in the incubator with an atmosphere of 5% CO_2_ at 37°C. After cultured for 1 week, the cells were washed with phosphate-buffered saline (PBS) twice when visible colonies were formed. After being fixed with 4% paraformaldehyde for 20 min, cells were washed with PBS and stained for 15 min. At last, cells were washed with PBS twice and the colonies were counted under ordinary optical microscope.

## Data Availability

The original contributions presented in the study are included in the article/Supplementary Material, further inquiries can be directed to the corresponding author.
