# Supplementary material for: Anticancer-active 3,4-diarylthiolated maleimides synthesis via three-component radical diarylthiolation
Source: Front Chem. 2022 Nov 25;10:1089860. doi: 10.3389/fchem.2022.1089860 (PMC9732252; doi:10.3389/fchem.2022.1089860)
Supplement: Supplementary file 1 [file DataSheet1.docx]

Supporting Information

**Anticancer-Active 3,4-diarylthiolated Maleimides Synthesis via Three-Component Radical Bisarylthiolation**

**Limei Wang,^1†^ Zhuo Li,^2†^ Zhehan Ma,^3^ Kedi Xia,^4^ Wenyu Wang,^5^ Wenchang Yu^4*^**

^1^Department of Clinical Pharmacy, Jilin Province FAW General Hospital, Changchun 130000, China

^2^Department of Pharmacy, Affiliated Hospital of Changchun University of Chinese Medicine, Changchun 130000, China

^3^Department of Otolaryngology，Jilin Province FAW General Hospital, Changchun 130000, China

^4^Department of Pharmacy, Jilin Province FAW General Hospital, Changchun 130000, China

^5^Department of Thoracic Surgery, Jilin Province FAW General Hospital, Changchun 130000, China

***Corresponding email:** 1151614322@qq.com

**Table of Contents**

**(1) General considerations, experimental data………S2-S21**

**(2) References for known compounds……………………S22**

**(3) ^1^H, ^13^C and ^19^F NMR spectra of products………S23-S55**

**General Information**

*N*-substituent Maleimides^1^ were prepared according to the reported procedures. ^1^H and ^13^C spectra of known compounds were in accordance with those described in the literatures. All other reagents were purchased from TCI, Sigma-Aldrich, Alfa Aesar, Acros, and Meryer and used without further purification. ^1^H NMR (500 MHz), ^13^C NMR (125 MHz) and ^19^F NMR (470 MHz) spectra were recorded in CDCl_3_ and DMSO-D6 solutions using a Burker AVANCE 500 spectrometer. High-resolution mass spectra were recorded on an ESI-Q-TOF mass spectrometer. Analysis of crude reaction mixture was done on the Varian 4000 GC/MS and 1200 LC. All reactions were conducted using standard Schlenk techniques. Column chromatography was performed using EM silica gel 60 (300−400 m).

**General Procedure of Copper-Catalyzed Oxidative Bisthiolation of Maleimides with S Powder and Arylboronic Acids：**

A 25 mL Schlenk tube equipped with a stir bar was charged with S_8_ powder (0.6 mmol), arylboronic acids (0.6 mmol), maleimide (0.2 mmol), CuI (0.02 mol), tricyclohexyl phosphine (0.02 mmol) and 2 mL DMSO, then, the tube was fitted with Teflon screwcap. The reaction mixture was stirred at 120 ℃ (aluminium block heating mantle) for 24 h. After the reaction, 10 ml ethyl acetate and 30 ml water were added for extraction, dried with sodium sulfate for half an hour, then dried and concentrated under reduced pressure. The residue was then purified by flash chromatography on silica gel to provide the corresponding product.

**5mmol scale-up reaction:**

A 150 mL Schlenk tube equipped with a stir bar was charged with S_8_ powder (15 mmol), phenylboronic acid (15 mmol), *N*-Phenylmaleimide (5.0 mmol), CuI (0.5 mol), tricyclohexyl phosphine (0.5 mmol), and 50 mL DMSO, then, the tube was fitted with Teflon screwcap. The reaction mixture was stirred at 120 ℃ (oil bath pan) for 24 h. After the reaction, 50 ml ethyl acetate and 100 ml water were added for extraction, dried with sodium sulfate for half an hour, then dried and concentrated under reduced pressure. The residue was then purified by flash chromatography on silica gel to provide the corresponding 1-phenyl-3,4-bis(phenylthio)-1H-pyrrole-2,5-dione (79%, isolated yield).

**Mechanism investigation:**

A 25 mL Schlenk tube equipped with a stir bar was charged with S_8_ powder (0.6 mmol), phenylboronic acid (0.6 mmol), *N*-methly maleimide (0.2 mmol), CuI (0.02 mol), tricyclohexyl phosphine (0.02 mmol), TEMPO (0.2 mmol) and 2 mL DMSO, then, the tube was fitted with Teflon screwcap. The reaction mixture was stirred at 120 ℃ (aluminium block heating mantle) for 24 h. After the reaction, 10 ml ethyl acetate and 30 ml water were added for extraction, the absence of **3a** point was confirmed by TLC. This result includes the involvement of a radical species in the reaction progress.

A 25 mL Schlenk tube equipped with a stir bar was charged with diphenyl disulfide (0.6 mmol), *N*-methly maleimide (0.2 mmol), CuI (0.02 mol), tricyclohexyl phosphine (0.02 mmol) and 2 mL DMSO, then, the tube was fitted with Teflon screwcap. The reaction mixture was stirred at 120 ℃ (aluminium block heating mantle) for 24 h. After the reaction, 10 ml ethyl acetate and 30 ml water were added for extraction, dried with sodium sulfate for half an hour, then dried and concentrated under reduced pressure. After the reaction, the absence of **3a** point was confirmed by TLC. This reaction process shows that disulfide is not a reaction intermediate.

A 25 mL Schlenk tube equipped with a stir bar was charged with S_8_ powder (0.6 mmol), phenylboronic acid (0.6 mmol), CuI (0.02 mol), tricyclohexyl phosphine (0.02 mmol) and 2 mL DMSO, then, the tube was fitted with Teflon screwcap. The reaction mixture was stirred at 120 ℃ (aluminium block heating mantle) for 24 h. After the reaction, 10 ml ethyl acetate and 30 ml water were added for extraction, diphenyl disulfide and biphenyl was detected by HRMS. This result demonstrated that arylthiyl radical and aryl radical intermediates are formed during the reaction progress.


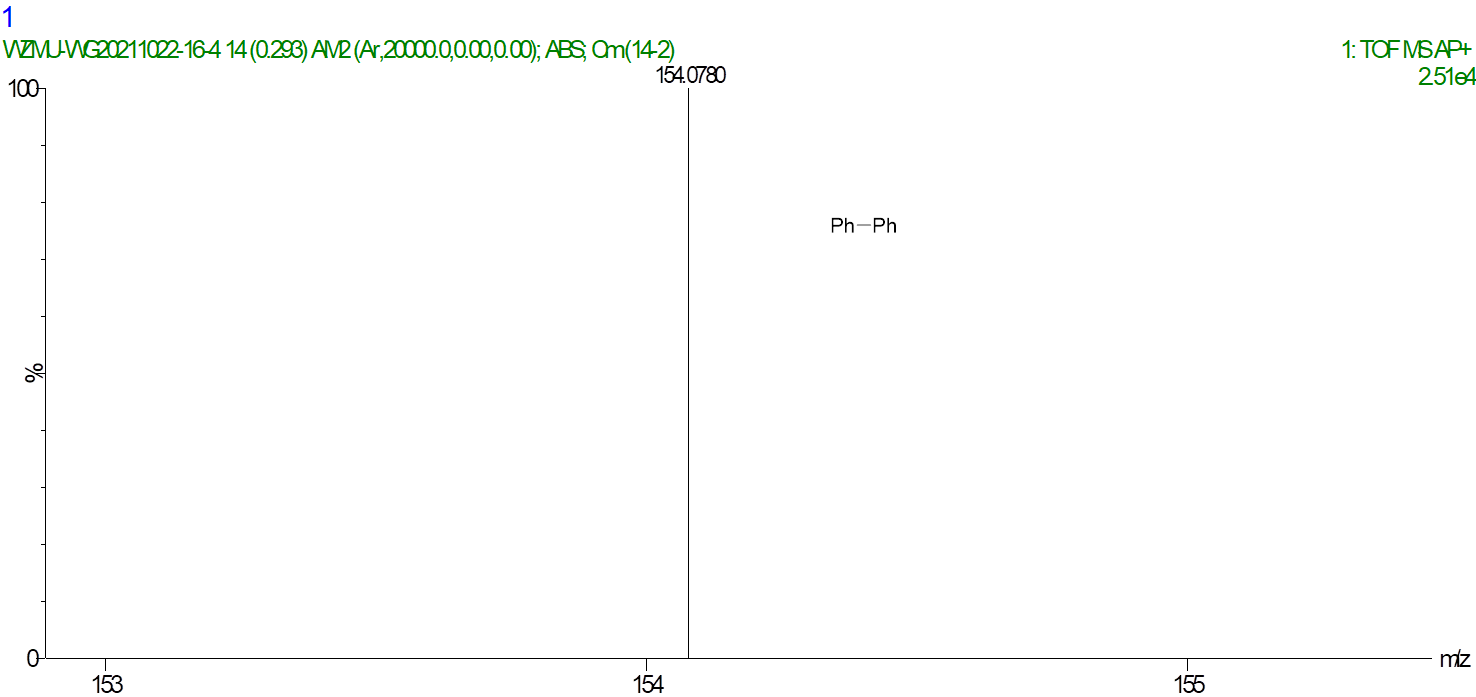


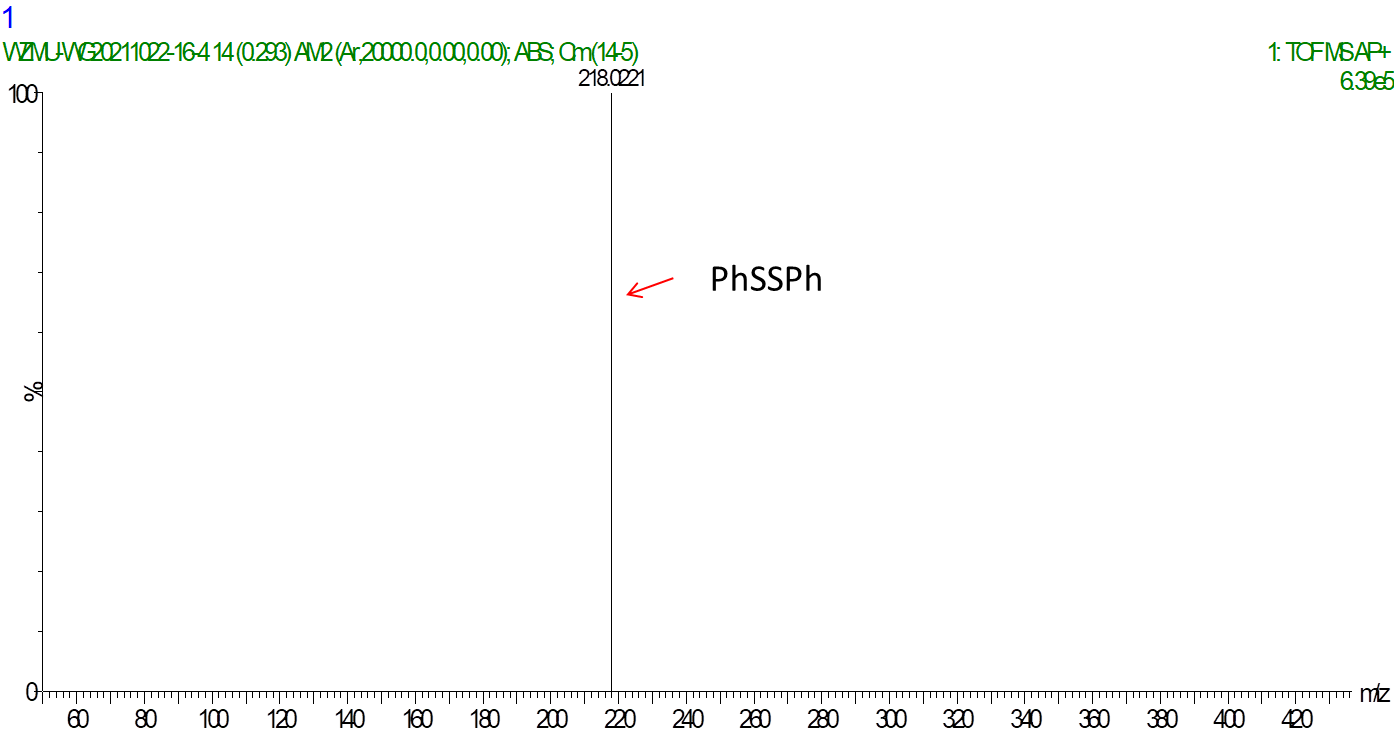

A 25 mL Schlenk tube equipped with a stir bar was charged with S_8_ powder (0.6 mmol), phenylboronic acid (0.6 mmol) and 2 mL DMSO, then, the tube was fitted with Teflon screwcap. The reaction mixture was stirred at 120 ℃ (aluminium block heating mantle) for 24 h. After the reaction, 10 ml ethyl acetate and 30 ml water were added for extraction, diphenyl disulfide and biphenyl was detected by HRMS. This result demonstrated that aryl radical intermediate is formed during the reaction progress, copper catalyst palys an important role in the activation of sulfur powder.

A 25 mL Schlenk tube equipped with a stir bar was charged with sodium sulfide nonahydrate (0.6 mmol), phenylboronic acid (0.6 mmol), *N*-methly maleimide (0.2 mmol), CuI (0.02 mol), tricyclohexyl phosphine (0.02 mmol) and 2 mL DMSO, then, the tube was fitted with Teflon screwcap. The reaction mixture was stirred at 120 ℃ (aluminium block heating mantle) for 24 h. After the reaction, 10 ml ethyl acetate and 30 ml water were added for extraction, the absence of **3a** point was confirmed by TLC. This result includes sulfur powder provides sulfur anions in the cross-coupling reactions, the generated the sulfate works as an oxidant is also extremely important.

A 25 mL Schlenk tube equipped with a stir bar was charged with S_8_ powder (0.6 mmol), phenylboronic acid (0.6 mmol), 3-((4-fluorophenyl)thio)-1-phenyl-1H-pyrrole-2,5-dione (0.2 mmol), CuI (0.02 mol), tricyclohexyl phosphine (0.02 mmol) and 2 mL DMSO, then, the tube was fitted with Teflon screwcap. The reaction mixture was stirred at 120 ℃ (aluminium block heating mantle) for 24 h. After the reaction, 10 ml ethyl acetate and 30 ml water were added for extraction, the corresponding product was not detected by TLC and HRMS, except with 91% recovery of starting material. This result indicates that the bisthiolation reactions is not performed with step-by-step thiolation progress.


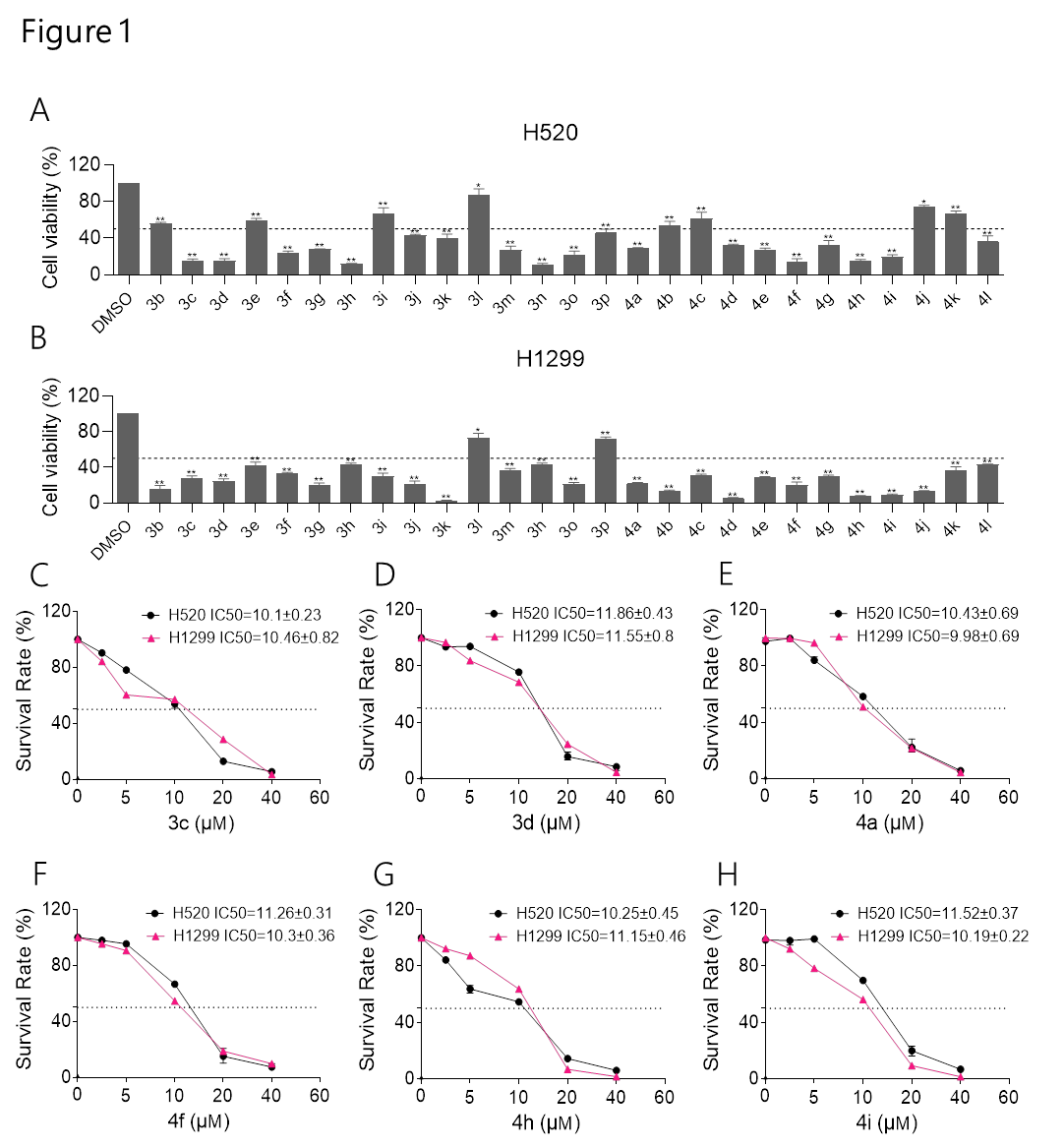


**Compounds have significant cytotoxic effect on lung cancer cells**

We further determined the cytotoxic effect of these 27 compounds on human lung cancer cells, H520 and H1299 were treated with the same concentration (20 μM) of these compounds for 48 h. As shown in Figure 1A-B, 17 of these compounds can reduce cell viability of H520 cells to less than fifty percent. As for H1299 cells, 25 compounds can reduce cell viability to less than fifty percent. Among these 27 compounds, we selected the 6 most effective compounds 3c, 3d, 4a, 4f, 4h and 4i for further study. Then, we explored the relationship between toxicity and concentration of these six compounds. The results showed that treatment with these compounds caused a dose-dependent increase in cytotoxicity (Figure 1C-H).

**
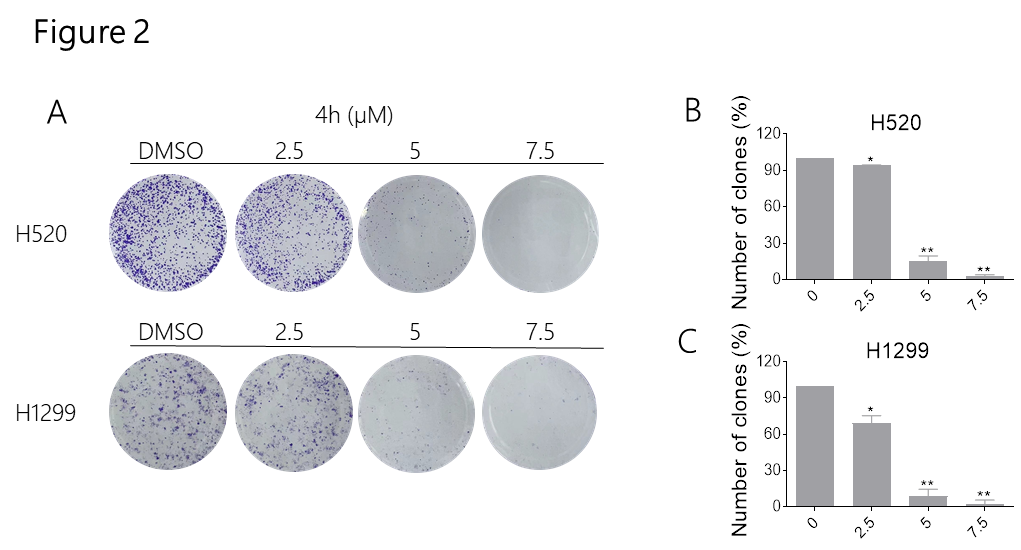
**

**4h suppressed the colony formation ability of lung cancer cells**

To evaluate the potential long-term effect of these compounds on human lung cancer cells, we selected the compound 4h and performed the colony formation assay. 4h caused a significant concentration-dependent decrease of colony numbers, in H520 and H1299 cells, compared with the control group (Figure 2A-C). These data suggest that 4h significantly suppresses the growth of lung cancer cells.

**Characterization of Products in Details:**

**1-methyl-3,4-bis(phenylthio)-1H-pyrrole-2,5-dione**

Following the general procedure, using (petroleum ether : EtOAc = 9 : 1) as the eluant afforded a yellow liquid (53.6 mg, 82% yield). **^1^H NMR** (500 MHz, CDCl_3_): δ 7.29-7.20 (m, 10H), 3.00 (s, 3H); **^13^C NMR** (125MHz, CDCl_3_): δ 166.9, 135.9, 132.0, 129.1, 129.0, 128.4, 24.7; **HRMS** (ESI): calcd for C_17_H_14_NO_2_S_2_ [M + H]^+^ 328.0466, found 328.0468.

**1-methyl-3,4-bis(p-tolylthio)-1H-pyrrole-2,5-dione**

Following the general procedure, using (petroleum ether : EtOAc = 9 : 1) as the eluant afforded a yellow solid (54.6 mg, 77% yield), Mp = 66-67℃. **^1^H NMR** (500 MHz, CDCl_3_): δ 7.20 (d, *J* = 8.1 Hz, 4H), 7.09 (d, *J* = 8.0 Hz, 4H), 2.96 (s, 3H), 2.33 (s, 6H); **^13^C NMR** (125MHz, CDCl_3_): δ 166.8, 138.9, 136.2, 132.5, 130.0, 129.8, 125.7, 115.1, 24.6, 21.3; **HRMS** (ESI): calcd for C_19_H_17_NO_2_NaS_2_ [M + Na]^+^ 378.0598, found 378.0608.

**3,4-bis((4-fluorophenyl)thio)-1-methyl-1H-pyrrole-2,5-dione**

Following the general procedure, using (petroleum ether : EtOAc = 9 : 1) as the eluant afforded a yellow solid (58.1 mg, 80% yield), Mp = 80-81℃. **^1^H NMR** (400 MHz, CDCl_3_): δ 7.34-7.30 (m, 4H), 7.03 (t, *J* = 8.6 Hz, 4H), 3.01 (s, 3H); **^13^C NMR** (100MHz, CDCl_3_): δ 166.6, 163.2 (d, *J* = 250.0 Hz), 135.8, 134.6 (d, *J* = 8.5 Hz), 124.1 (d, *J* = 3.4 Hz), 116.4 (d, *J* = 22.2 Hz), 24.7; **^19^F NMR** (375 MHz, CDCl_3_): δ -111.4 (s, 1F); **HRMS** (ESI): calcd for C_17_H_12_NO_2_F_2_S_2_ [M + H]^+^ 364.0278, found 364.0270.

**3,4-bis((4-chlorophenyl)thio)-1-methyl-1H-pyrrole-2,5-dione**

Following the general procedure, using (petroleum ether : EtOAc = 9 : 1) as the eluant afforded a yellow solid (61.6 mg, 78% yield), Mp = 84-85℃. **^1^H NMR** (400 MHz, CDCl_3_): δ 7.27 (d, *J* = 8.5 Hz, 4H), 7.17 (d, *J* = 8.5 Hz, 4H), 3.04 (s, 3H); **^13^C NMR** (100MHz, CDCl_3_): δ 166.6, 135.4, 134.9, 133.2, 129.3, 127.2, 24.8; **HRMS** (ESI): calcd for C_17_H_12_NO_2_S_2_Cl_2_ [M + H]^+^ 395.9687, found 395.9689.

**3,4-bis((2-chlorophenyl)thio)-1-methyl-1H-pyrrole-2,5-dione**

Following the general procedure, using (petroleum ether : EtOAc = 9 : 1) as the eluant afforded a yellow liquid (35.5 mg, 45% yield). **^1^H NMR** (400 MHz, CDCl_3_): δ 7.38 (t, *J* = 9.1 Hz, 4H), 7.29 (t, *J* = 7.2 Hz, 2H), 7.22 (t, *J* = 7.5 Hz, 2H), 3.04 (s, 3H); **^13^C NMR** (100MHz, CDCl_3_): δ 166.4, 136.6, 135.5, 133.9, 130.2, 130.1, 128.3, 127.3, 24.8; **HRMS** (ESI): calcd for C_17_H_12_NO_2_S_2_Cl_2_ [M + H]^+^ 395.9687, found 395.9689.

**3,4-bis((3-chlorophenyl)thio)-1-methyl-1H-pyrrole-2,5-dione**

Following the general procedure, using (petroleum ether : EtOAc = 9 : 1) as the eluant afforded a yellow liquid (52.1 mg, 66% yield). **^1^H NMR** (400 MHz, CDCl_3_): δ 7.29 (d, *J* = 8.0 Hz, 2H), 7.21 (t, *J* = 7.9 Hz, 2H), 7.08 (t, *J* = 1.9 Hz, 2H), 7.03 (d, *J* = 7.8 Hz, 2H), 3.09 (s, 3H); **^13^C NMR** (100MHz, CDCl_3_): δ 166.9, 134.8, 134.8, 130.9, 129.9, 129.4, 128.7, 24.9; **HRMS** (ESI): calcd for C_17_H_12_NO_2_S_2_Cl_2_ [M + H]^+^ 395.9687, found 395.9689.

**3,4-bis((4-bromophenyl)thio)-1-methyl-1H-pyrrole-2,5-dione**

Following the general procedure, using (petroleum ether : EtOAc = 9 : 1) as the eluant afforded a yellow solid (67.5 mg, 70% yield), Mp = 74-75℃. **^1^H NMR** (500 MHz, CDCl_3_): δ 7.38 (d, *J*=8.45 Hz, 4H), 7.05 (d, *J*=8.45 Hz, 4H), 3.02 (s, 3H); **^13^C NMR** (125MHz, CDCl_3_): δ 166.6, 135.3, 133.3, 132.1, 127.8, 122.9, 24.8; **HRMS** (ESI): calcd for C_17_H_12_NO_2_S_2_Br_2_ [M + H]^+^ 483.8676, found 483.8674.

**3,4-bis((4-iodophenyl)thio)-1-methyl-1H-pyrrole-2,5-dione**

Following the general procedure, using (petroleum ether : EtOAc = 9 : 1) as the eluant afforded a yellow solid (76.4 mg, 66% yield), Mp = 97-98℃. **^1^H NMR** (400 MHz, CDCl_3_): δ 7.61 (d, *J* = 8.5 Hz, 4H), 6.92 (d, *J* = 8.4 Hz, 4H), 3.06 (s, 3H); **^13^C NMR** (100MHz, CDCl_3_): δ 166.7, 138.1, 135.1, 133.2, 128.6, 94.3, 24.9; **HRMS** (ESI): calcd for C_17_H_12_NO_2_S_2_I_2_ [M + H]^+^ 579.8399, found 579.8404.

1-methyl-3,4-bis((4-(trifluoromethyl)phenyl)thio)-1H-pyrrole-2,5-dione

Following the general procedure, using (petroleum ether : EtOAc = 9 : 1) as the eluant afforded a yellow solid (75.0 mg, 81% yield), Mp = 68-69℃. **^1^H NMR** (500 MHz, CDCl_3_): δ 7.49 (d, *J* = 8.2 Hz, 4H), 7.18 (d, *J* = 8.1 Hz, 4H), 3.12 (s,3H)；**^13^C NMR** (125MHz, CDCl_3_): δ 166.6, 133.8 (d, *J* = 215.5 Hz), 130.9, 130.3 (d, *J* = 32.9 Hz), 125.7 (q, *J* = 3.8 Hz), 123.6 (d, *J* = 272.4 Hz), 24.9; **^19^F NMR** (375 MHz, CDCl_3_): δ -62.7 (s, 3F); **HRMS** (ESI): calcd for C_19_H_12_NO_2_F_6_S_2_ [M + H]^+^ 464.0214, found 464.0205.

**dimethyl 4,4'-((1-methyl-2,5-dioxo-2,5-dihydro-1H-pyrrole-3,4-diyl)bis(sulfanediyl))dibenzoate**

Following the general procedure, using (petroleum ether : EtOAc = 9 : 1) as the eluant afforded a yellow solid (52.3 mg, 59% yield), Mp = 98-99℃. **^1^H NMR** (500 MHz, CDCl_3_): δ 7.87 (d, *J*=8.40 Hz, 4H), 7.14 (d, *J*=8.46 Hz, 4H), 3.92 (s, 6H), 3.06 (s, 3H); **^13^C NMR** (125MHz, CDCl_3_): δ 166.7, 166.3, 137.8, 136.4, 135.4, 134.4, 130.7, 130.5, 130.4, 130.2, 129.8, 127.5, 126.0, 52.4, 24.9; **HRMS** (ESI): calcd for C_21_H_17_NO_6_NaS_2_ [M + Na]^+^ 466.0395, found 466.0395.

**1-methyl-3,4-bis(naphthalen-1-ylthio)-1H-pyrrole-2,5-dione**

Following the general procedure, using (petroleum ether : EtOAc = 9 : 1) as the eluant afforded a yellow liquid (68.3 mg, 80% yield). **^1^H NMR** (400 MHz, CDCl_3_): δ 8.07-8.05 (m, 2H), 7.83-7.80 (m, 4H), 7.61 (dd, *J* = 7.3, 1.2 Hz, 2H), 7.54 (tt, *J* = 6.9, 5.2 Hz, 4H), 7.36 (dd, *J* = 8.2, 7.2 Hz, 2H), 2.94 (s, 3H); **^13^C NMR** (100MHz, CDCl_3_): δ 166.7, 135.8, 134.3, 133.9, 133.1, 130.3, 128.8, 127.2, 126.4, 126.0, 125.4, 125.1, 24.6; **HRMS** (ESI): calcd for C_25_H_17_NO_2_NaS_2_ [M + Na]^+^ 450.0598, found 450.0599.

**1-methyl-3,4-bis((4-(trimethylsilyl)phenyl)thio)-1H-pyrrole-2,5-dione**

Following the general procedure, using (petroleum ether : EtOAc = 9 : 1) as the eluant afforded a yellow solid (56.5 mg, 60% yield), Mp = 74-75℃. **^1^H NMR** (400 MHz, CDCl_3_): δ 7.35 (d, *J* = 8.0 Hz, 4H), 7.06 (d, *J* = 8.1 Hz, 4H), 3.09 (s, 3H), 0.30 (s, 18H)；**^13^C NMR** (100MHz, CDCl_3_): δ 167.3, 140.6, 135.1, 133.7, 130.3, 129.4, 24.8, -1.2；**HRMS** (ESI): calcd for C_23_H_30_NO_2_S_2_Si_2_ [M + H]^+^ 472.1256, found 472.1252.

**3,4-bis([1,1'-biphenyl]-4-ylthio)-1-methyl-1H-pyrrole-2,5-dione**

Following the general procedure, using (petroleum ether : EtOAc = 9 : 1) as the eluant afforded a yellow solid (67.1 mg, 70% yield), Mp = 156-157℃. **^1^H NMR** (400 MHz, CDCl_3_): δ 7.60 (d, *J* = 7.7 Hz, 4H), 7.52-7.47 (m, 8H), 7.41 (t, *J* = 7.3 Hz, 2H), 7.30-7.27 (m, 4H), 3.10 (s, 1H); **^13^C NMR** (100MHz, CDCl_3_): δ 167.2, 141.3, 140.0, 135.6, 132.1, 128.9, 127.9, 127.8, 127.6, 127.1, 24.8; **HRMS** (ESI): calcd for C_29_H_21_NO_2_NaS_2_ [M + Na]^+^ 502.0911, found 502.0909.

**1-methyl-3,4-bis((4-phenoxyphenyl)thio)-1H-pyrrole-2,5-dione**

Following the general procedure, using (petroleum ether : EtOAc = 9 : 1) as the eluant afforded a yellow liquid (64.4 mg, 63% yield). **^1^H NMR** (500 MHz, CDCl_3_): δ 7.36-7.24 (m, 10H), 7.14 (t, *J* = 7.3 Hz, 2H), 7.02-6.98 (m, 4H), 6.92-6.88 (m, 4H), 2.99 (s, 3H); **^13^C NMR** (125MHz, CDCl_3_): δ 166.8, 158.4, 156.2, 136.1, 134.5, 129.9, 124.1, 122.3, 119.5, 118.9, 24.6; **HRMS** (ESI): calcd for C_29_H_21_NO_4_NaS_2_ [M + Na]^+^ 534.0810, found 534.0807.

**3,4-bis((3-methoxyphenyl)thio)-1-methyl-1H-pyrrole-2,5-dione**

Following the general procedure, using (petroleum ether : EtOAc = 9 : 1) as the eluant afforded a yellow solid (58.1 mg, 75% yield), Mp = 78-79℃. **^1^H NMR** (500 MHz, CDCl_3_): δ 7.14 (t, *J*=8.10 Hz, 2H), 6.80 (dd, *J*=8.30, 2.20 Hz, 2H), 6.74 (d, *J*=7.70 Hz, 2H), 6.64-6.63(m, 2H), 3.74 (s, 6H), 3.03(s, 3H); **^13^C NMR** (125MHz, CDCl_3_): δ 167.1, 159.6, 135.5, 129.8, 129.6, 123.9, 116.6, 114.3, 55.3, 24.7;

**HRMS** (ESI): calcd for C_19_H_17_NO_4_NaS_2_ [M + Na]^+^ 410.0497, found 410.0500.

**3,4-bis(mesitylthio)-1-methyl-1H-pyrrole-2,5-dione**

Following the general procedure, using (petroleum ether : EtOAc = 9 : 1) as the eluant afforded a yellow solid (38.6 mg, 47% yield), Mp = 96-97℃. **^1^H NMR** (400 MHz, CDCl_3_): δ 6.96 (s, 4H), 2.90 (s, 3H), 2.45 (s, 12H), 2.31 (s, 6H); **^13^C NMR** (100MHz, CDCl_3_): δ 166.3, 143.2, 139.9, 133.7, 129.2, 123.8, 24.4, 22.1, 21.2; **HRMS** (ESI): calcd for C_23_H_25_NO_2_NaS_2_ [M + H]^+^ 434.1224, found 434.1225.

**3,4-bis(phenylthio)-1H-pyrrole-2,5-dione**

Following the general procedure, using (petroleum ether : EtOAc = 9 : 1) as the eluant afforded a yellow solid (26.3 mg, 42% yield), Mp = 94-95℃. **^1^H NMR** (500 MHz, CDCl_3_): δ 7.75 (brs, 1H), 7.29-7.23 (m, 6H), 7.19-7.17 (m, 4H); **^13^C NMR** (125MHz, CDCl_3_): δ 166.3, 136.7, 131.9, 129.0, 128.8, 128.5; **HRMS** (ESI): calcd for C_16_H_12_NO_2_S_2_ [M + H]^+^ 314.0309, found 314.0314.

**1-cyclohexyl-3,4-bis(phenylthio)-1H-pyrrole-2,5-dione**

Following the general procedure, using (petroleum ether : EtOAc = 9 : 1) as the eluant afforded a yellow solid (64.8 mg, 82% yield), Mp = 76-77℃. **^1^H NMR** (500 MHz, CDCl_3_): δ 7.26-7.17 (m, 10H), 3.92-3.87 (m, 1H), 2.05-1.97 (m, 2H), 1.78 (d, *J*=12.80 Hz, 2H), 1.66-1.59 (m, 3H), 1.29-1.14 (m, 3H); **^13^C NMR** (125MHz, CDCl_3_): δ 166.8, 135.4, 131.8, 129.2, 128.9, 128.3, 51.8, 29.9, 25.9, 25.0; **HRMS** (ESI): calcd for C_22_H_22_NO_2_S_2_ [M + H]^+^ 396.1092, found 396.1093.

**1-phenyl-3,4-bis(phenylthio)-1H-pyrrole-2,5-dione**

Following the general procedure, using (petroleum ether : EtOAc = 9 : 1) as the eluant afforded a yellow solid (63.0 mg, 81% yield), Mp = 80-81℃. **^1^H NMR** (500 MHz, CDCl_3_): δ 7.61-7.28 (m, 15H); **^13^C NMR** (125MHz, CDCl_3_): δ 165.7, 135.9, 132.3, 131.5, 129.1, 129.1, 128.9, 128.7, 127.9, 125.9; **HRMS** (ESI): calcd for C_22_H_16_NO_2_S_2_ [M + H]^+^ 390.0622, found 390.0625.

**1-(4-methylbenzyl)-3,4-bis(phenylthio)-1H-pyrrole-2,5-dione**

Following the general procedure, using (petroleum ether : EtOAc = 9 : 1) as the eluant afforded a yellow liquid (62.5 mg, 75% yield). **^1^H NMR** (500 MHz, CDCl_3_): δ 7.34-7.28 (m, 8H), 7.26-7.23 (m, 4H), 7.19-7.17 (m, 2H), 4.60 (s, 2H), 2.31 (s, 3H); **^13^C NMR** (125MHz, CDCl_3_): δ 166.5, 137.7, 135.8, 133.1, 131.9, 130.2, 129.3, 129.1, 128.9, 128.8, 128.6, 128.4, 42.2, 21.1; **HRMS** (ESI): calcd for C_24_H_19_NO_2_NaS_2_ [M + Na]^+^ 440.0755, found 440.0754.

**1-(4-fluorobenzyl)-3,4-bis(phenylthio)-1H-pyrrole-2,5-dione**

Following the general procedure, using (petroleum ether : EtOAc = 9 : 1) as the eluant afforded a yellow solid (58.9 mg, 70% yield), Mp = 60-61℃. **^1^H NMR** (400 MHz, CDCl_3_): δ 7.40-7.23 (m, 12H), 7.03 (t, *J* = 8.7 Hz, 2H), 4.66 (s, 2H); **^13^C NMR** (100MHz, CDCl_3_): δ 166.5, 162.5 (d, *J* = 246.7 Hz), 135.8, 132.0, 130.8 (d, *J* = 8.2 Hz), 129.1, 129.0, 128.5, 115.6 (d, *J* = 21.5 Hz), 41.8; **^19^F NMR** (375 MHz, CDCl_3_): δ -113.9 (s, 1F); **HRMS** (ESI): calcd for C_23_H_16_NO_2_NaS_2_F [M + Na]^+^ 444.0504, found 444.0507.

**3,4-bis(phenylthio)-1-(4-(trifluoromethyl)benzyl)-1H-pyrrole-2,5-dione**

Following the general procedure, using (petroleum ether : EtOAc = 9 : 1) as the eluant afforded a yellow solid (62.2 mg, 66% yield), Mp = 81-82℃. **^1^H NMR** (400 MHz, CDCl_3_): δ 7.61 (d, *J* = 8.0 Hz, 2H), 7.49 (d, *J* = 8.0 Hz, 2H), 7.36-7.24 (m, 10H), 4.74 (s, 2H); **^13^C NMR** (100MHz, CDCl_3_): δ 166.4, 139.8, 135.9, 132.1, 130.3 (d, *J* = 32.6 Hz), 129.1 (d, *J* = 3.2 Hz), 128.9, 128.6, 125.8 (q, *J* = 3.5 Hz), 124.1 (d, *J* = 272.1 Hz), 41.9; **^19^F NMR** (375 MHz, CDCl_3_): δ -62.6 (s, 3F); **HRMS** (ESI): calcd for C_24_H_17_NO_2_F_3_S_2_ [M + H]^+^ 472.0653, found 472.0647.

**1-(4-methoxybenzyl)-3,4-bis(phenylthio)-1H-pyrrole-2,5-dione**

Following the general procedure, using (petroleum ether : EtOAc = 9 : 1) as the eluant afforded a yellow liquid (61.5 mg, 71% yield). **^1^H NMR** (500 MHz, CDCl_3_): δ 7.29-7.15 (m, 12H), 6.81-6.80 (m, 2H), 4.57 (s, 2H), 3.75 (s, 3H); **^13^C NMR** (125MHz, CDCl_3_): δ 166.5, 159.3, 135.7, 131.8, 130.2, 128.9, 128.3, 113.9, 55.2, 41.9; **HRMS** (ESI): calcd for C_24_H_19_NO_3_NaS_2_ [M + Na]^+^ 456.0704, found 456.0705.

**1-(4-chlorobenzyl)-3,4-bis(phenylthio)-1H-pyrrole-2,5-dione**

Following the general procedure, using (petroleum ether : EtOAc = 9 : 1) as the eluant afforded a yellow solid (63.8 mg, 73% yield), Mp = 66-67℃. **^1^H NMR** (500 MHz, CDCl_3_): δ 7.28-7.22 (m, 10H), 7.19-7.17 (m, 4H), 4.59 (s, 2H); **^13^C NMR** (125MHz, CDCl_3_): δ 166.4, 135.8, 134.4, 133.9, 131.9, 130.2, 129.0, 128.9, 128.9, 128.5, 41.7; **HRMS** (ESI): calcd for C_23_H_17_NO_2_S_2_Cl [M + H]^+^ 438.0389, found 438.0392.

**1-(4-bromobenzyl)-3,4-bis(phenylthio)-1H-pyrrole-2,5-dione**

Following the general procedure, using (petroleum ether : EtOAc = 9 : 1) as the eluant afforded a yellow liquid (71.0 mg, 74% yield). **^1^H NMR** (500 MHz, CDCl_3_): δ 7.48-7.46 (m, 2H), 7.34-7.23 (m, 12H), 4.63 (s, 2H); **^13^C NMR** (125MHz, CDCl_3_): δ 166.4, 135.8, 134.9, 131.9, 131.8, 130.5, 129.0, 128.9, 128.5, 122.1, 41.8; **HRMS** (ESI): calcd for C_23_H_17_NO_2_S_2_Br [M + H]^+^ 481.9884, found 481.9884.

**1-(naphthalen-1-ylmethyl)-3,4-bis(phenylthio)-1H-pyrrole-2,5-dione**

Following the general procedure, using (petroleum ether : EtOAc = 9 : 1) as the eluant afforded a yellow solid (71.6 mg, 79% yield), Mp = 85-86℃. **^1^H NMR** (500 MHz, CDCl_3_): δ 8.21 (d, *J*=8.30 Hz, 1H), 7.85-7.83 (m, 1H), 7.78 (d, *J*=8.25 Hz, 1H), 7.56 (d, *J*=6.95 Hz, 1H), 7.53-7.46 (m, 2H), 7.42-7.39 (m, 1H), 7.26-7.18 (m, 6H), 7.14-7.13 (m, 3H), 5.12 (s, 2H); **^13^C NMR** (125MHz, CDCl_3_): δ 166.8, 135.9, 133.8, 131.8, 131.2, 131.1, 129.1, 129.0, 128.9, 128.7, 128.3, 128.1, 126.6, 125.9, 125.3, 123.5, 40.5; **HRMS** (ESI): calcd for C_27_H_19_NO_2_NaS_2_ [M + Na]^+^ 476.0755, found 476.0753.

**3,4-bis(phenylthio)-1-(thiophen-2-ylmethyl)-1H-pyrrole-2,5-dione**

Following the general procedure, using (petroleum ether : EtOAc = 9 : 1) as the eluant afforded a yellow liquid (62.9 mg, 77% yield). **^1^H NMR** (500 MHz, CDCl_3_): δ 7.34-7.24 (m, 11H), 7.12-7.10 (m, 1H), 6.98-6.96 (m, 1H), 4.86 (s, 2H); **^13^C NMR** (125MHz, CDCl_3_): δ 166.1, 137.7, 135.9, 131.9, 129.0, 129.0, 128.5, 127.9, 127.0, 126.1, 36.5; **HRMS** (ESI): calcd for C_21_H_15_NO_2_NaS_3_ [M + Na]^+^ 432.0163, found 432.0167.

**1-(3,4-dichlorobenzyl)-3,4-bis(phenylthio)-1H-pyrrole-2,5-dione**

Following the general procedure, using (petroleum ether : EtOAc = 9 : 1) as the eluant afforded a yellow solid (56.5 mg, 60% yield), Mp = 86-87℃. **^1^H NMR** (400 MHz, CDCl_3_): δ 7.47 (d, *J* = 2.1 Hz, 1H), 7.40 (d, *J* = 8.2 Hz, 1H), 7.36-7.21 (m, 11H), 4.62 (s, 2H); **^13^C NMR** (100MHz, CDCl_3_): δ 166.3, 136.0, 135.9, 132.8, 132.3, 132.1, 130.8, 129.1, 128.9, 128.6, 128.2, 41.3; **HRMS** (ESI): calcd for C_23_H_16_NO_2_S_2_Cl_2_ [M + H]^+^ 471.9999, found 472.0003.

**1,1'-(methylenebis(4,1-phenylene))bis(3,4-bis(phenylthio)-1H-pyrrole-2,5-dione)**

Following the general procedure, using (petroleum ether : EtOAc = 9 : 1) as the eluant afforded a yellow solid (121.6 mg, 77% yield), Mp = 154-155℃. **^1^H NMR** (400 MHz, CDCl_3_): δ 7.34-7.23 (m, 28H), 4.01 (s, 2H); **^13^C NMR** (100MHz, CDCl_3_): δ 165.7, 140.3, 135.8, 132.2, 129.7, 129.1, 128.8, 128.6, 126.0, 41.1; **HRMS** (ESI): calcd for C_45_H_31_N_2_O_4_S_4_ [M + H]^+^ 791.1166, found 791.1181.

**1,1'-(1,3-phenylene)bis(3,4-bis(phenylthio)-1H-pyrrole-2,5-dione)**

Following the general procedure, using (petroleum ether : EtOAc = 9 : 1) as the eluant afforded a yellow liquid (100.8 mg, 72% yield). **^1^H NMR** (400 MHz, CDCl_3_): δ 7.55-7.50 (m, 4H), 7.44-7.30 (m, 17H), 6.87-6.85 (m, 3H); **^13^C NMR** (100MHz, CDCl_3_): δ 169.15, 165.26, 136.00, 134.31, 132.33, 132.19, 131.88, 129.48, 129.18, 128.74, 124.79, 124.66, 122.57; **HRMS** (ESI): calcd for C_38_H_25_N_2_O_4_S_4_ [M + H]^+^ 701.0697, found 701.0691.

**1,1'-(((propane-2,2-diylbis(4,1-phenylene))bis(oxy))bis(4,1-phenylene))bis(3,4-bis(phenylthio)-1H-pyrrole-2,5-dione)**

Following the general procedure, using (petroleum ether : EtOAc = 9 : 1) as the eluant afforded a yellow solid (120.2 mg, 60% yield), Mp = 103-104℃. **^1^H NMR** (400 MHz, CDCl_3_): δ 7.35-7.29 (m, 24H), 7.25 (d, *J* = 8.7 Hz, 4H), 7.07 (d, *J* = 8.9 Hz, 4H), 6.97 (d, *J* = 8.7 Hz, 4H), 1.72 (s, 6H); **^13^C NMR** (100MHz, CDCl_3_): δ 165.8, 157.1, 154.4, 146.1, 135.8, 132.3, 129.1, 128.8, 128.7, 128.2, 127.5, 126.1, 118.8, 42.3, 31.1, 29.7; **HRMS** (ESI): calcd for C_59_H_42_N_2_O_6_NaS_4_ [M + Na]^+^ 1025.1823, found 1025.1830.

**References:**

(1) (a) Ding, G.; Li, C.; Shen, Y.; Lu, B.; Zhang, Z.; Xie, X. *Adv. Synth. Catal.* **2016**, *358*, 1241-1250. (b) Matuszak, N.; Muccioli, G. G.; Labar, G.; Lambert, D. M. *J. Med Chem*. **2009**, *52*, 7410-7420.

**^1^H, ^13^C and ^19^F NMR spectra of product**

**
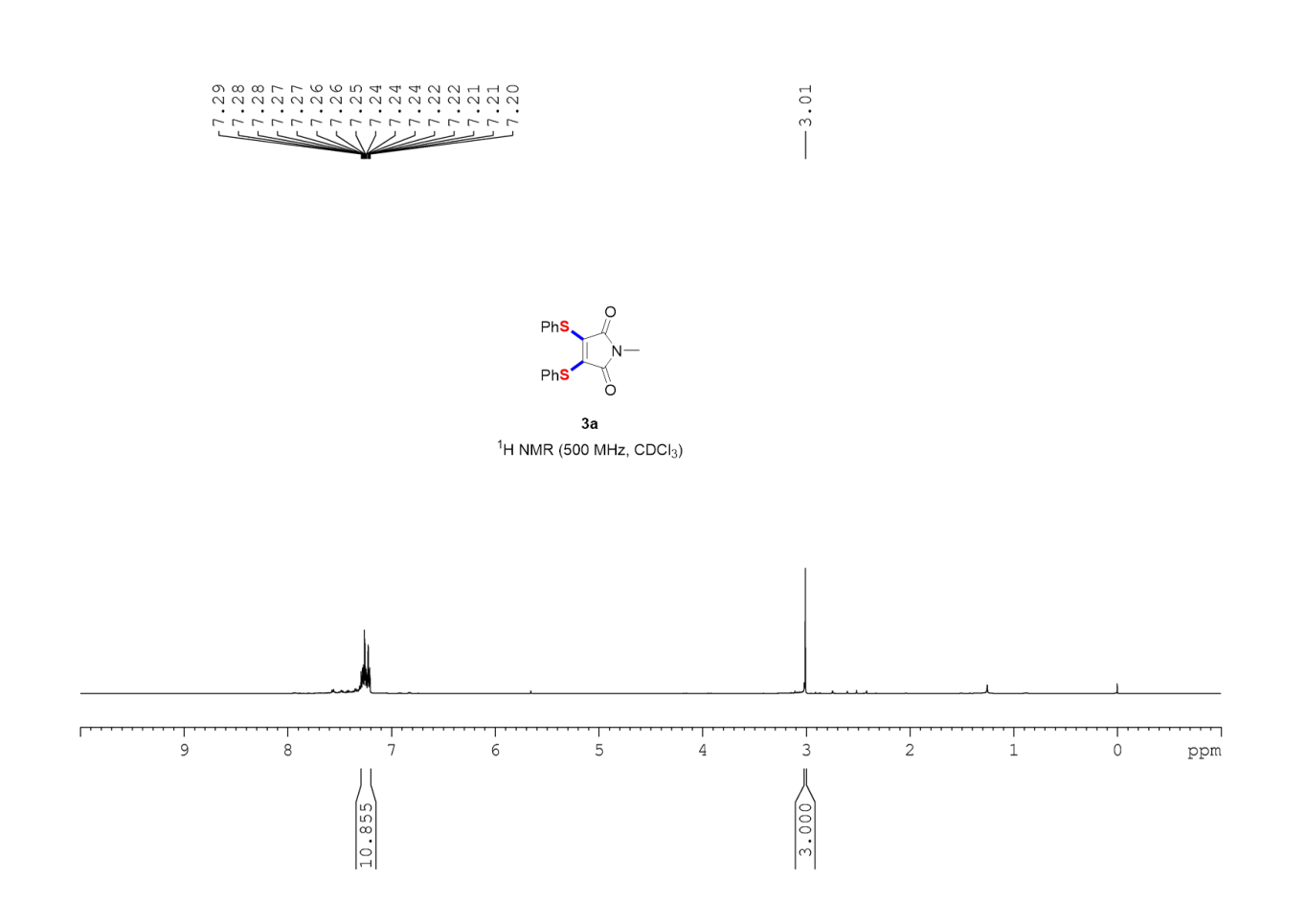
**

**
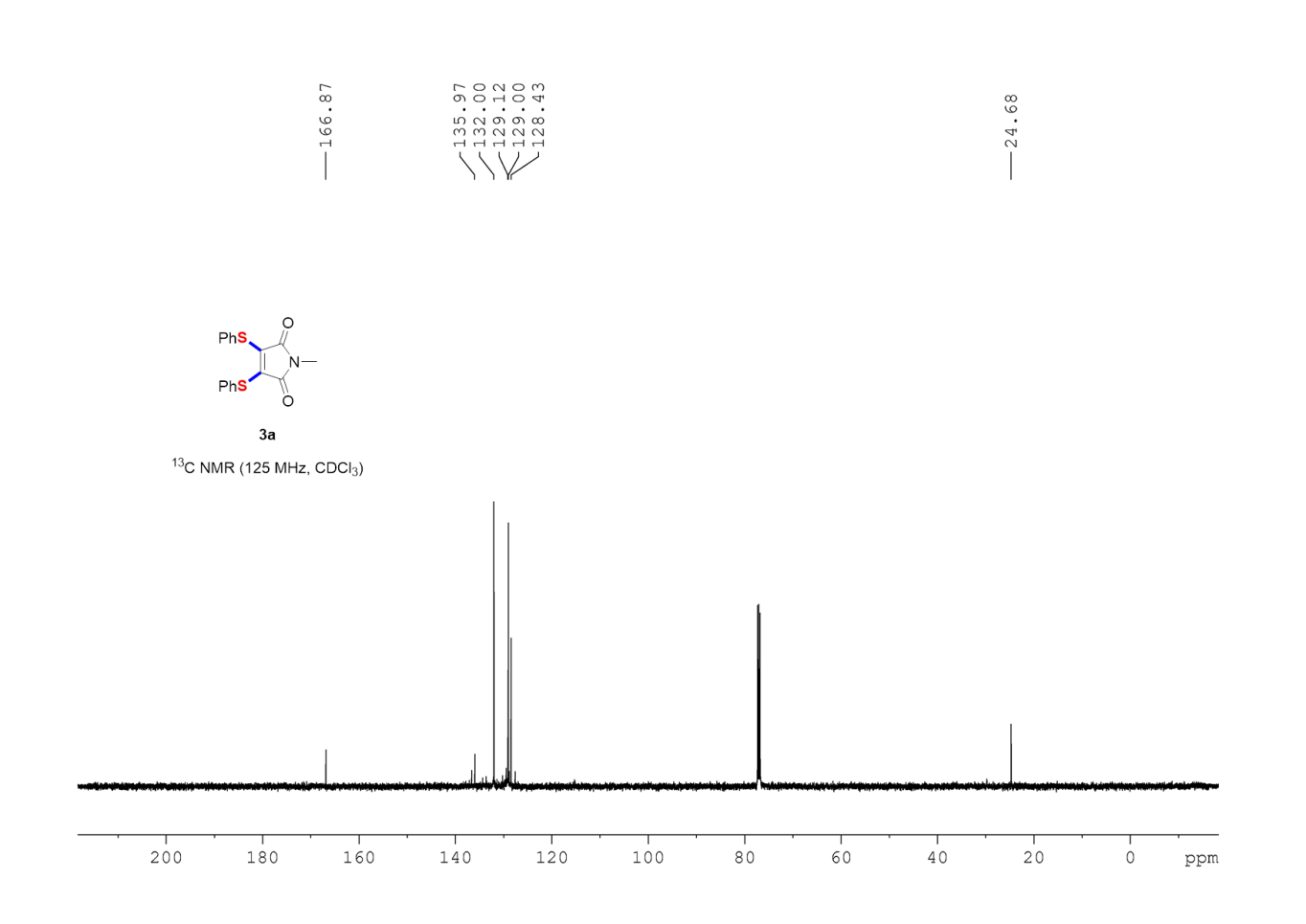
**

**
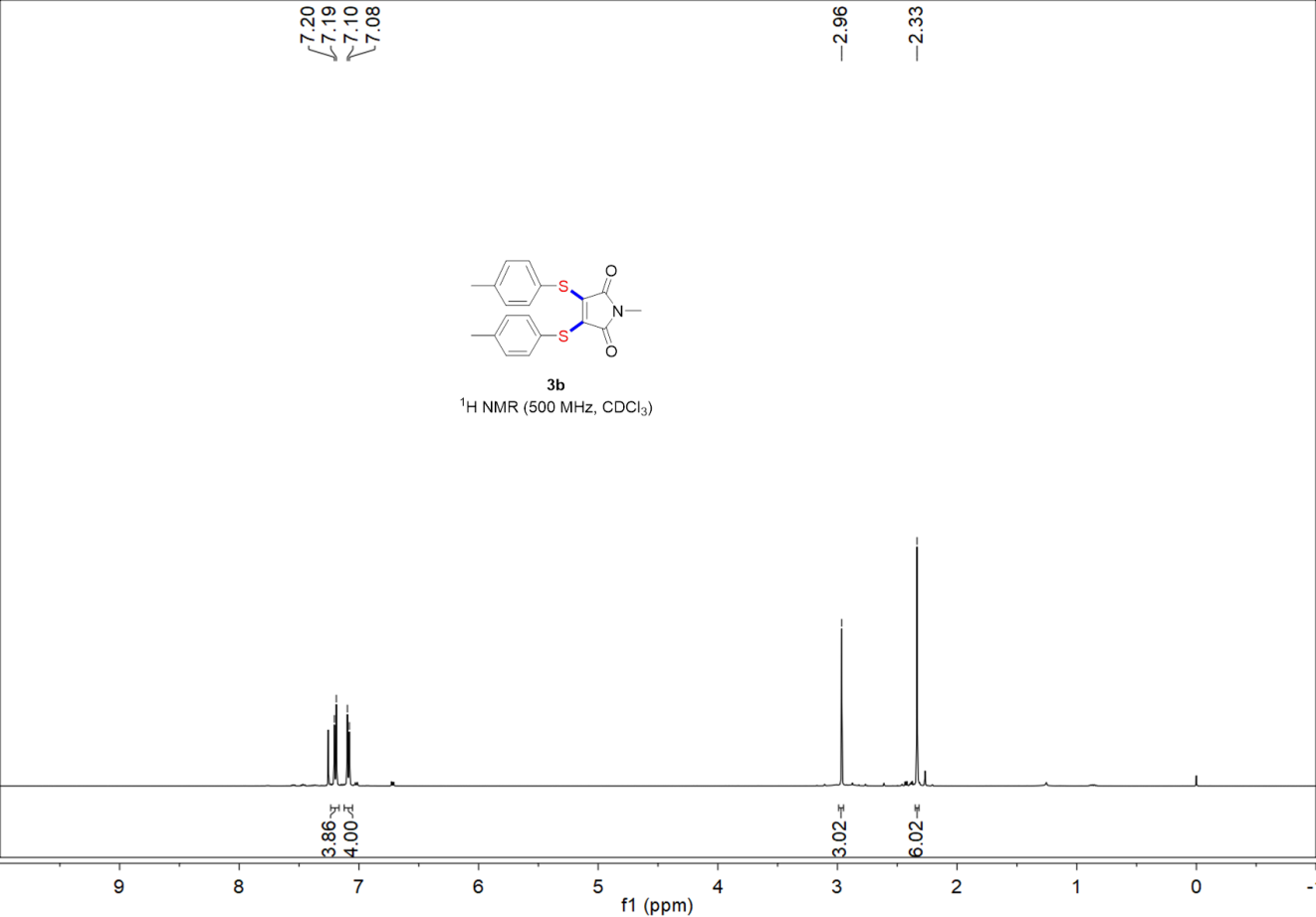
**

**
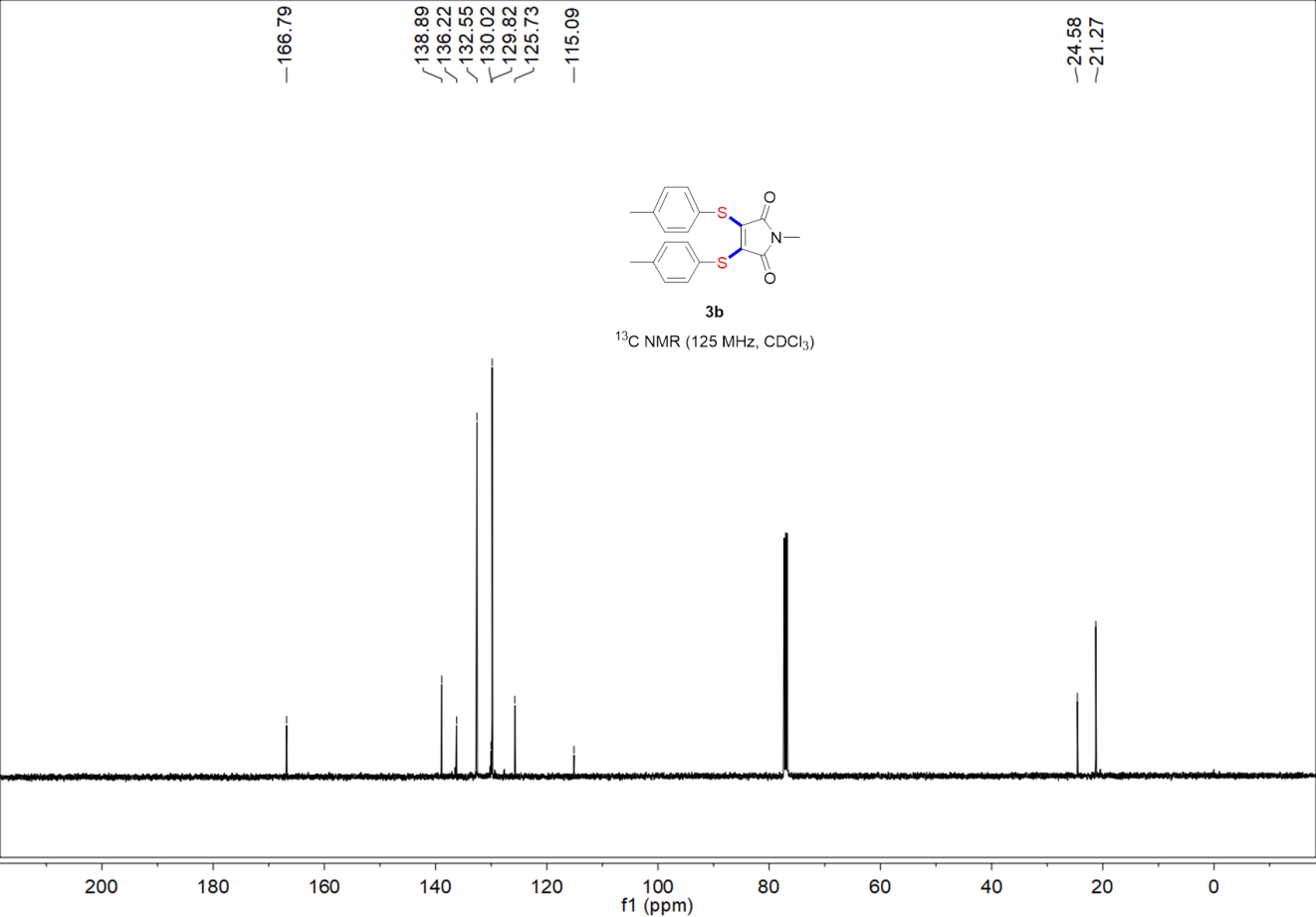
**

**
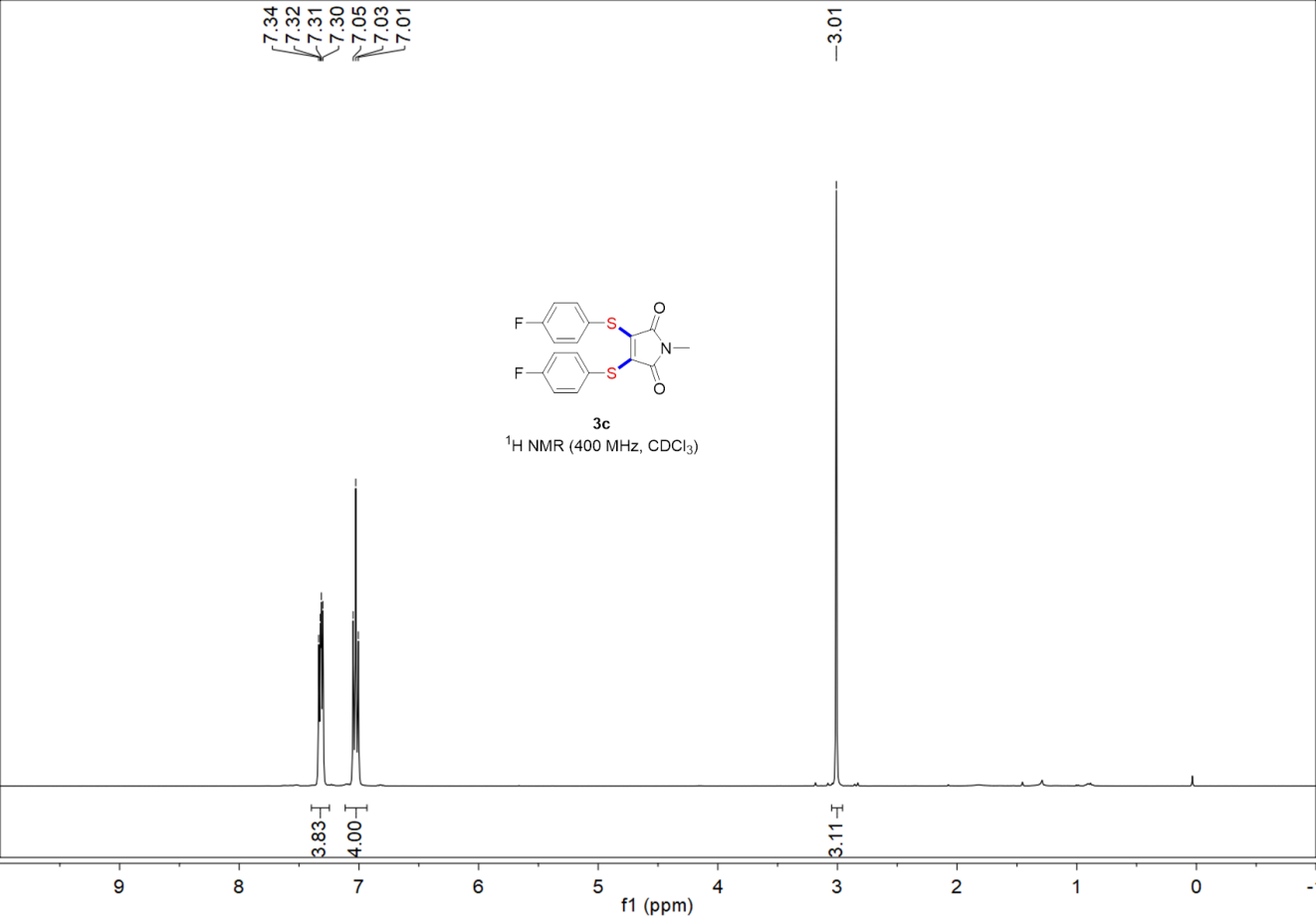
**

**
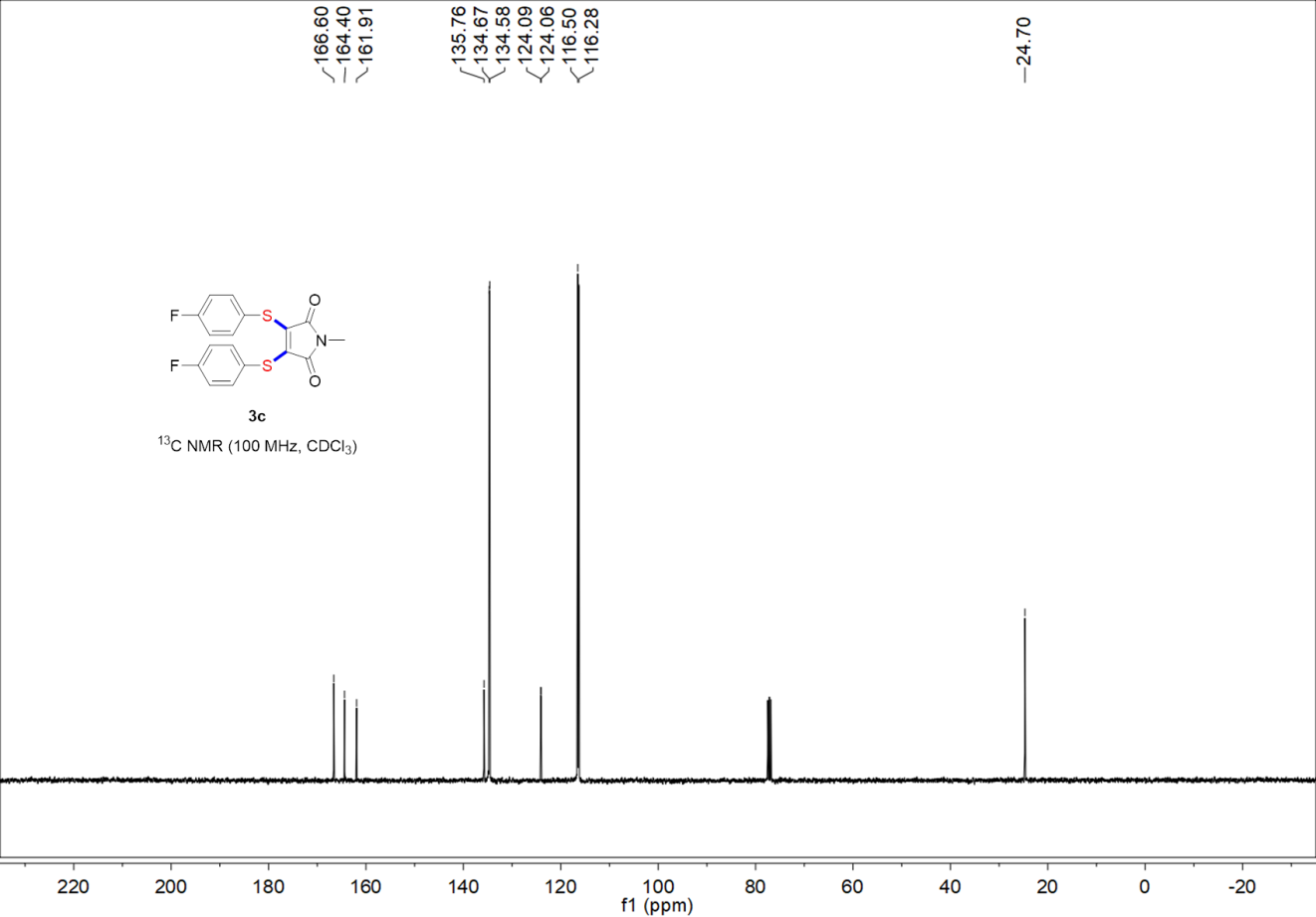
**

**
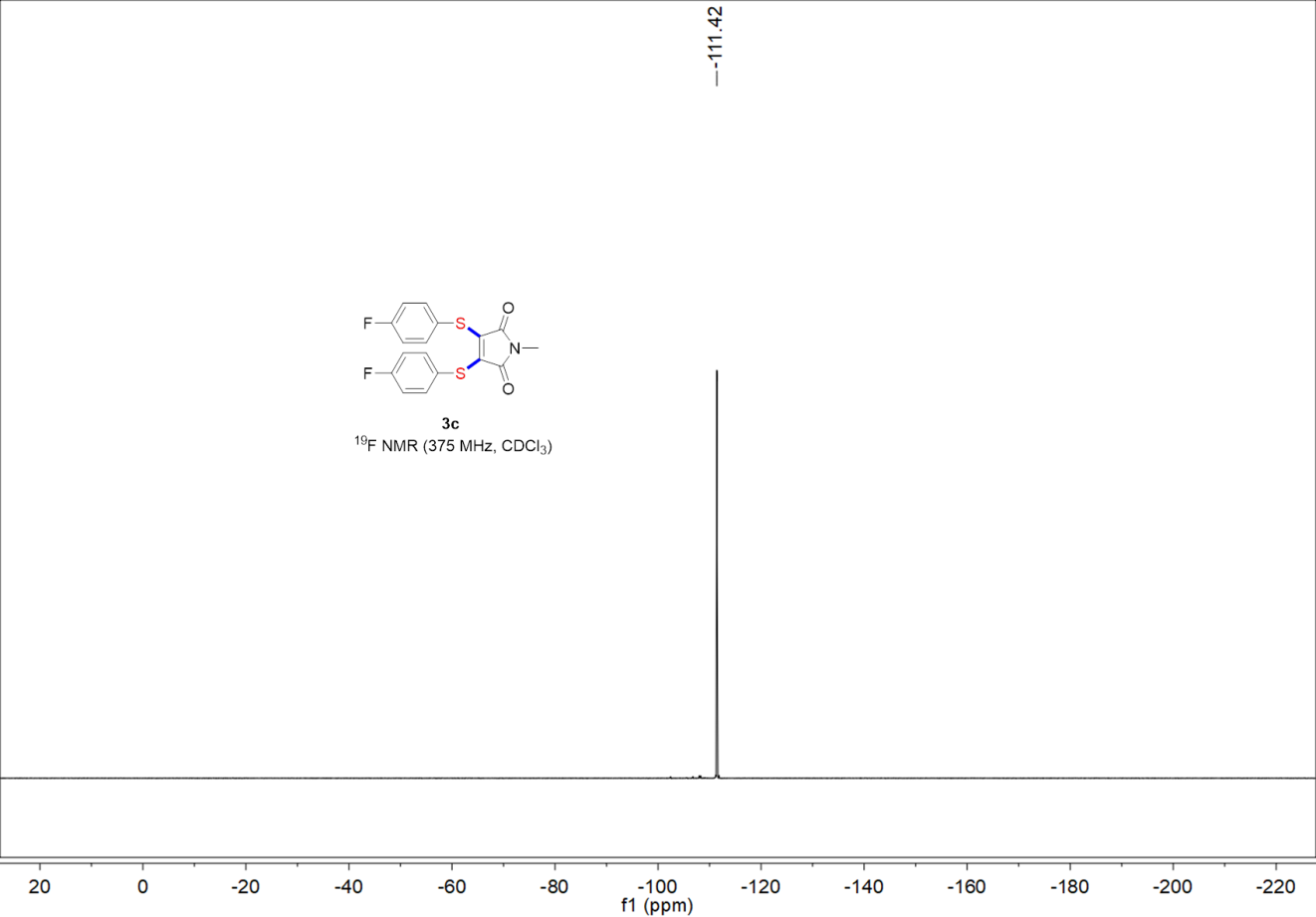
**

**
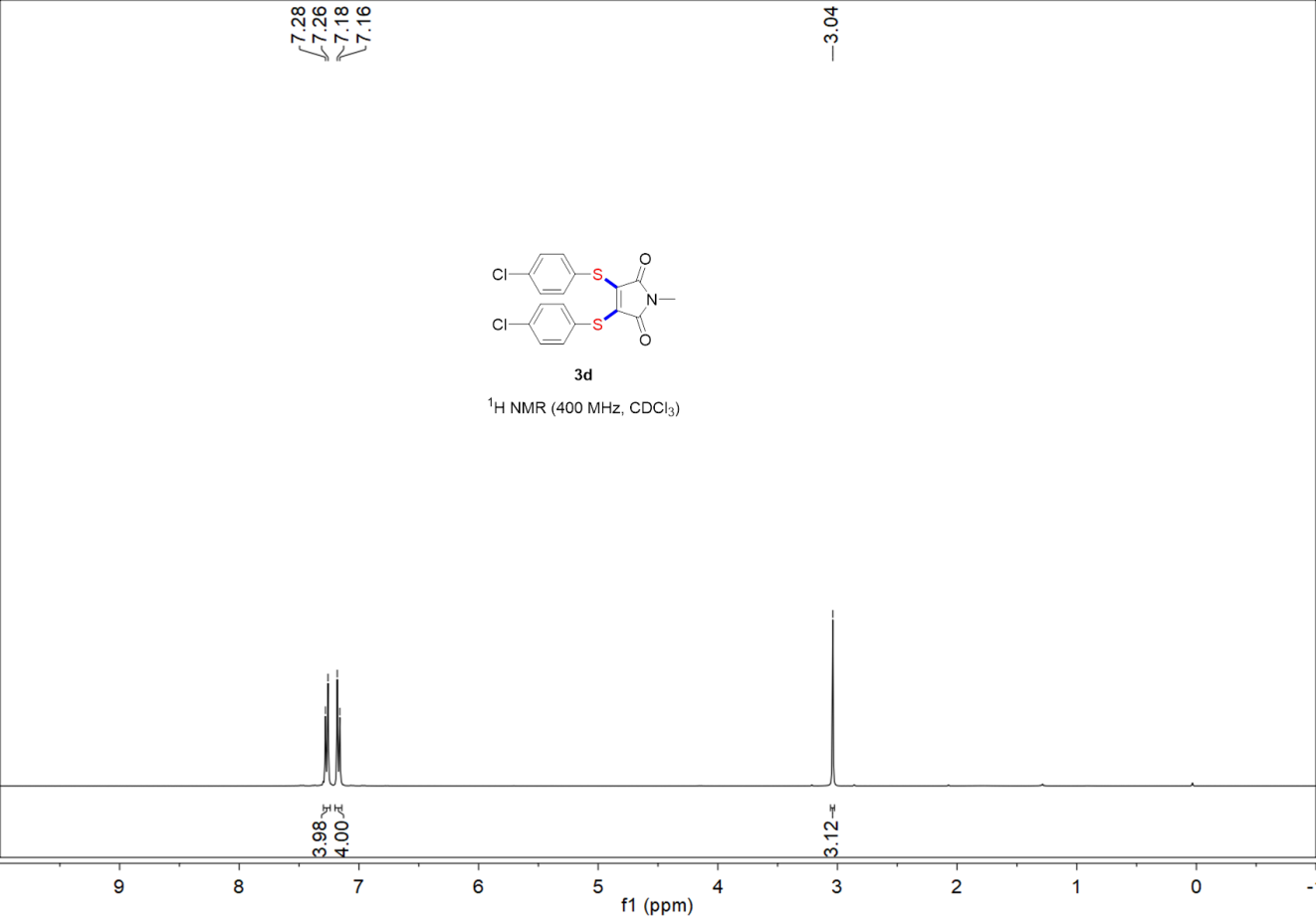
**

**
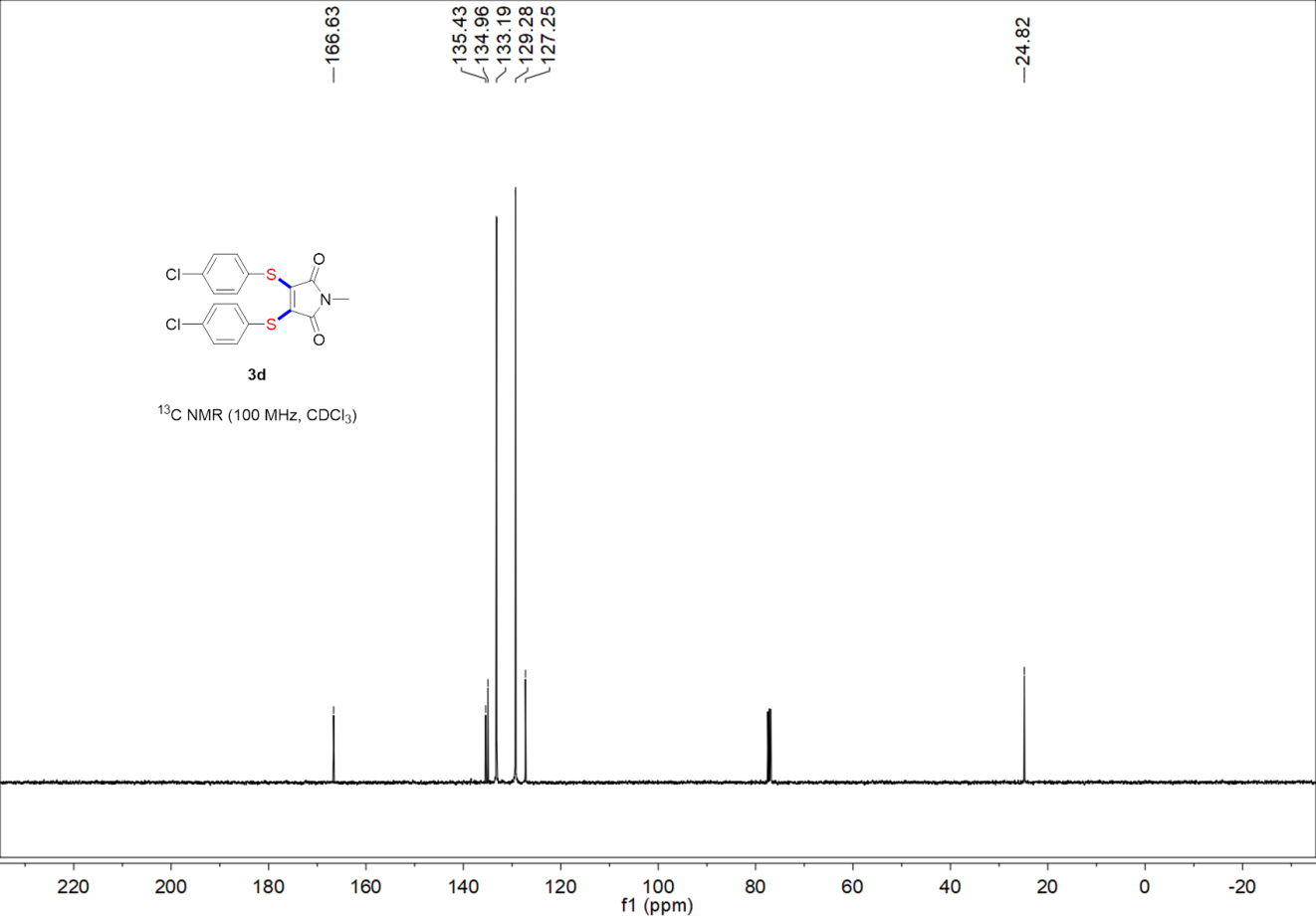
**

**
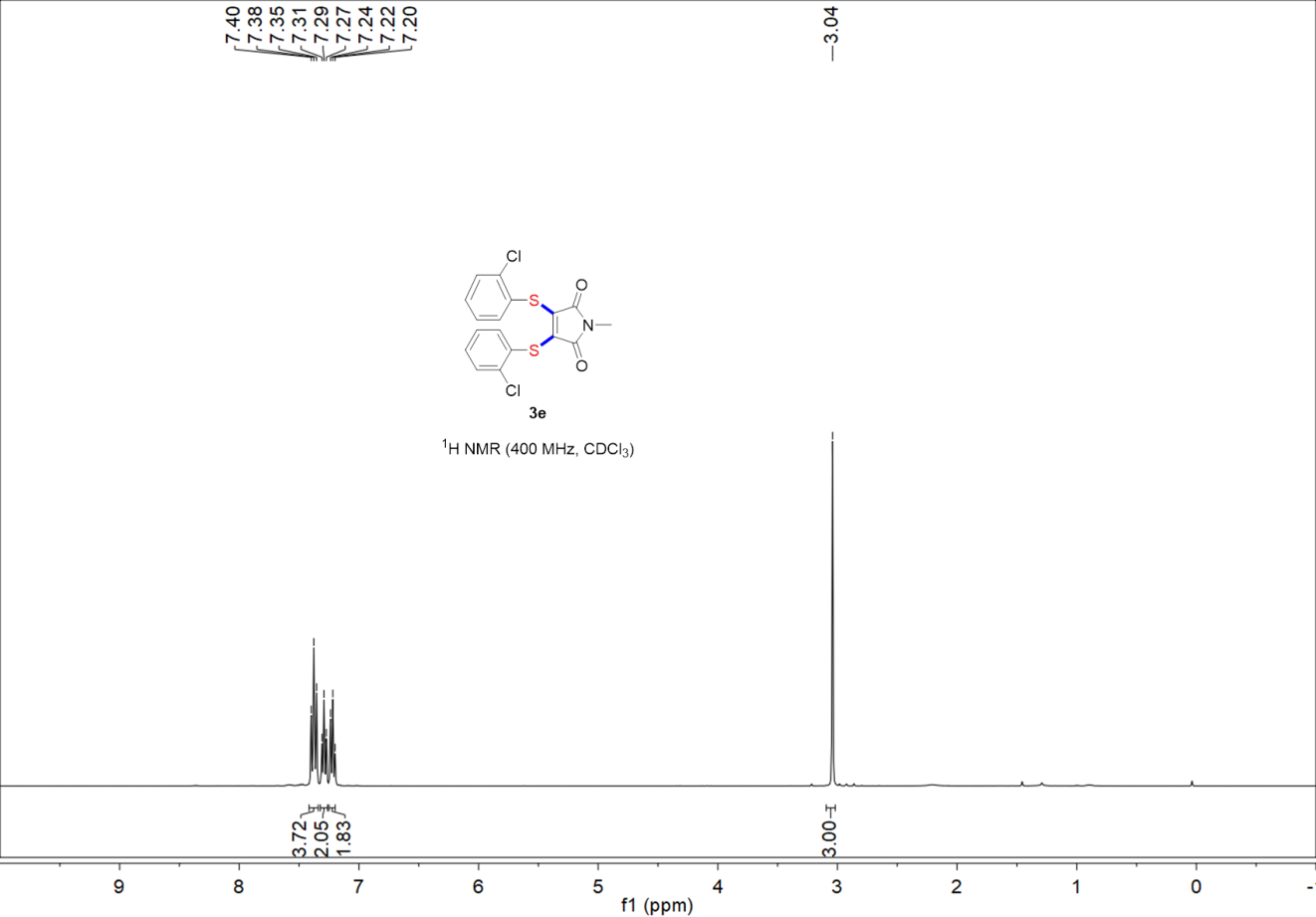
**

**
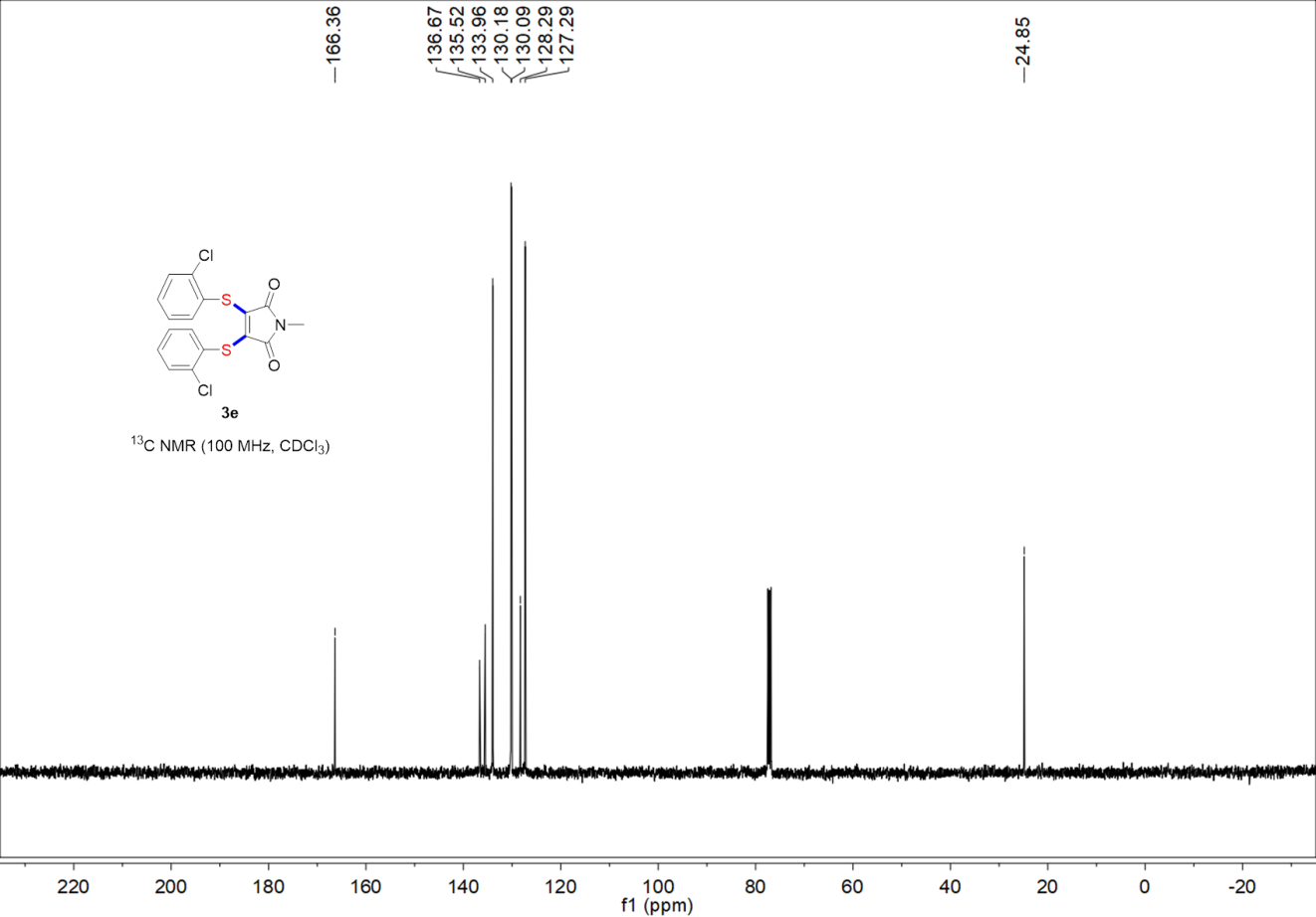
**

**
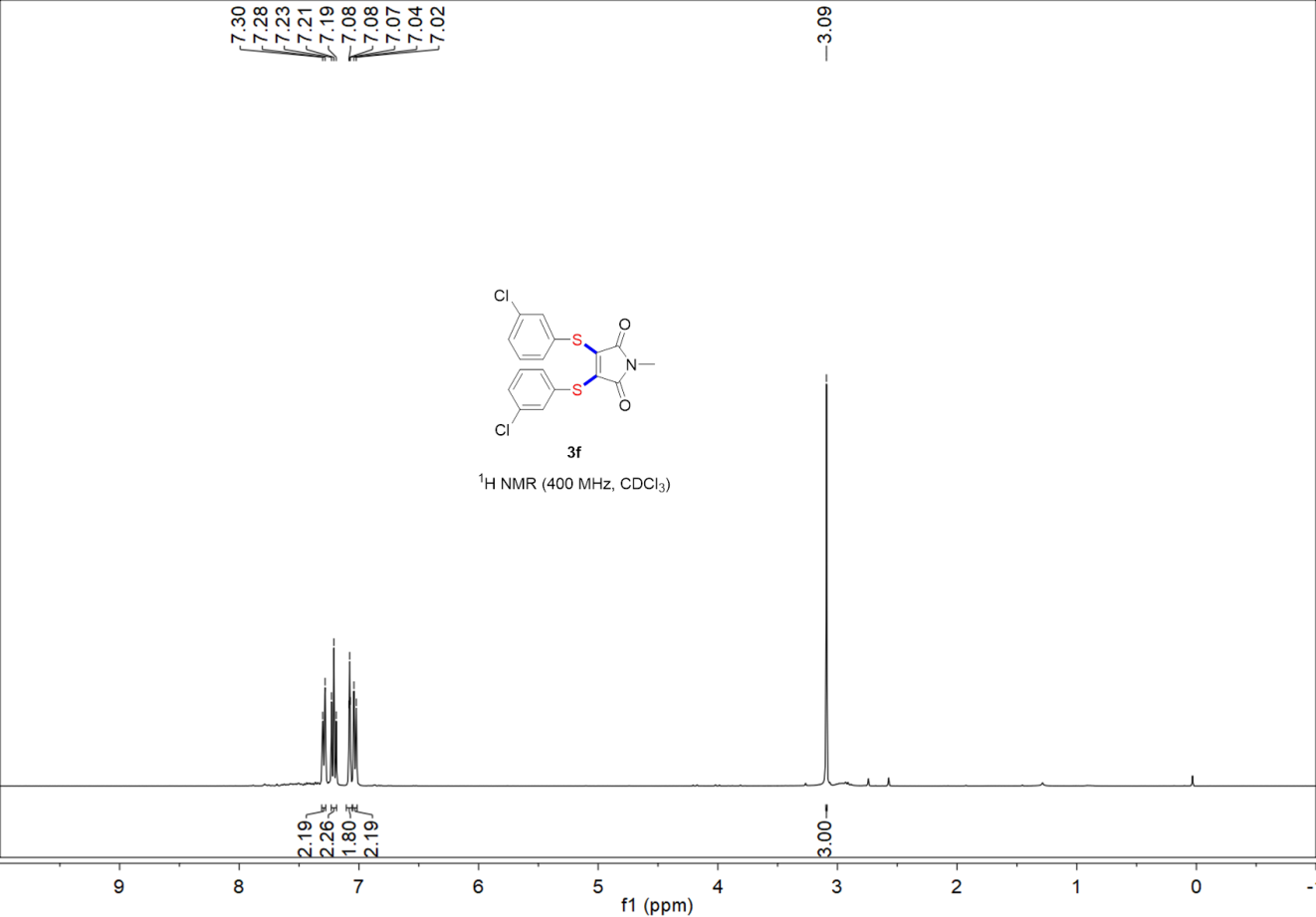
**

**
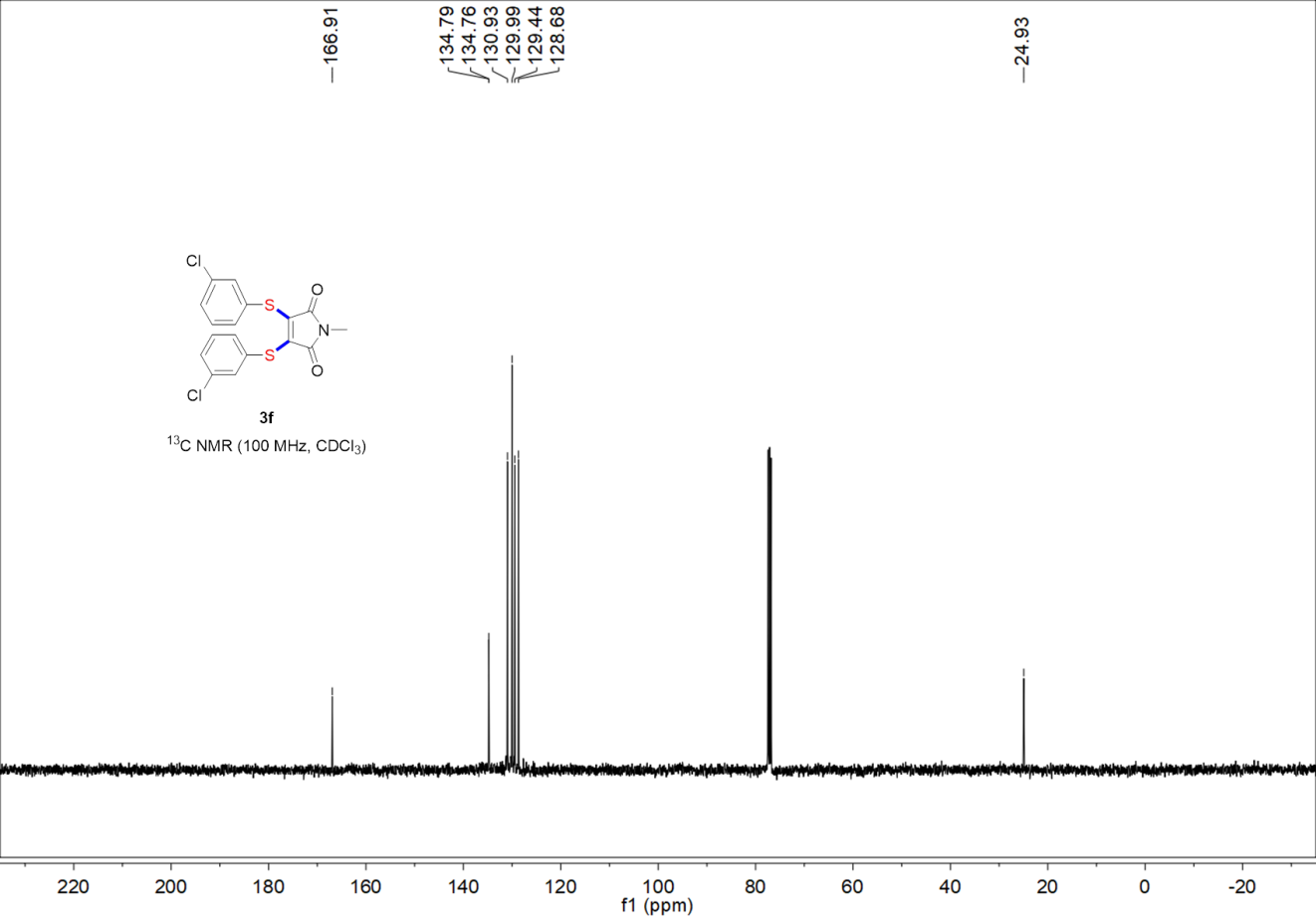
**

**
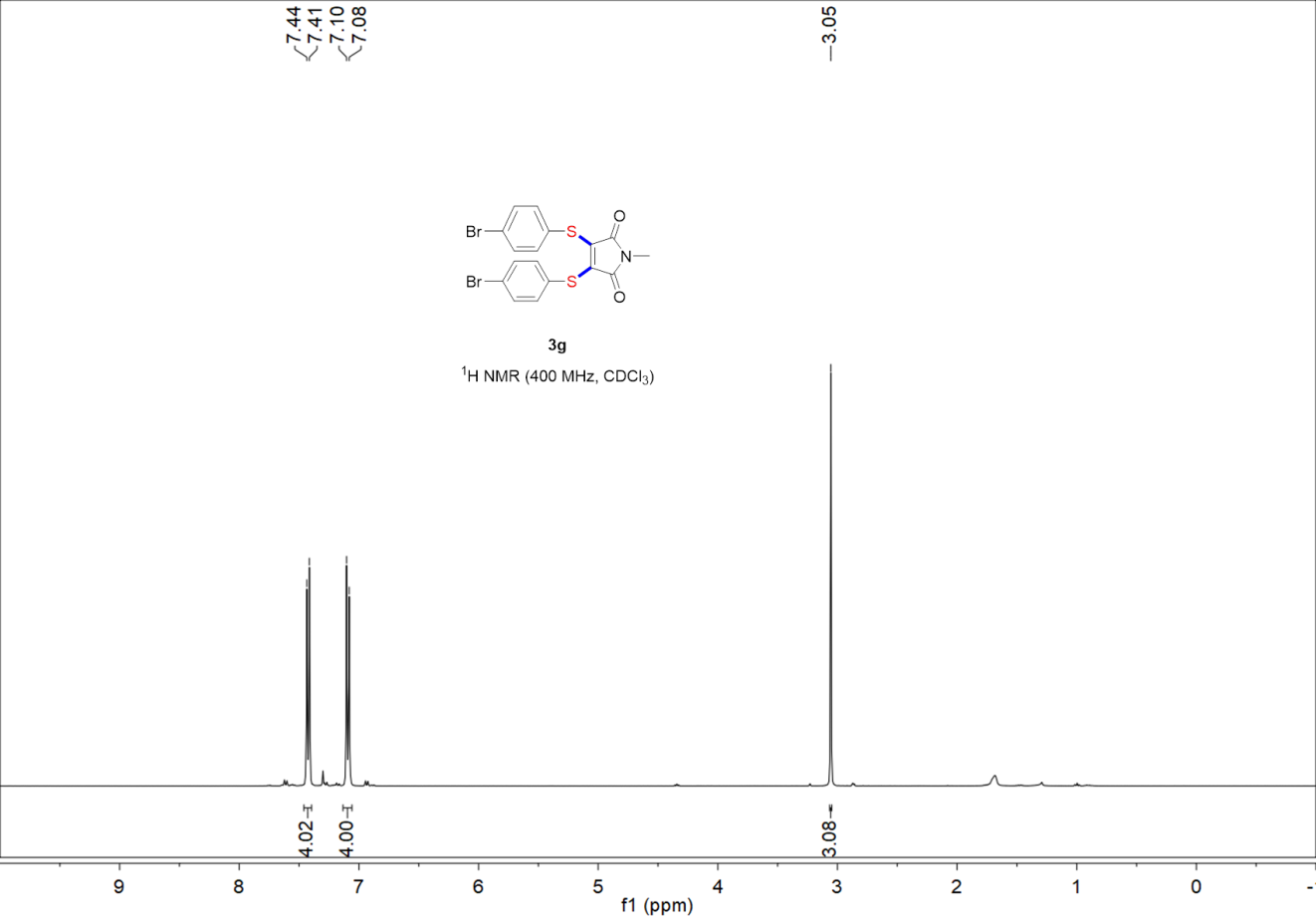
**

**
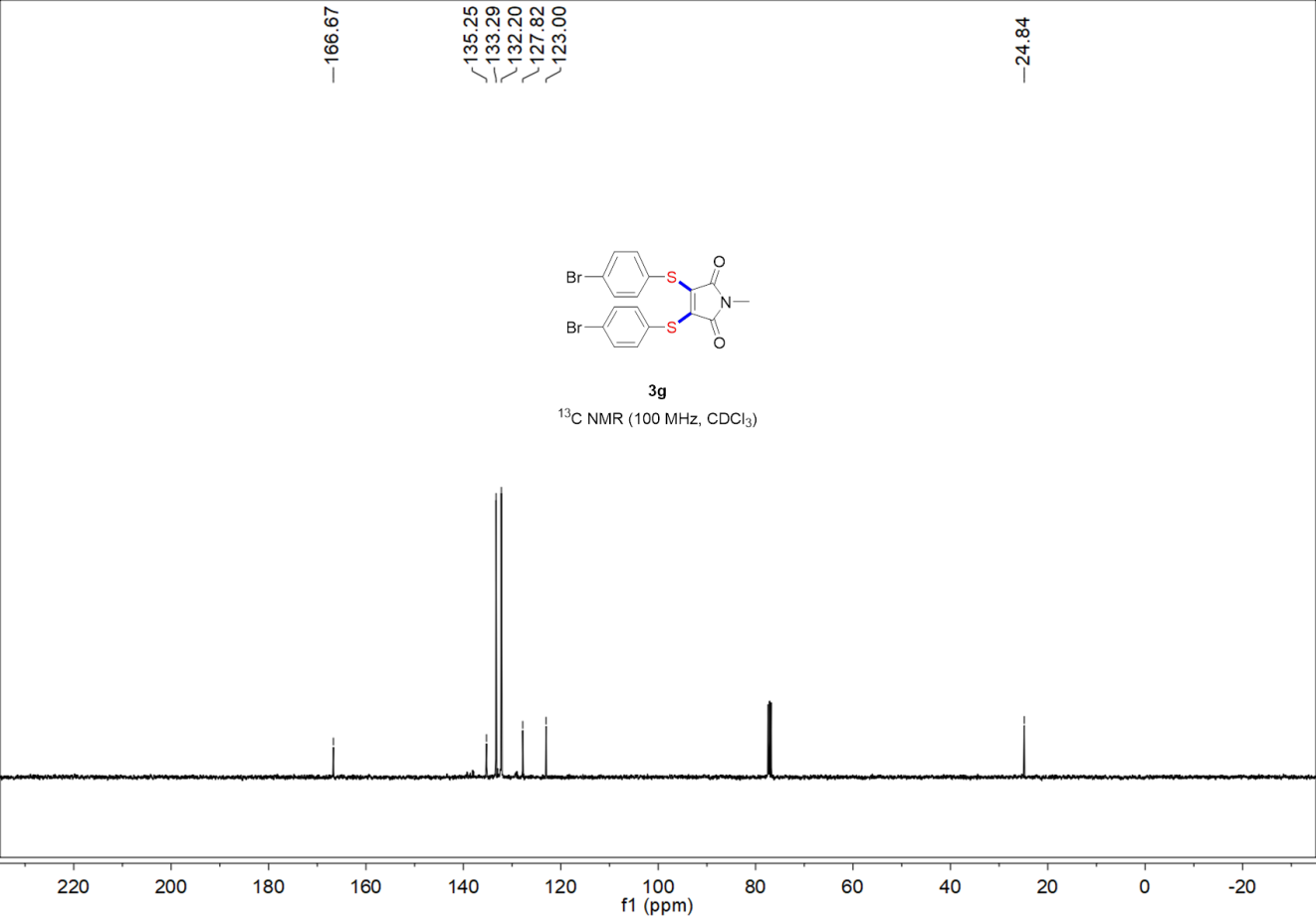
**

**
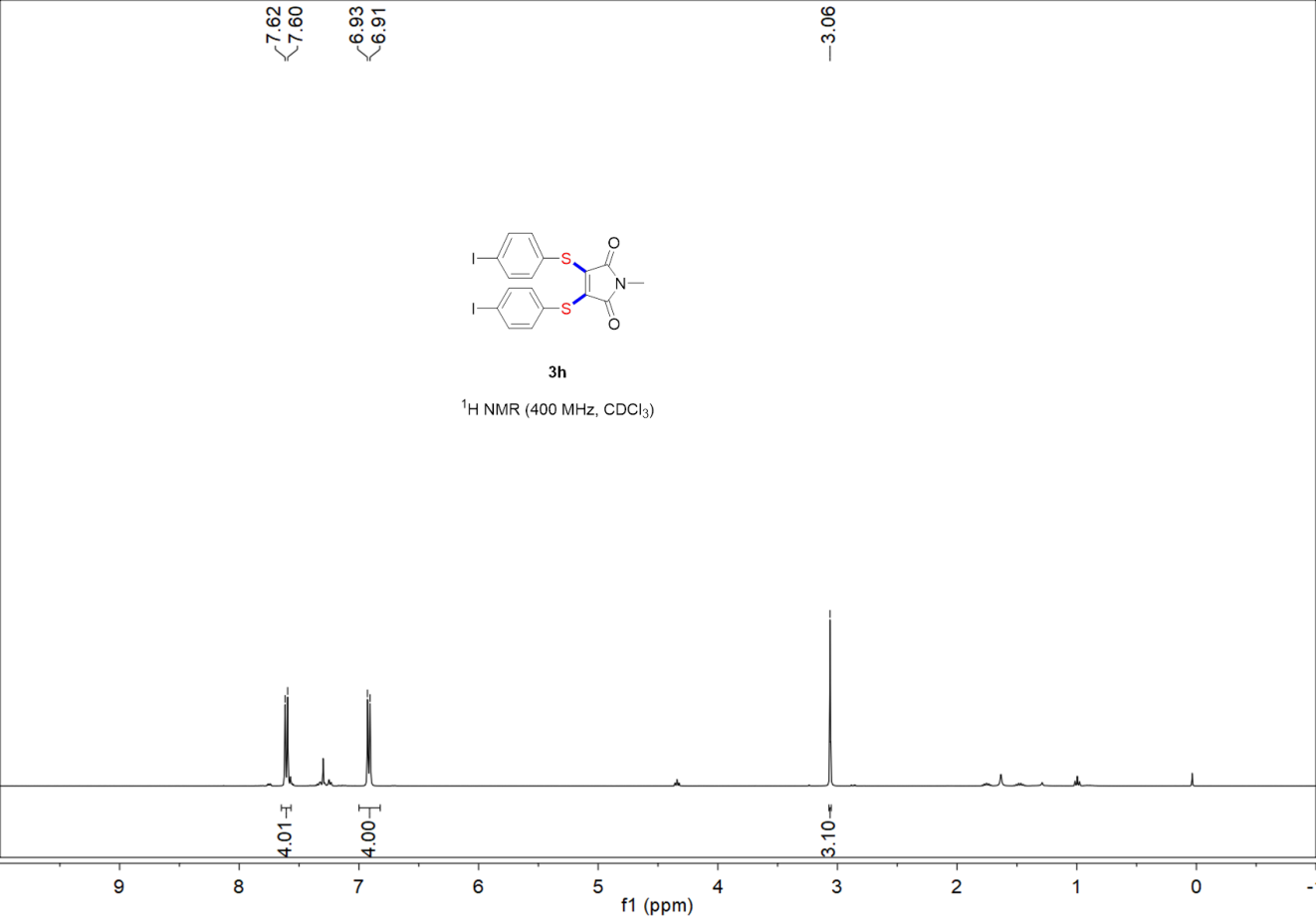
**

**
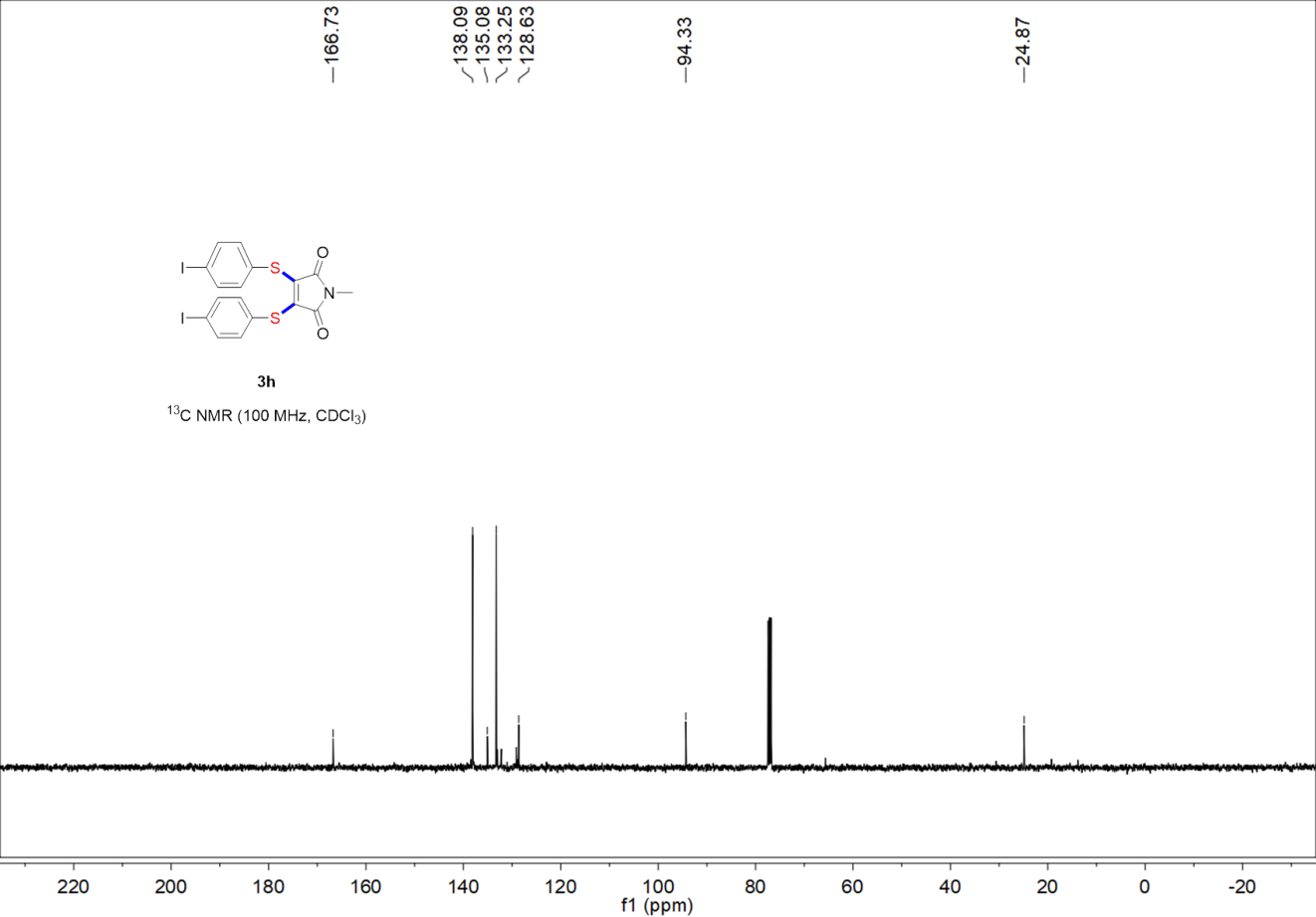
**

**
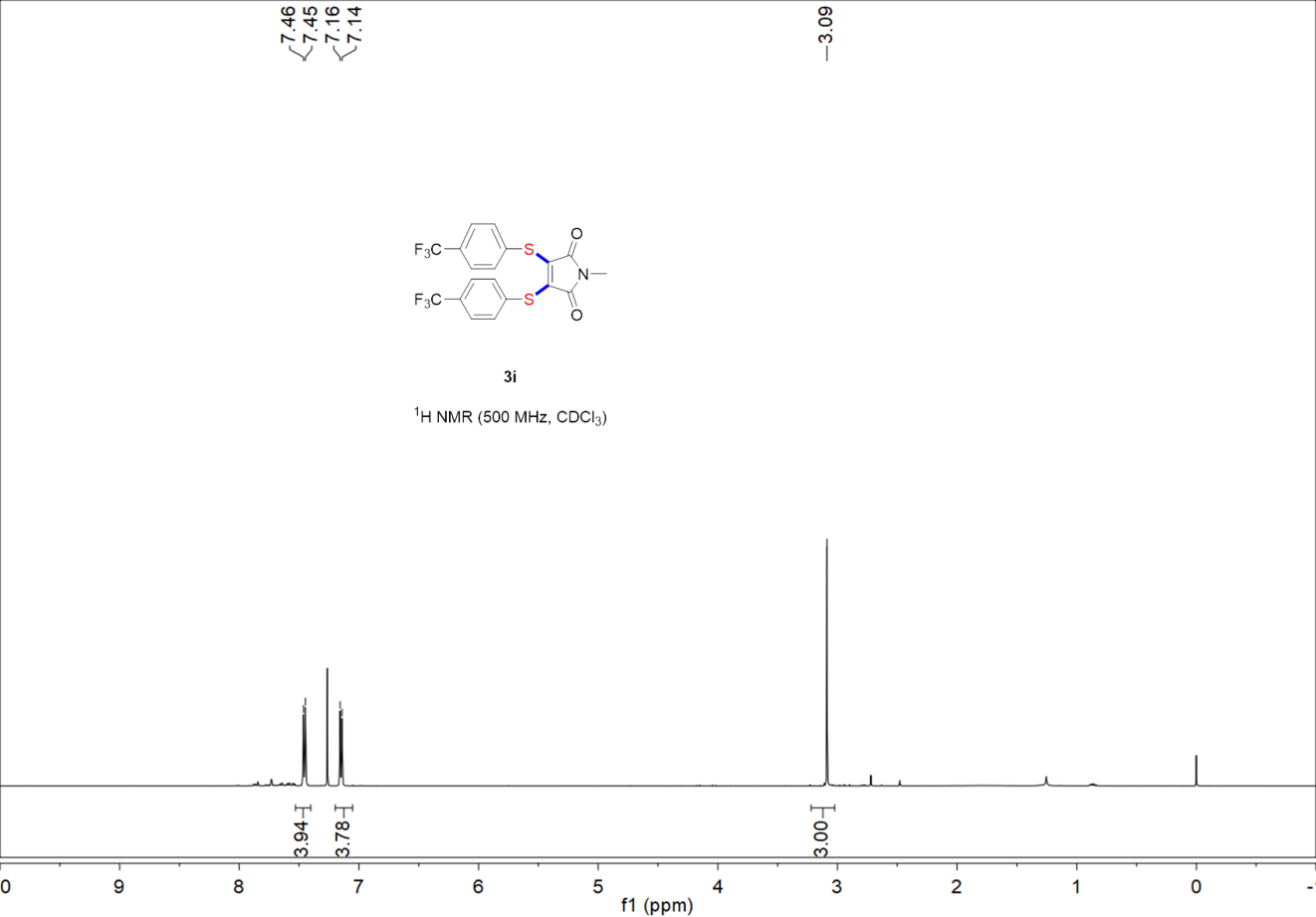
**

**
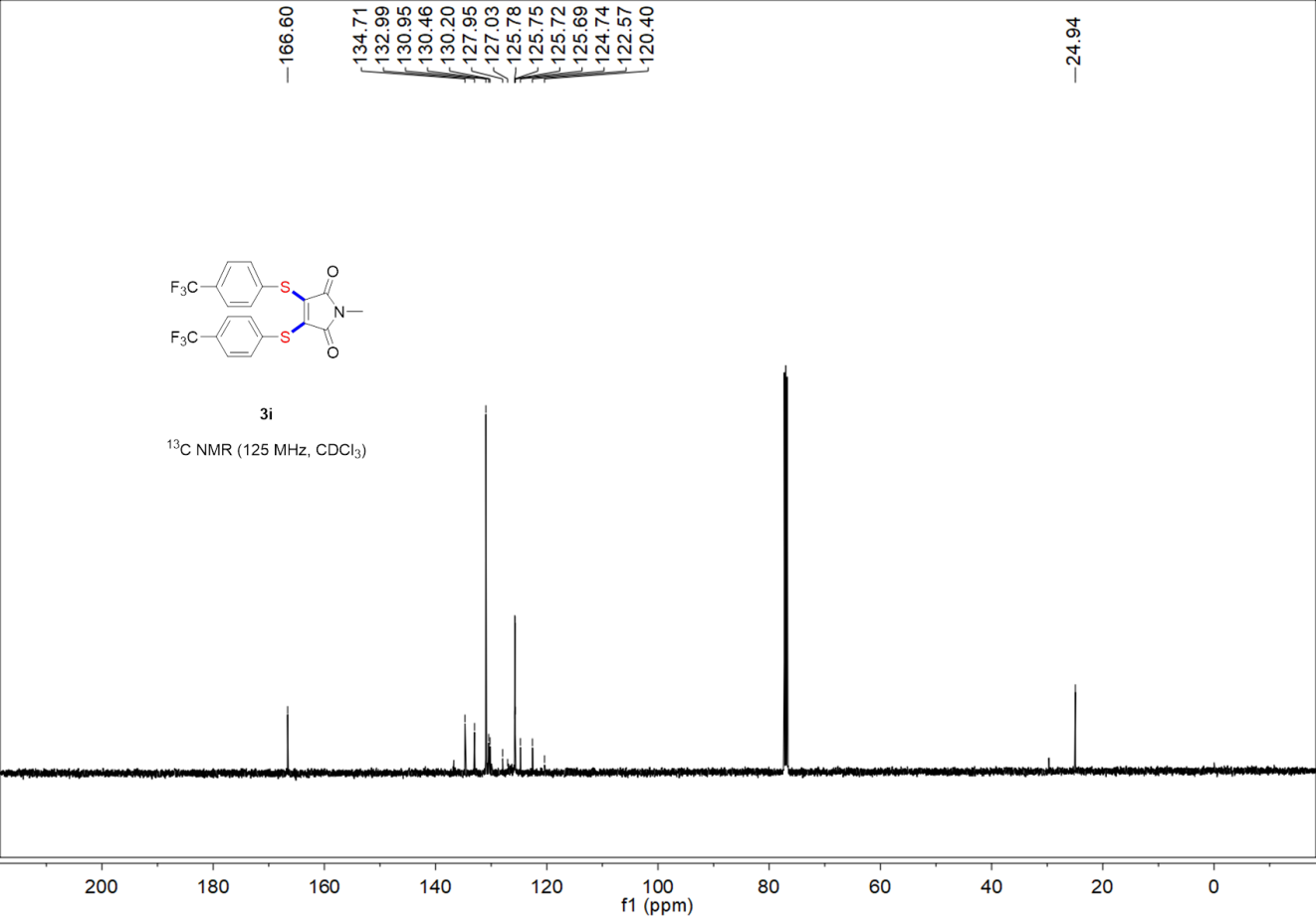
**

**
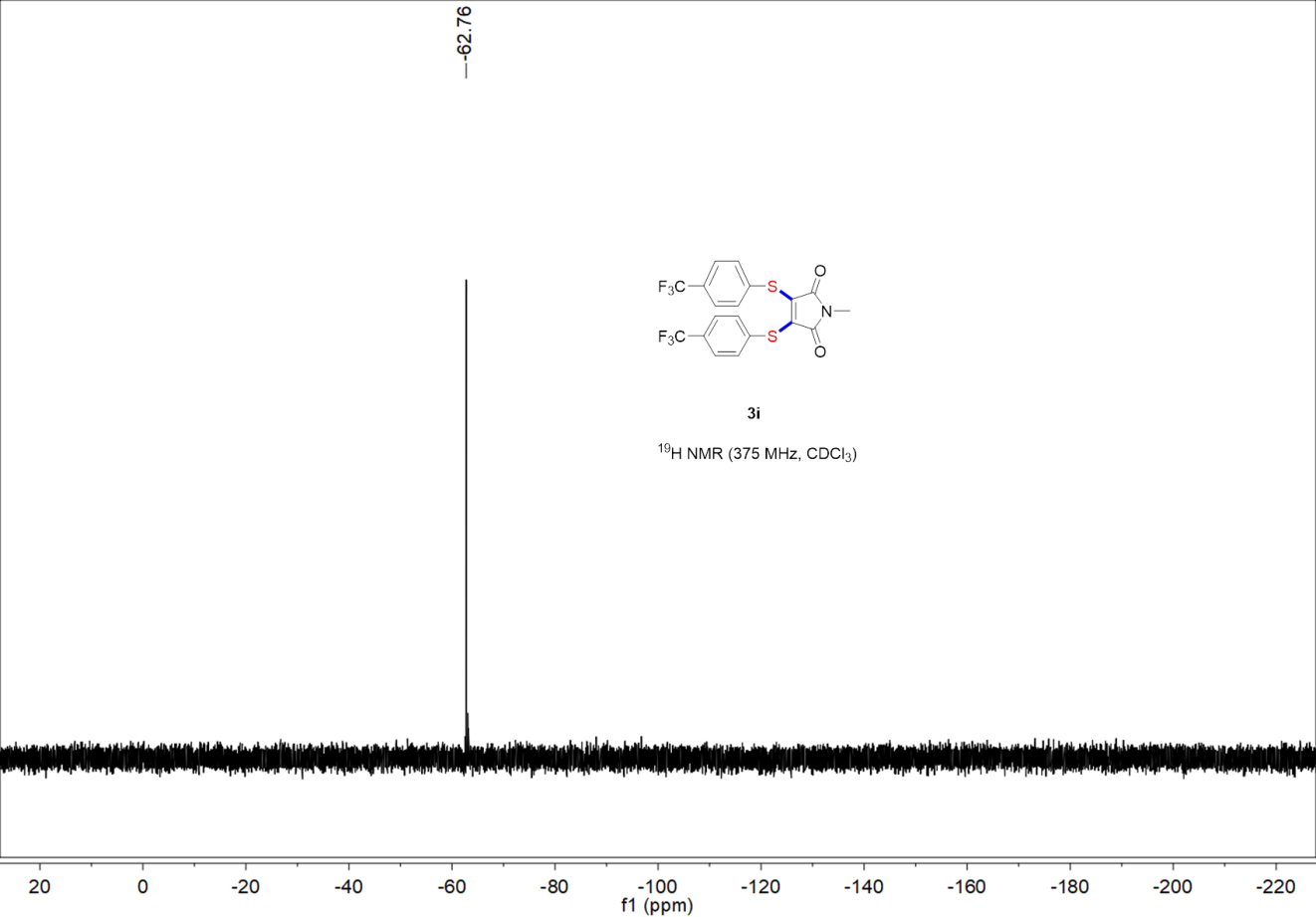
**

**
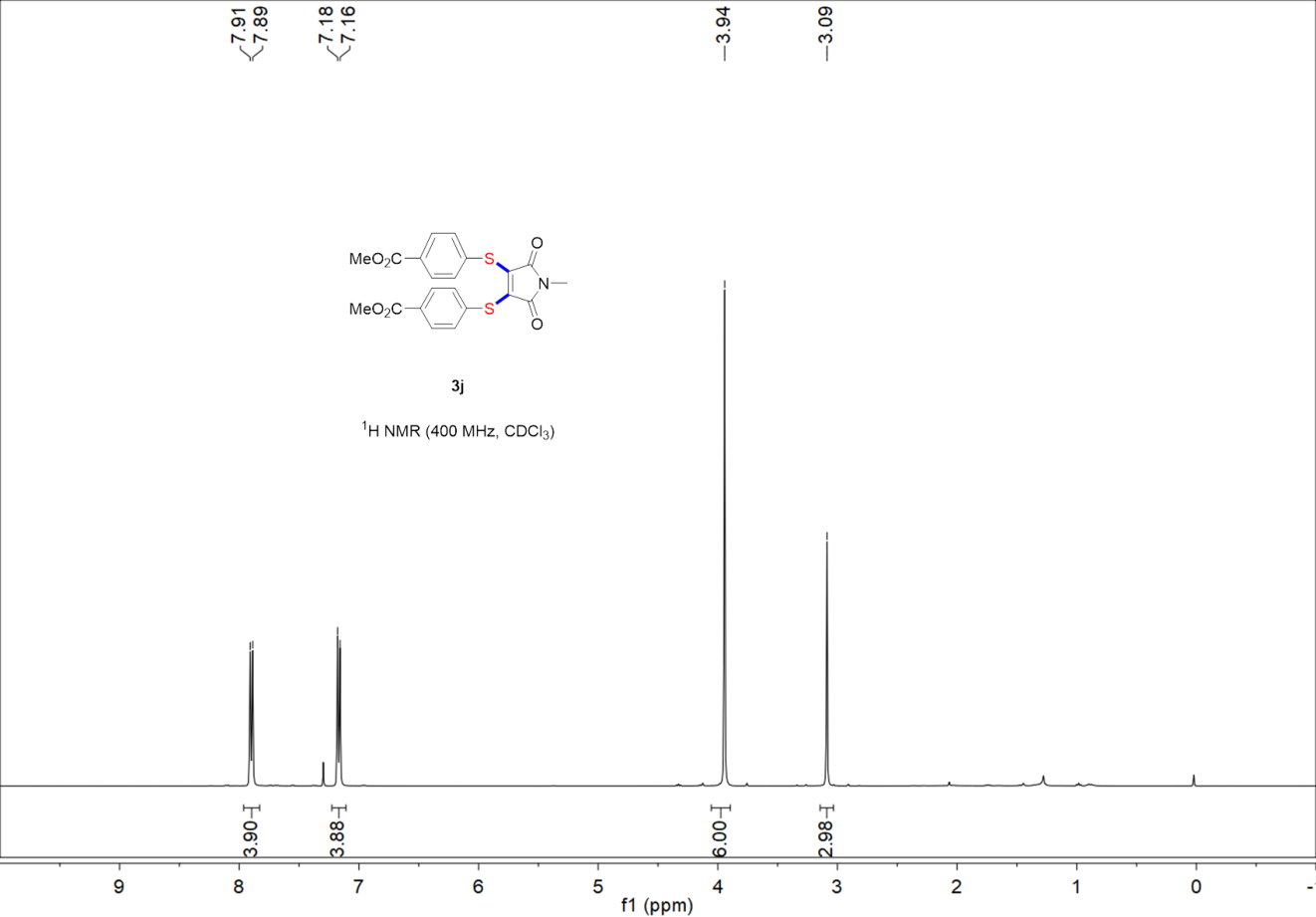
**

**
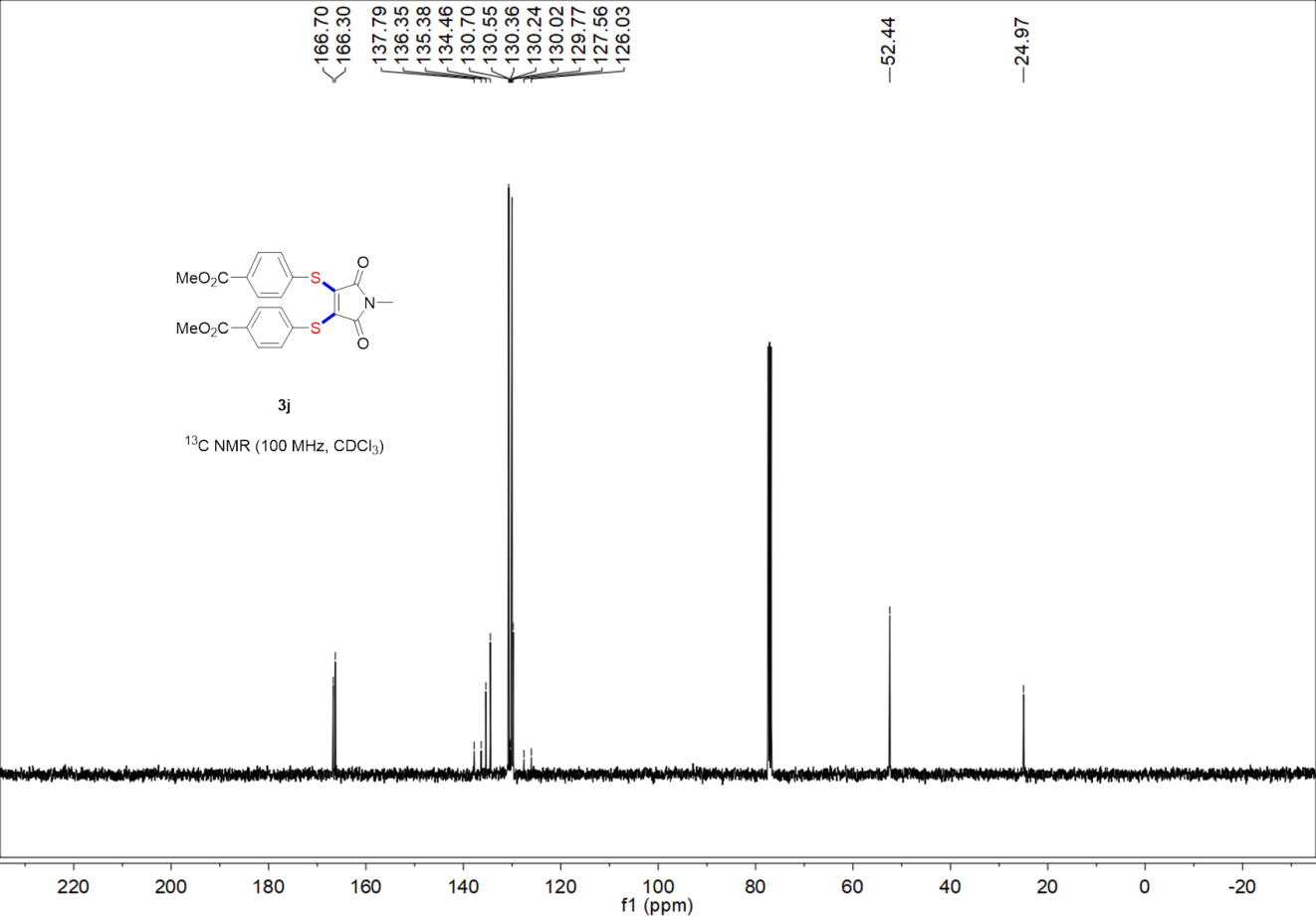
**

**
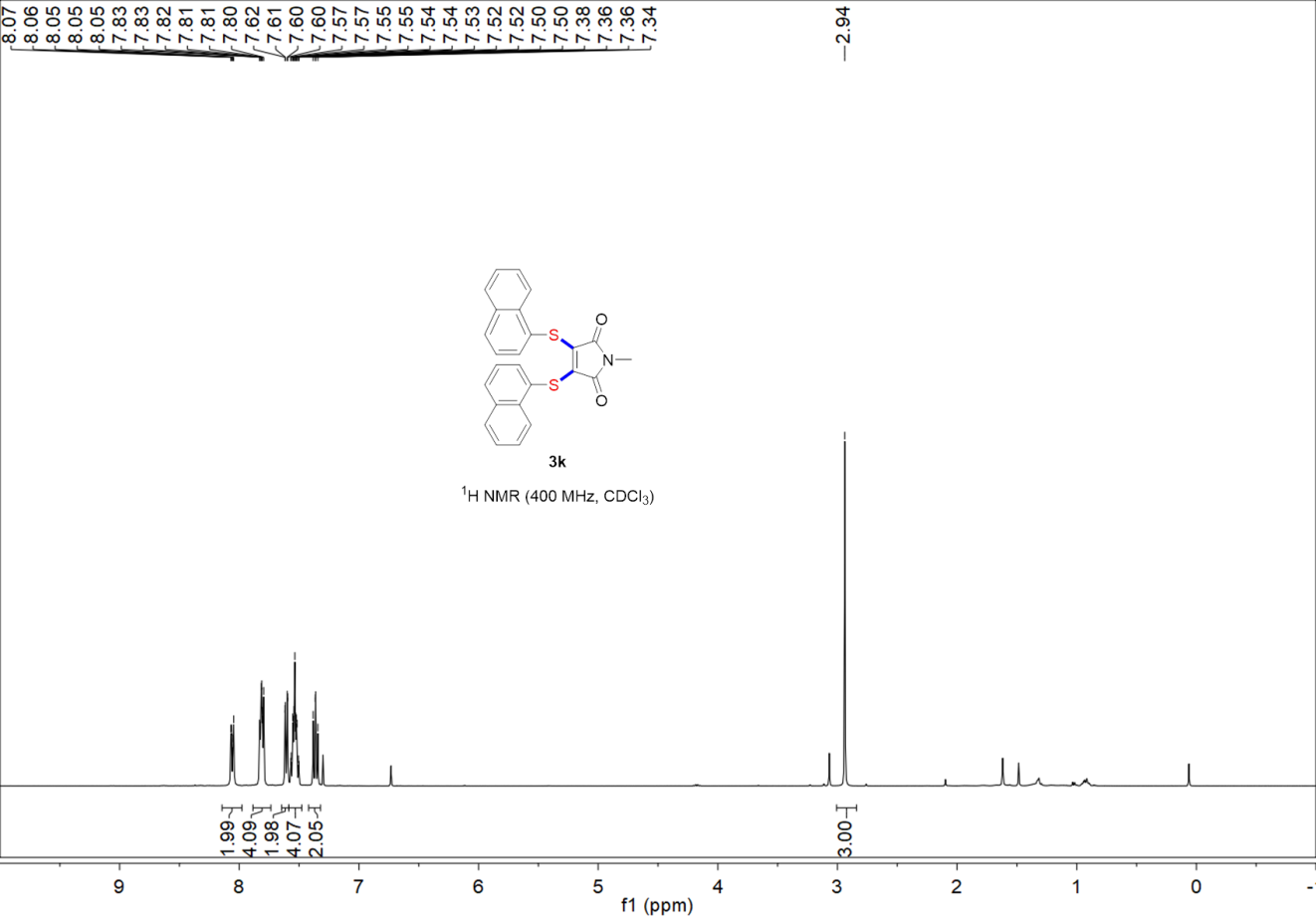
**

**
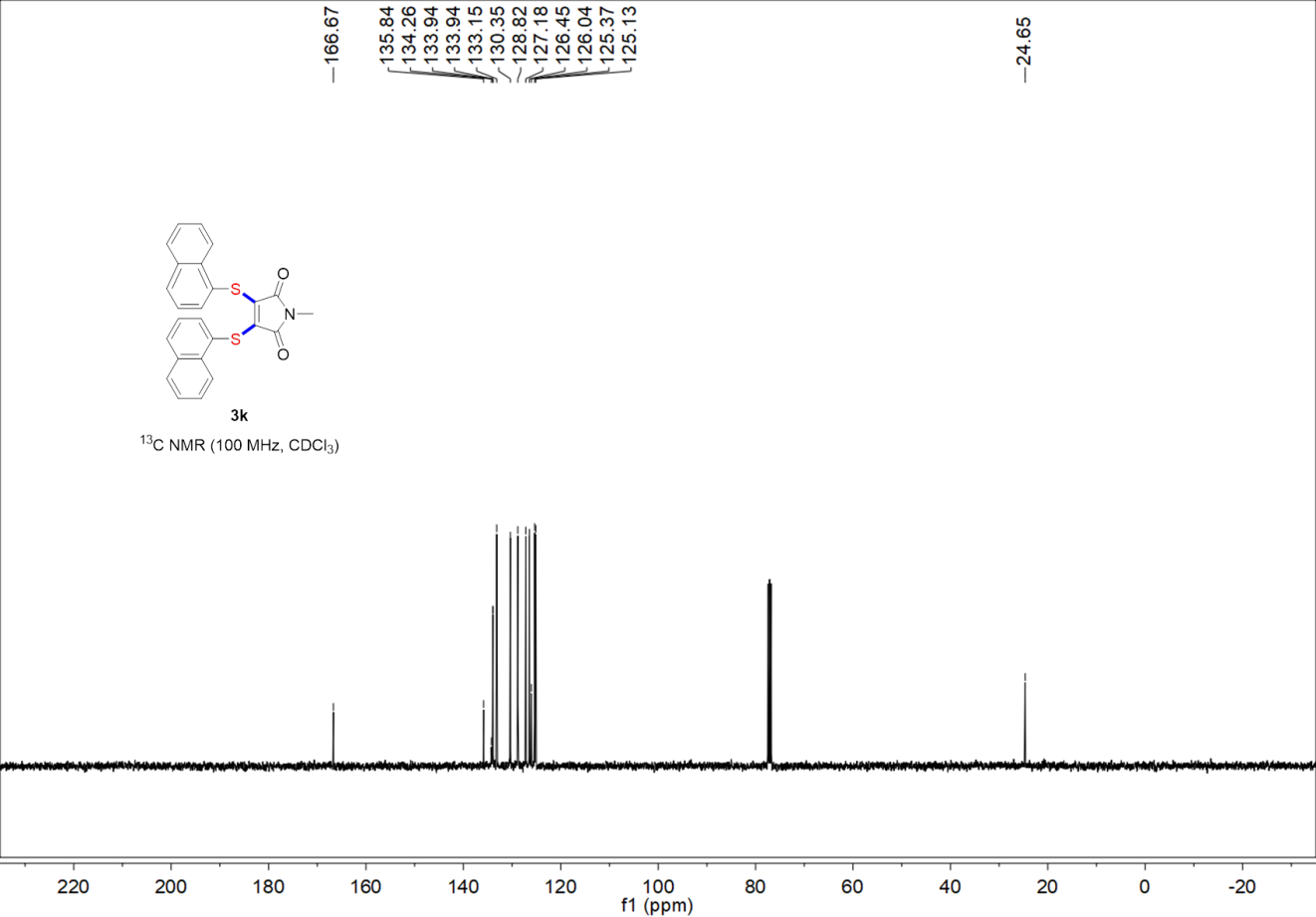
**

**
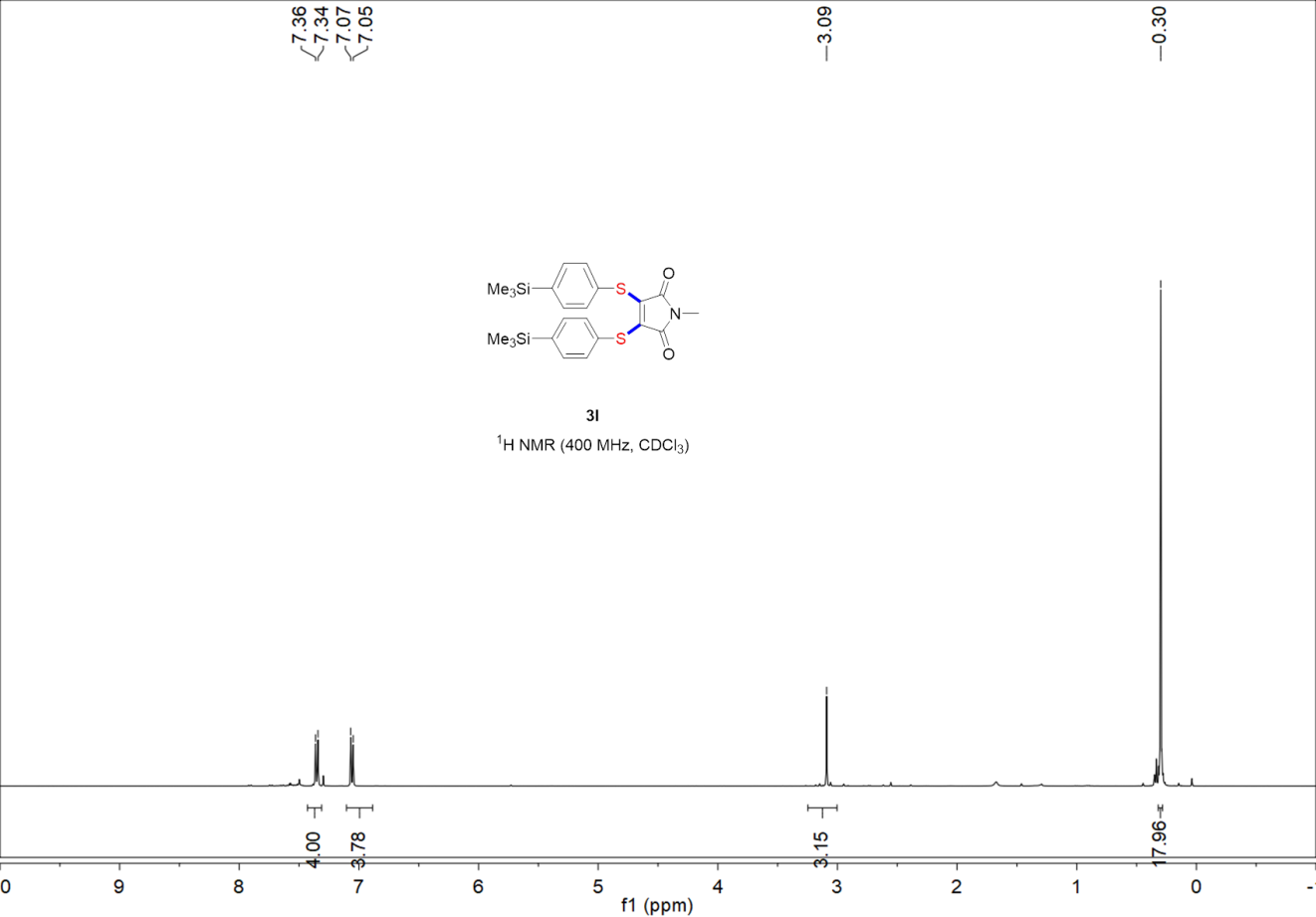
**

**
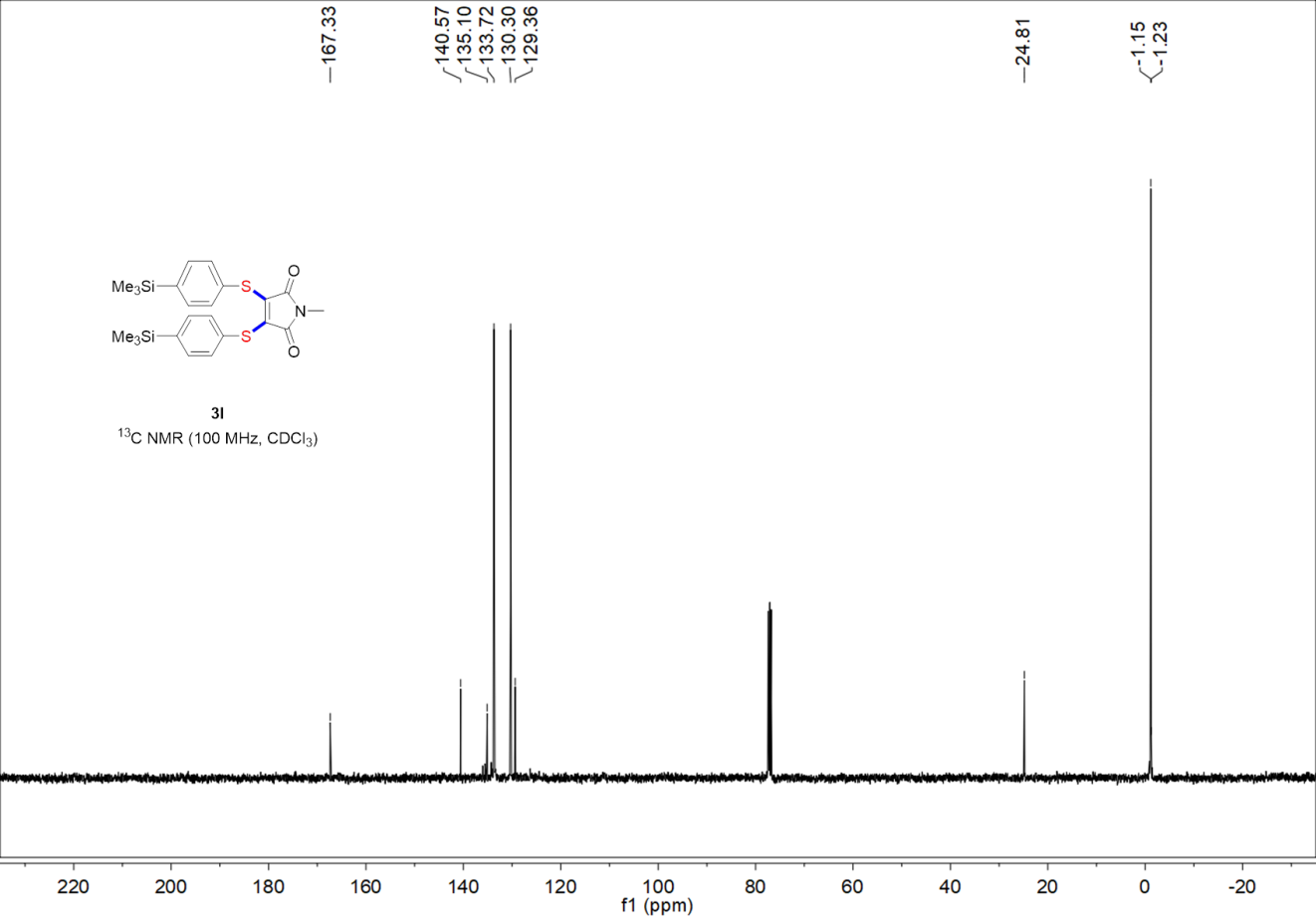
**

**
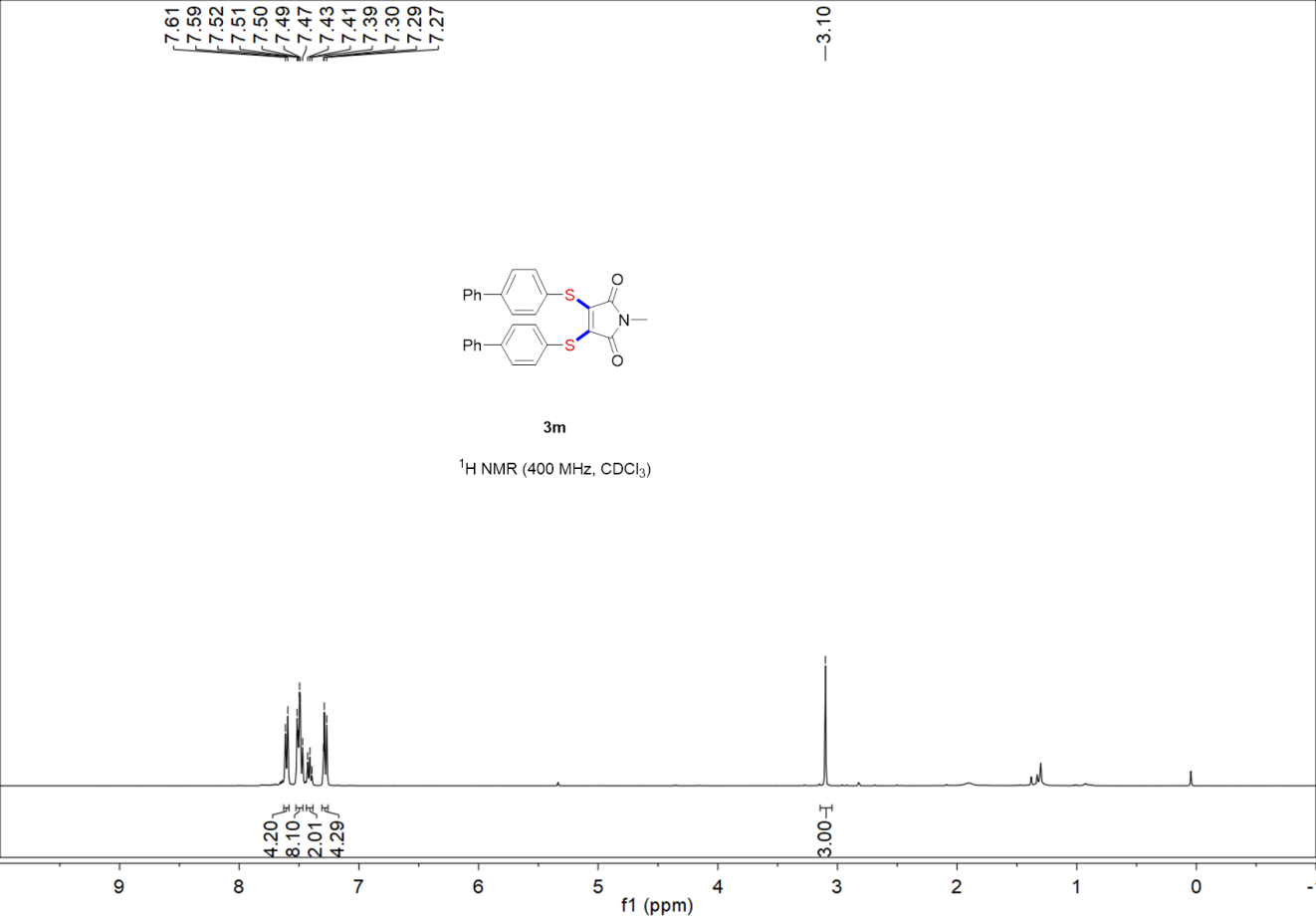
**

**
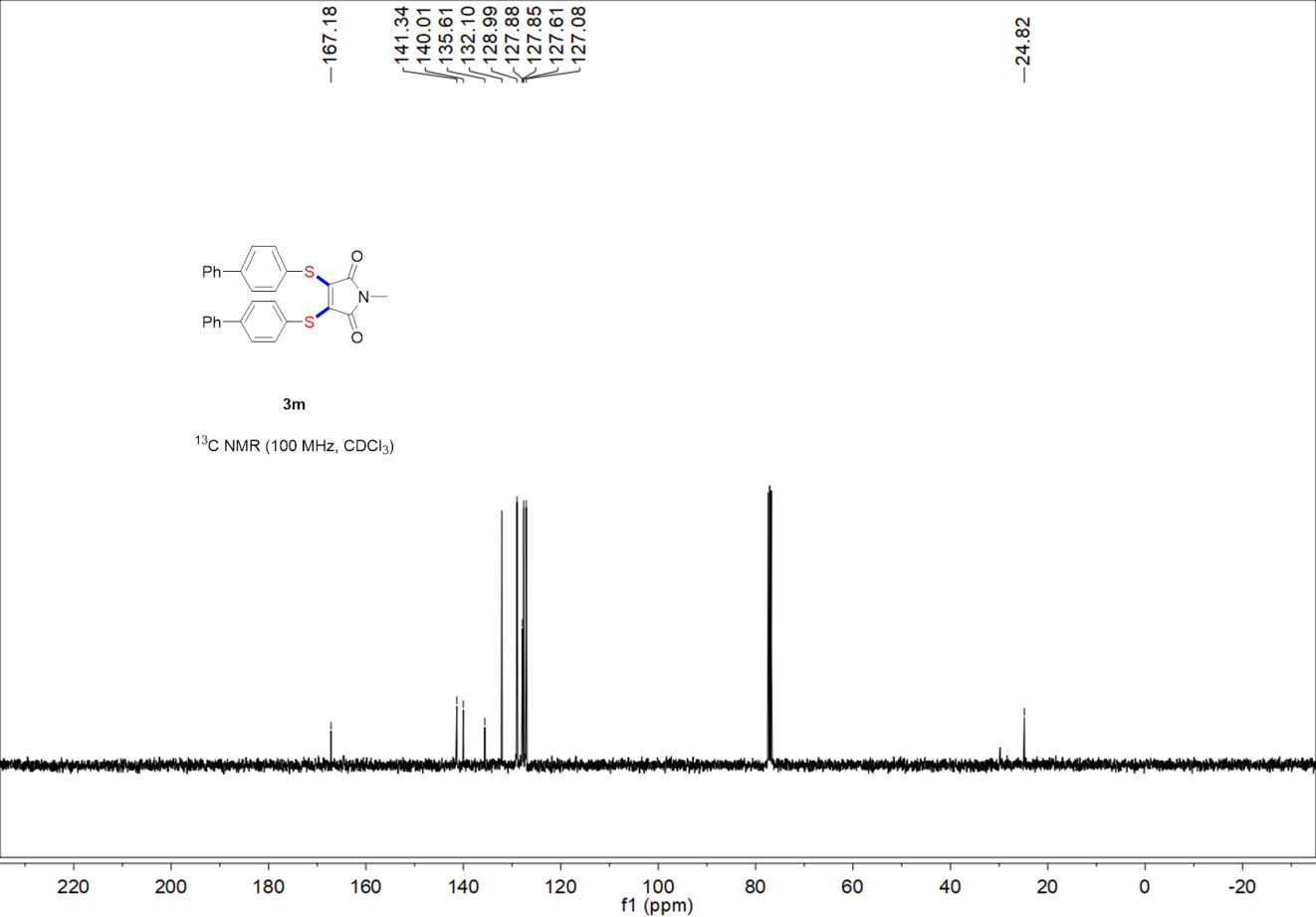
**

**
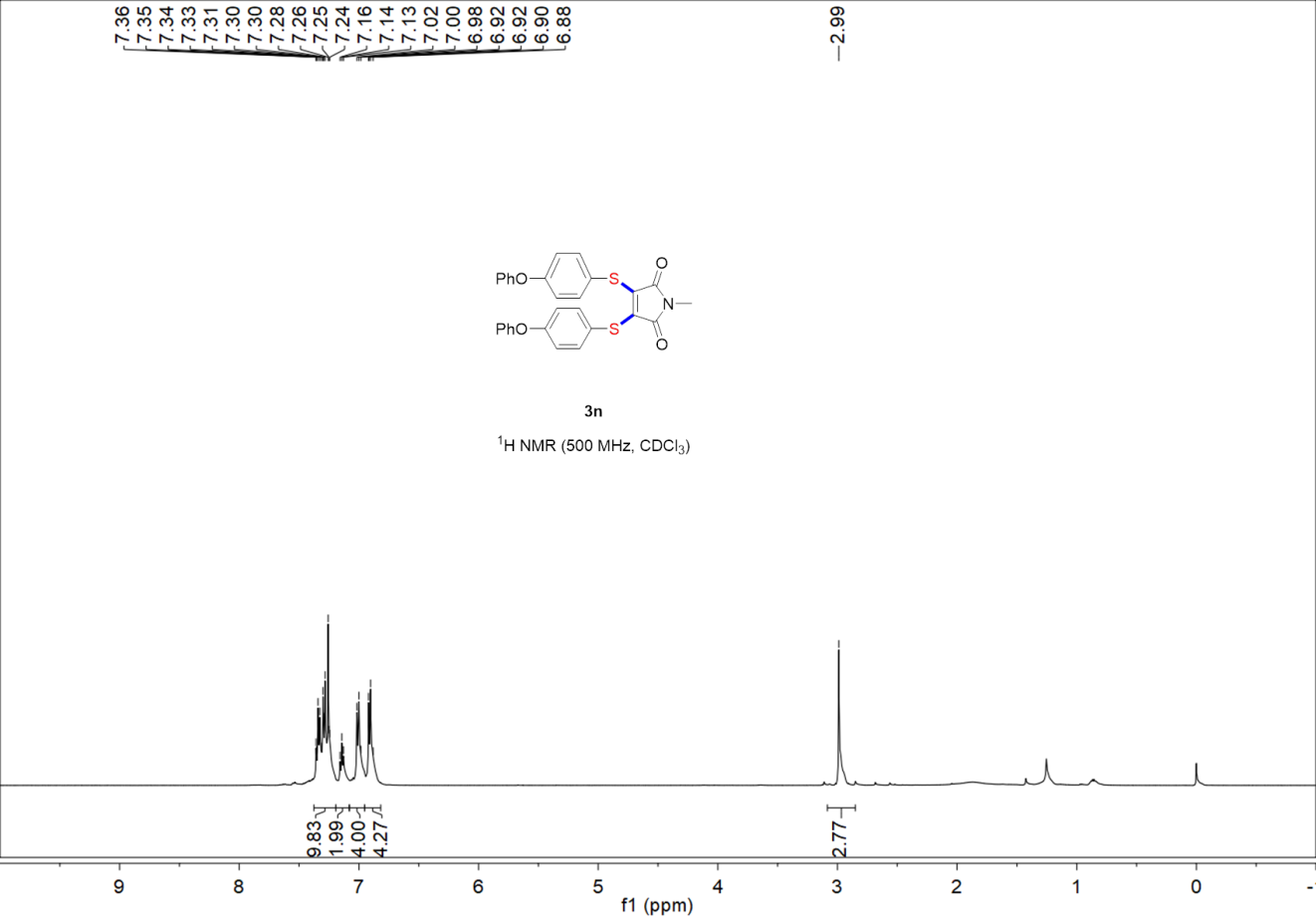
**

**
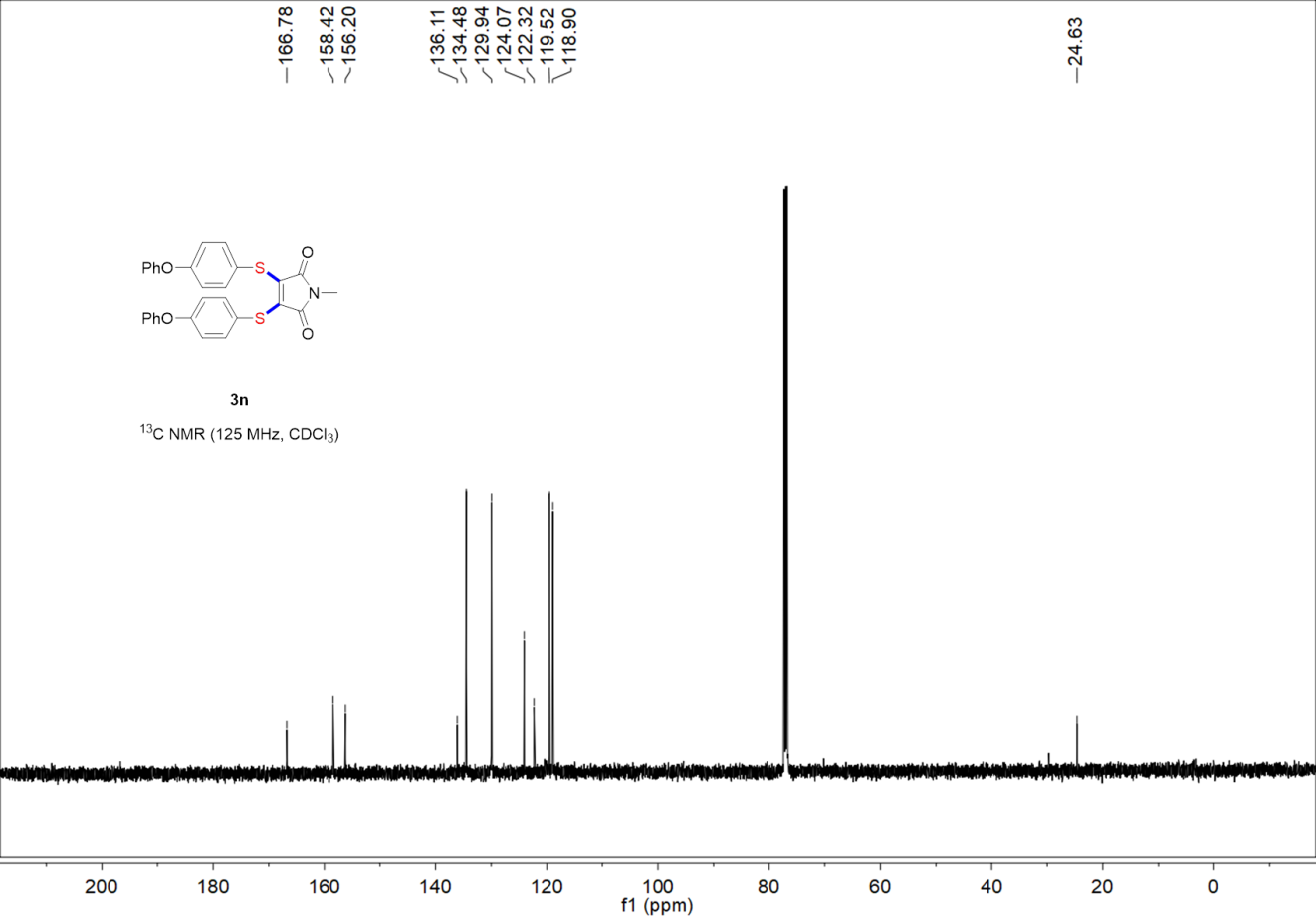
**

**
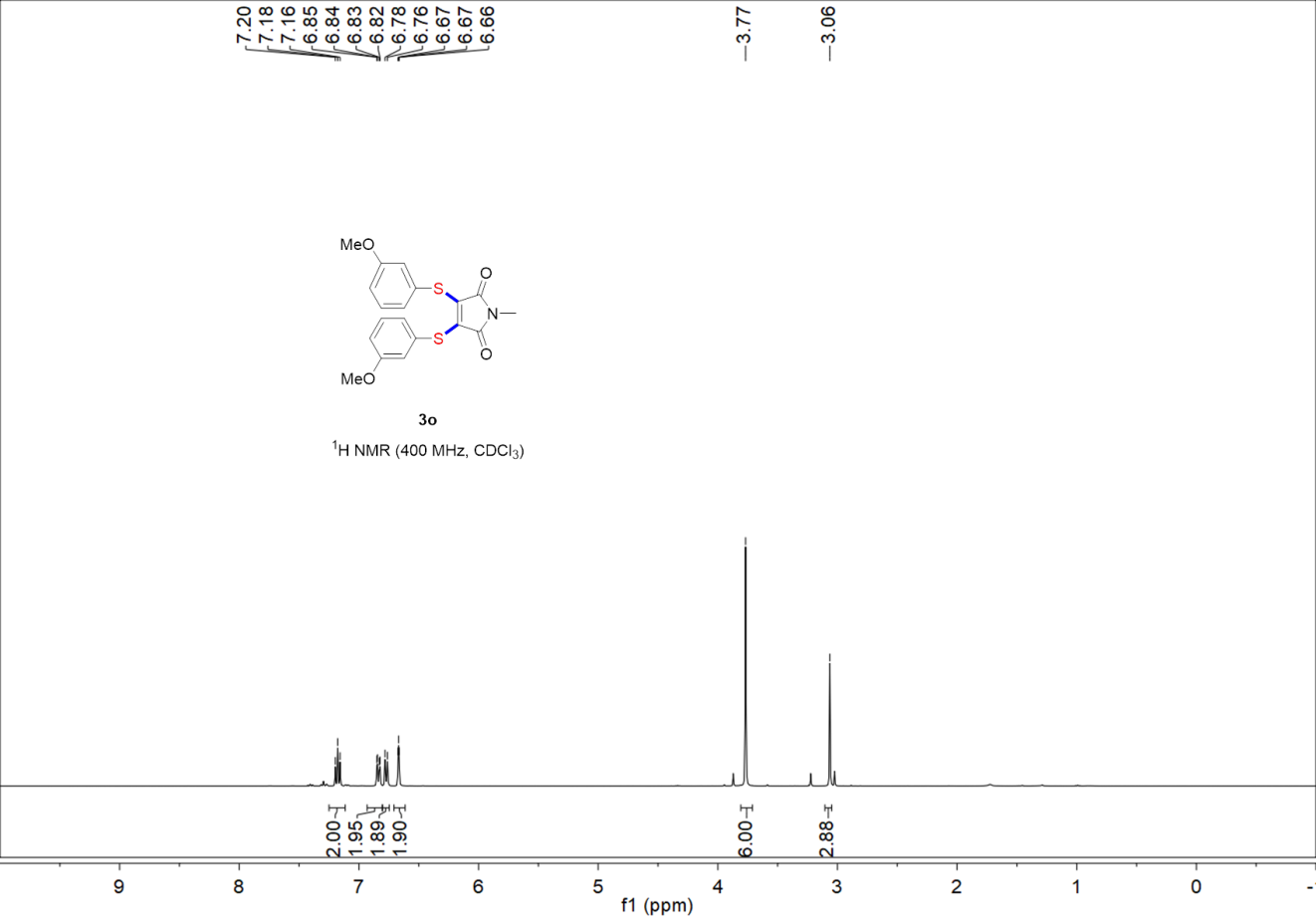
**

**
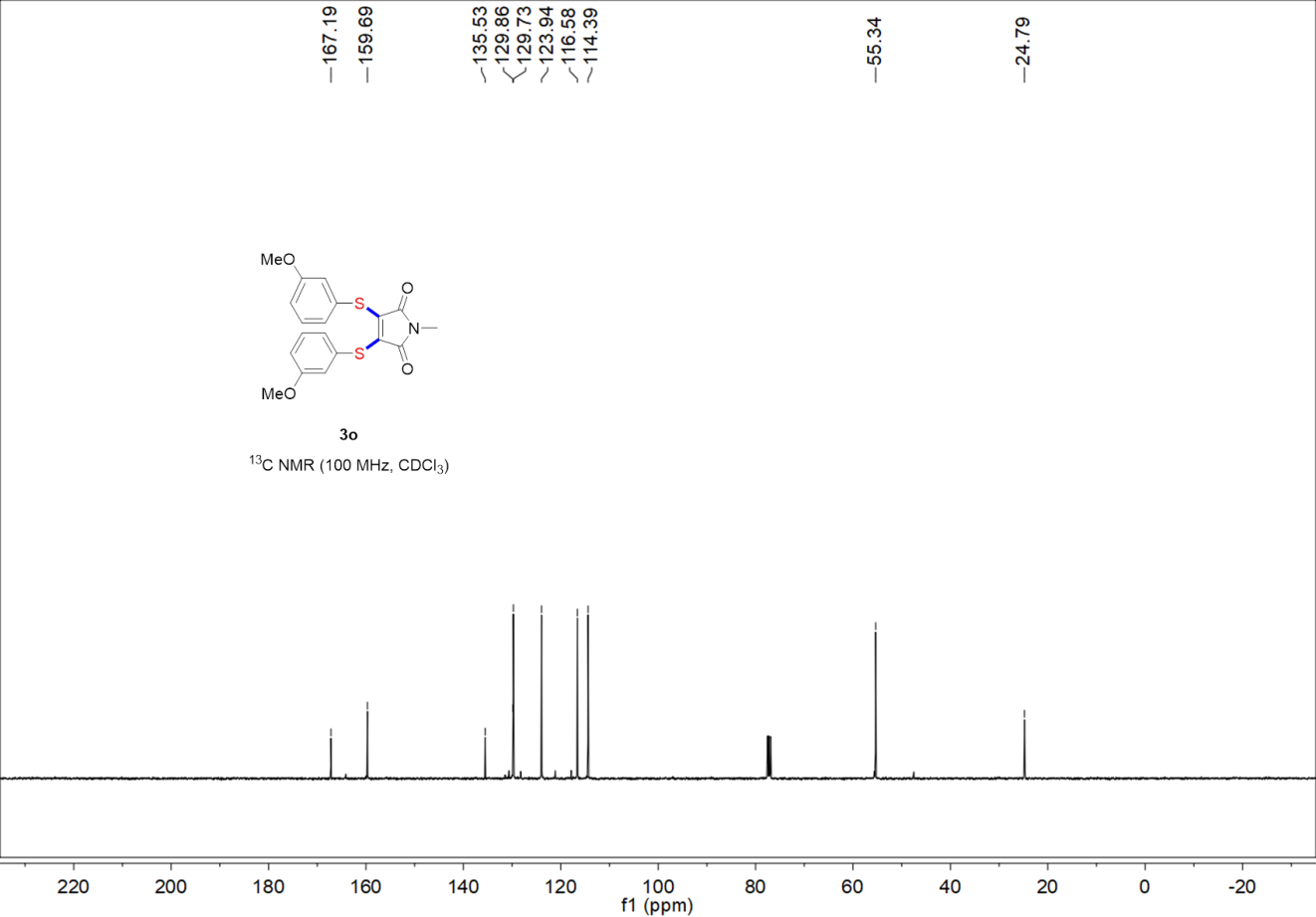
**

**
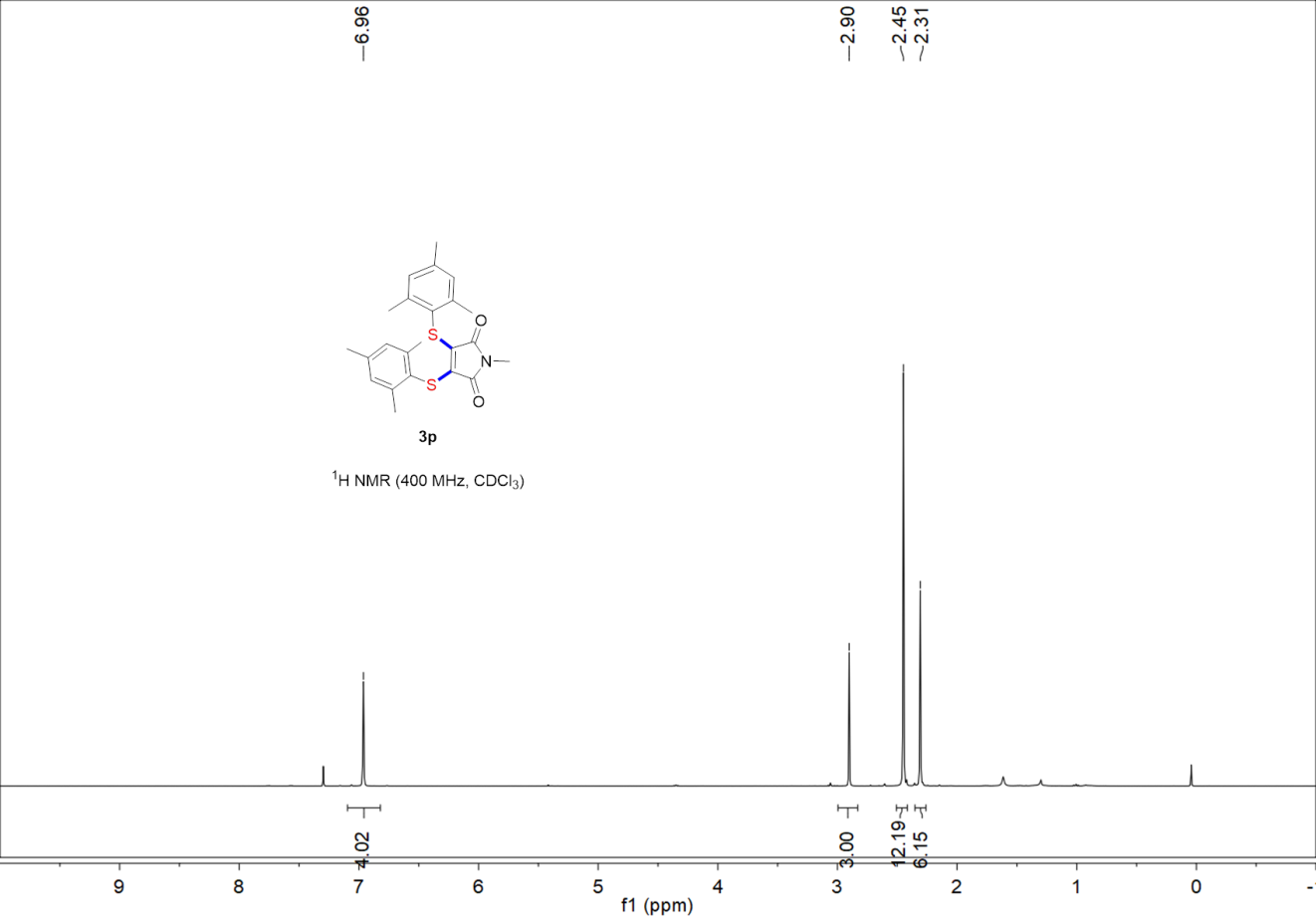
**

**
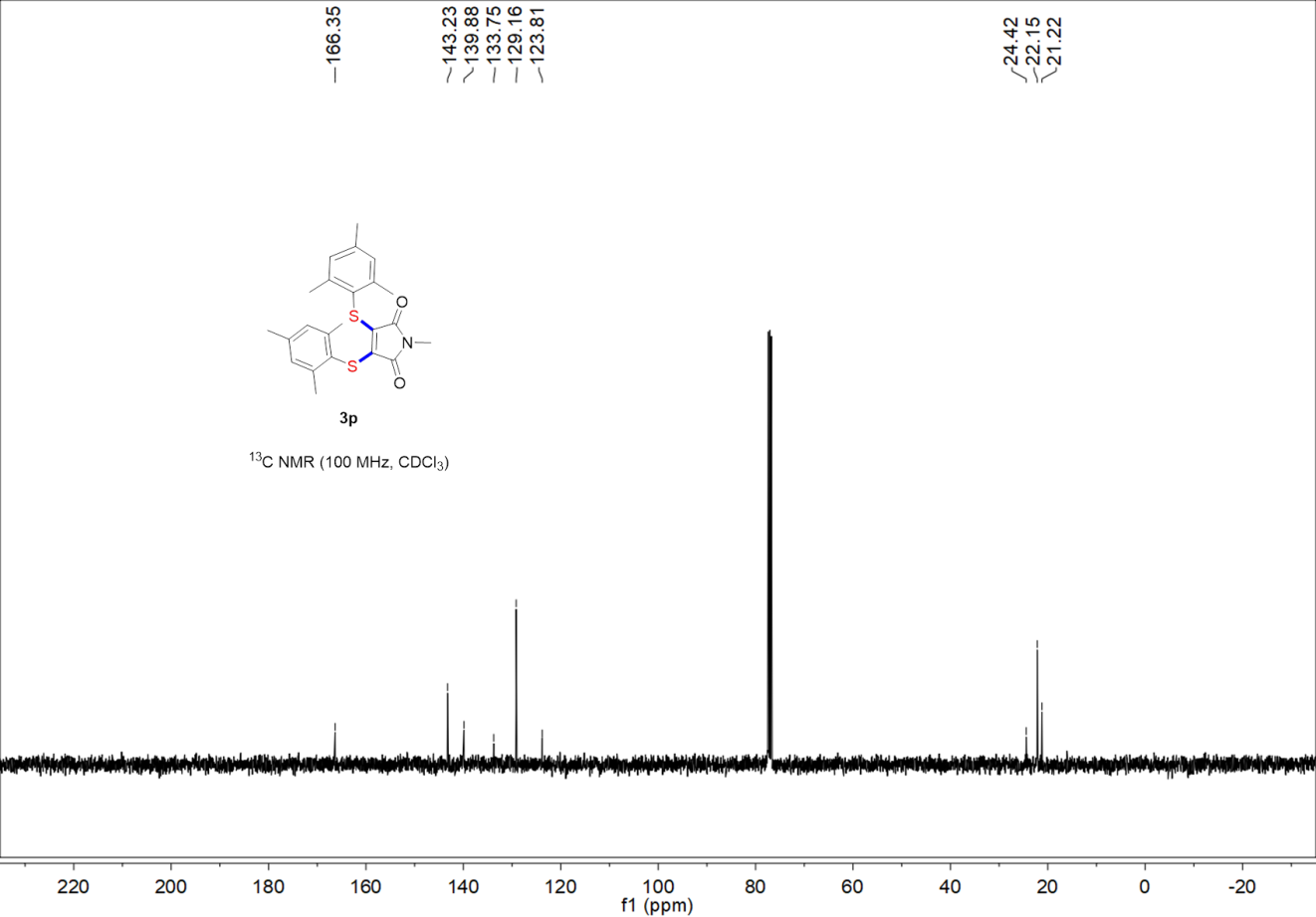
**

**
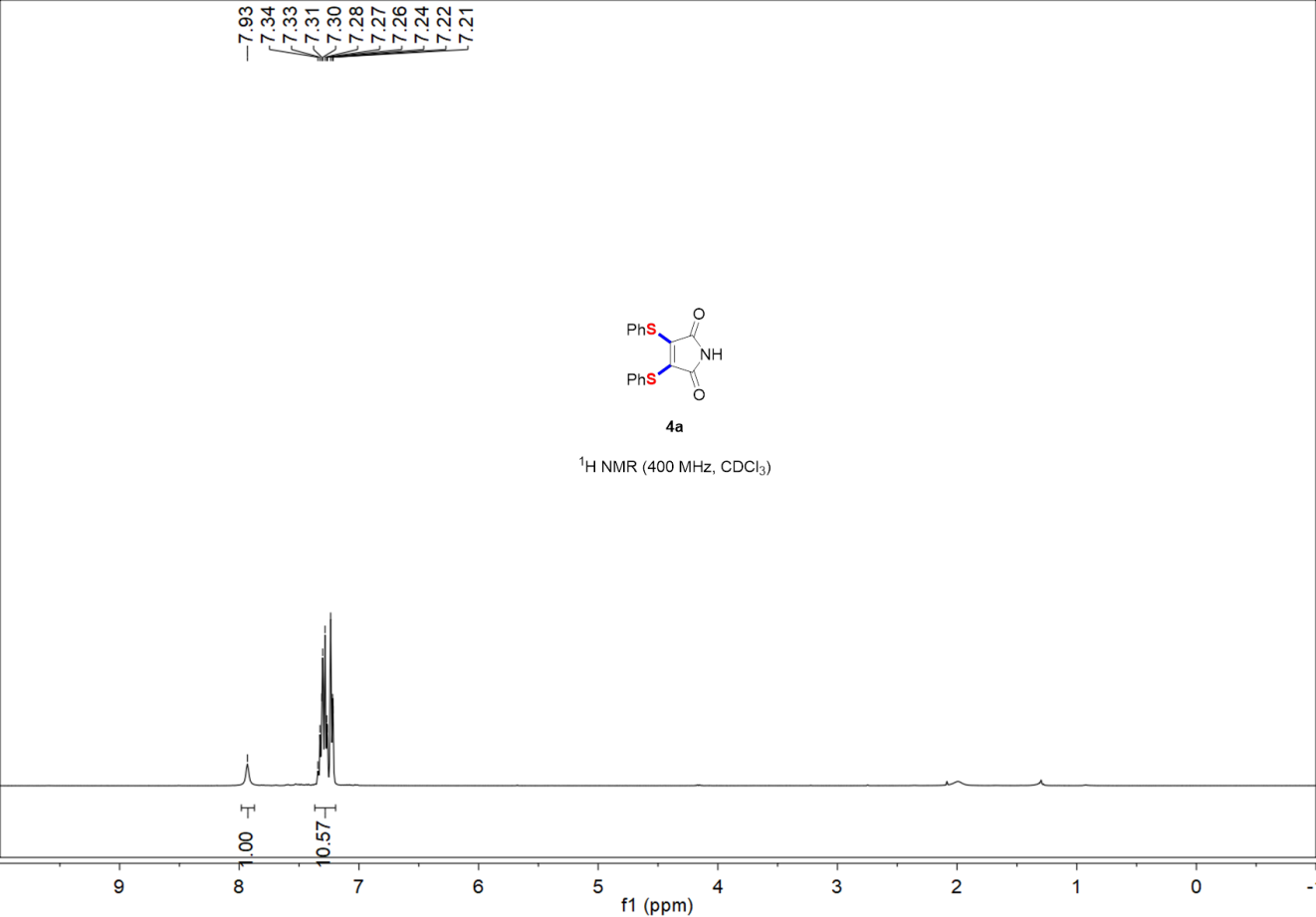
**

**
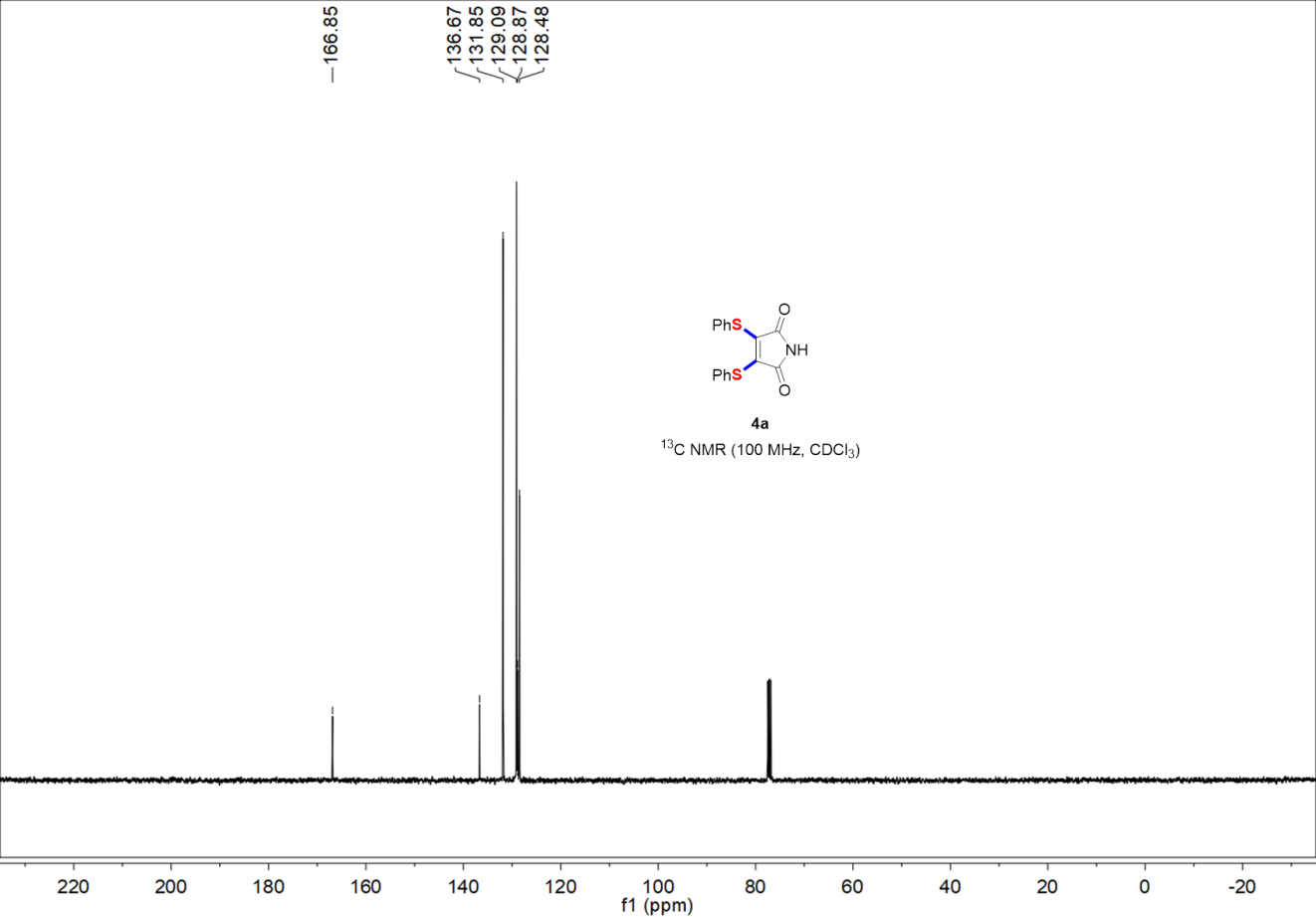
**

**
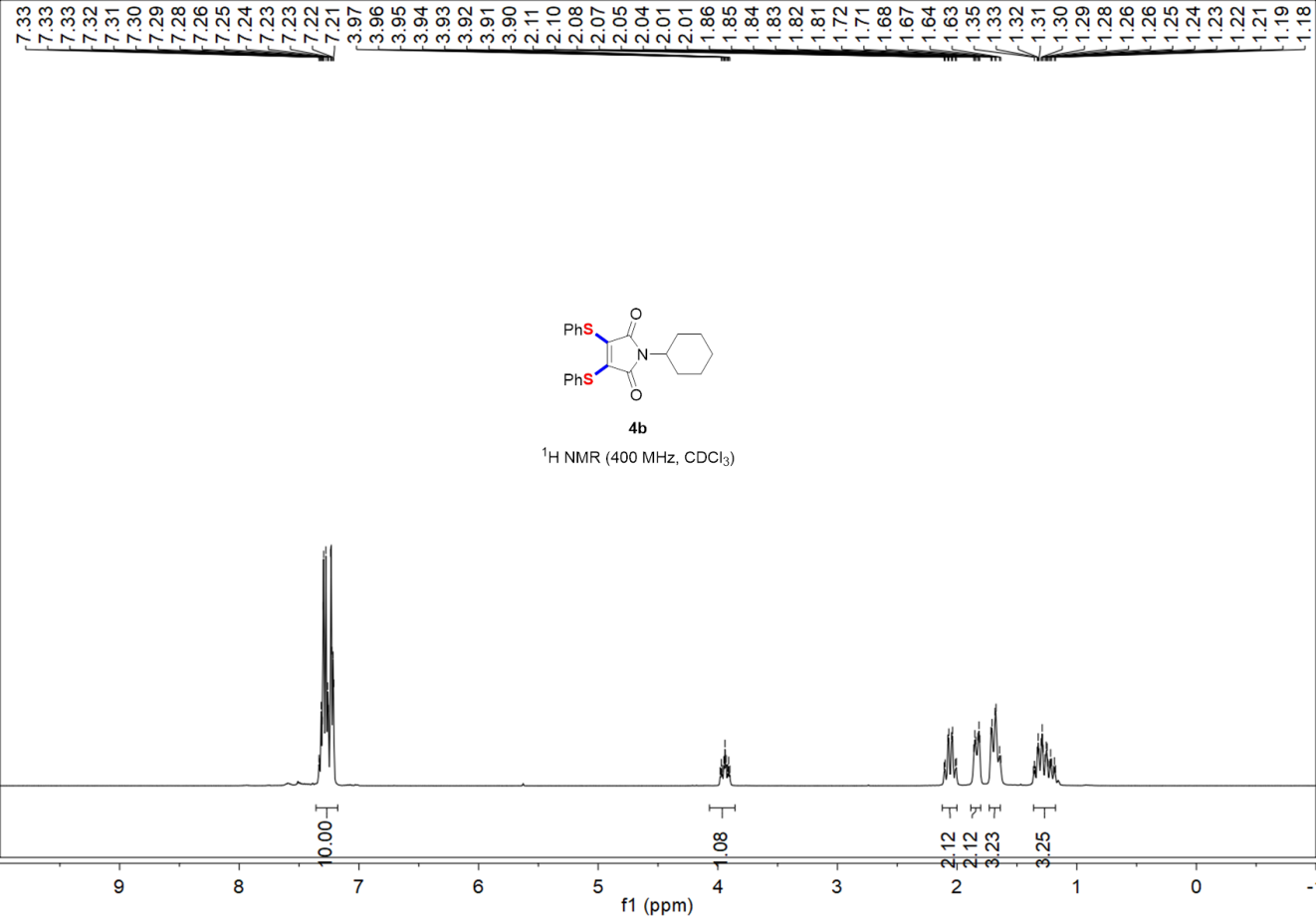
**

**
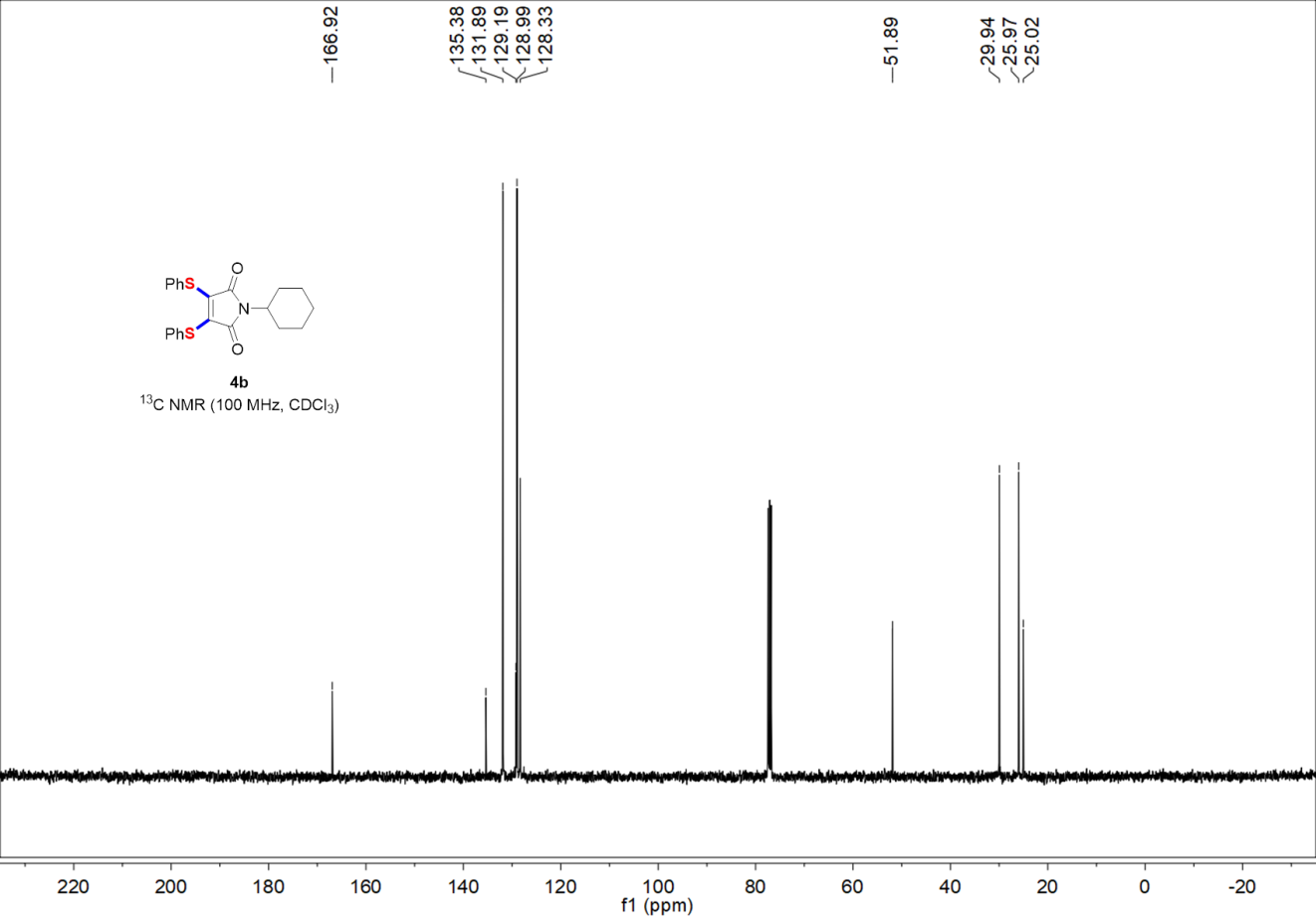
**

**
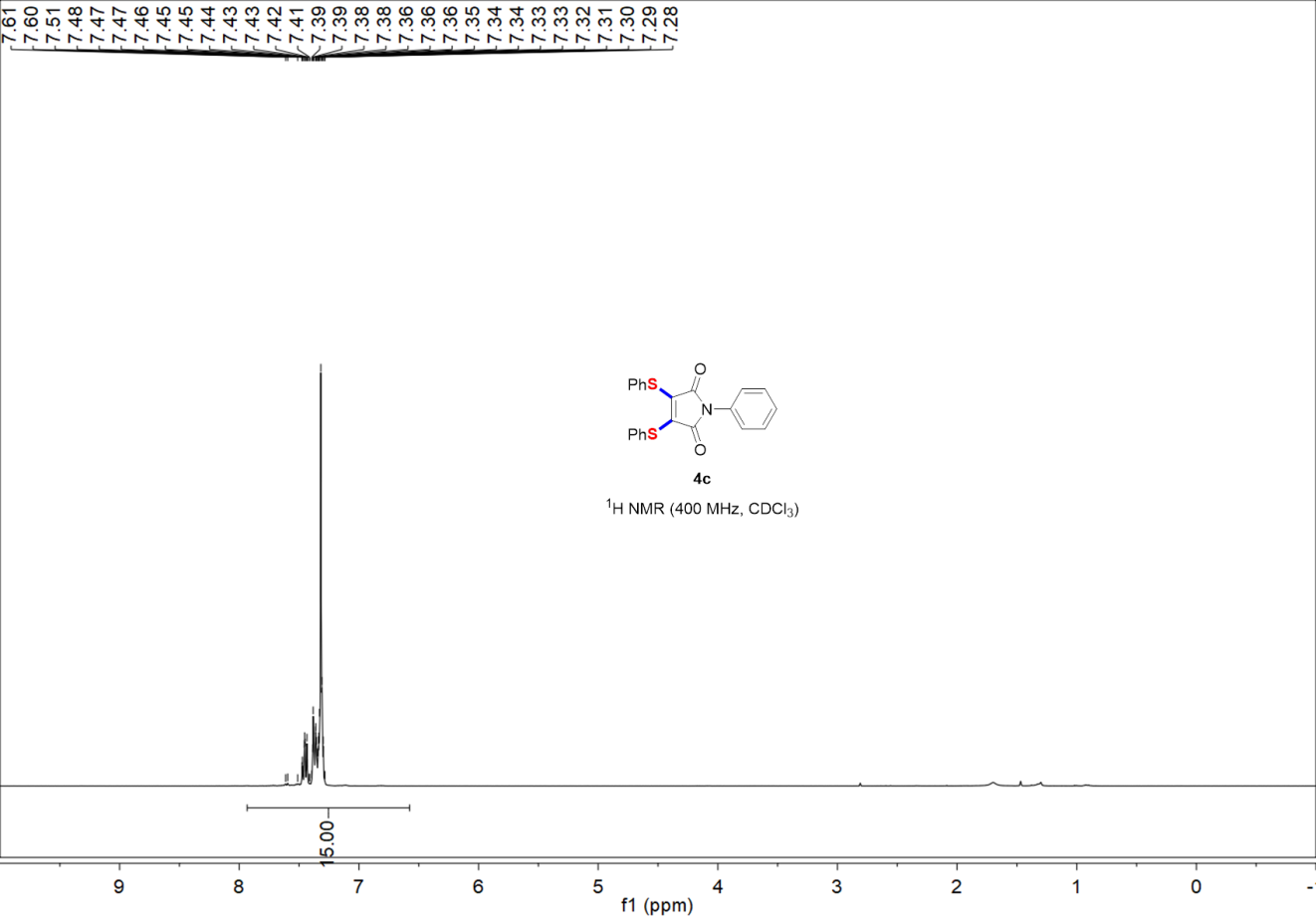
**

**
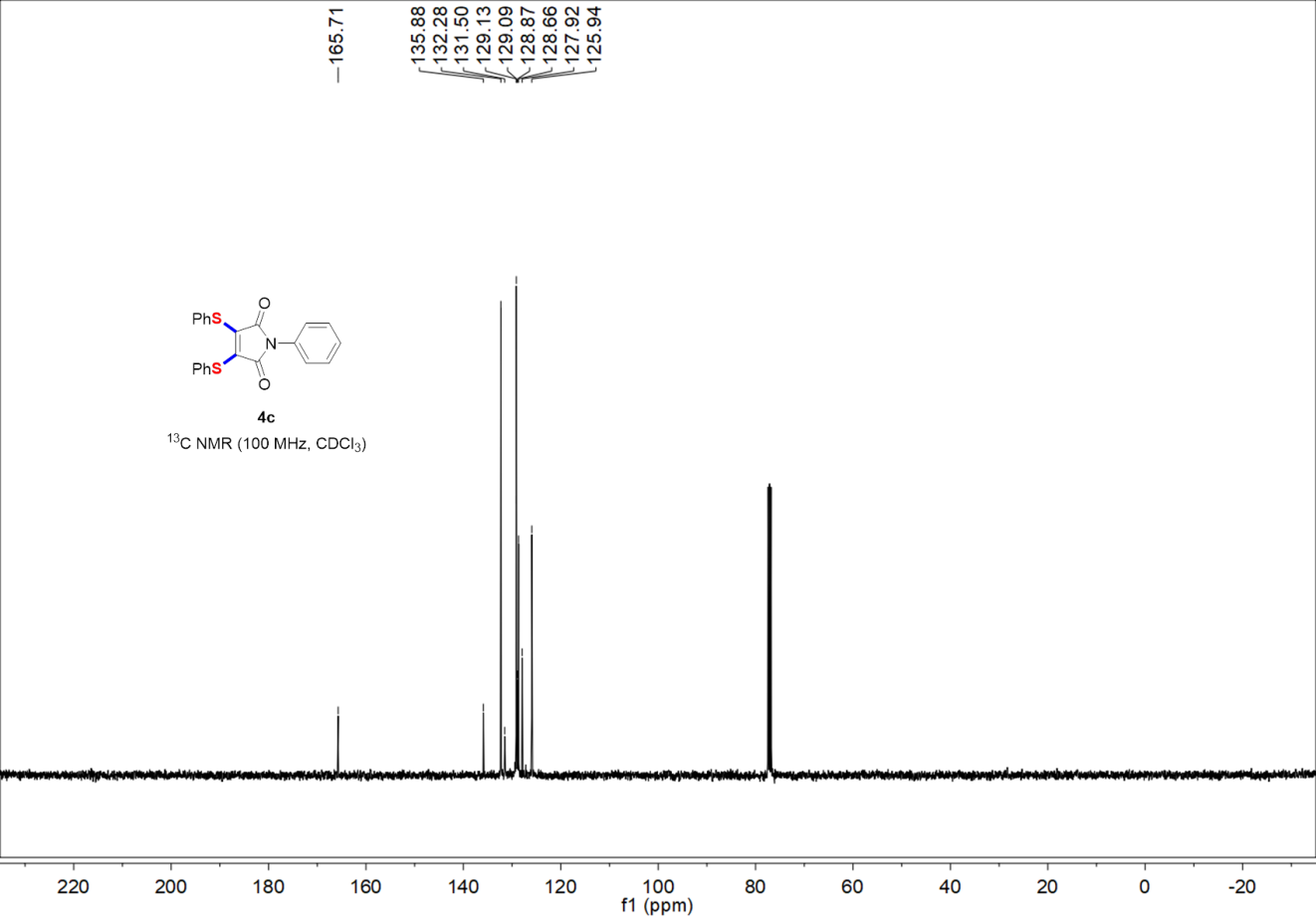
**

**
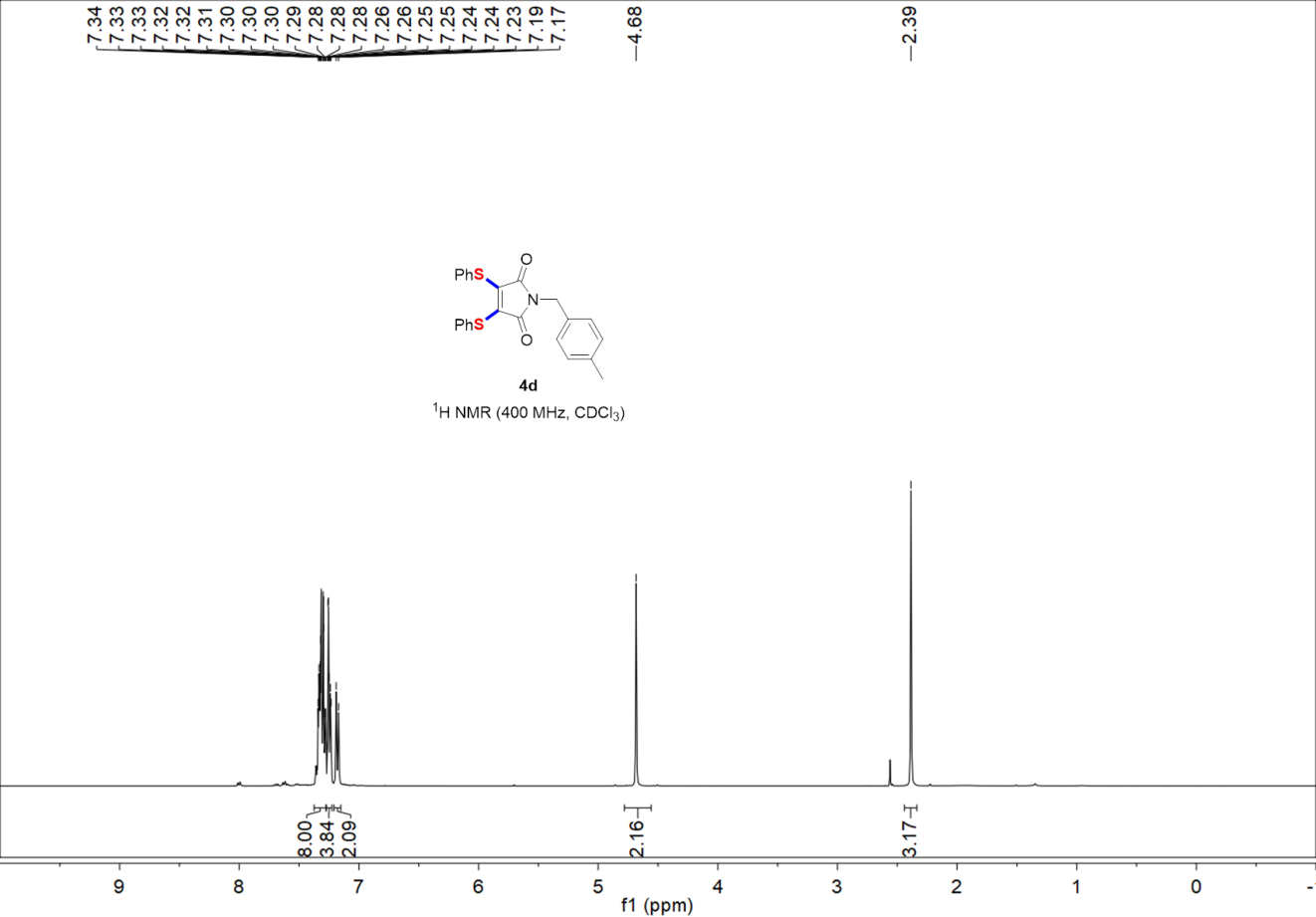
**

**
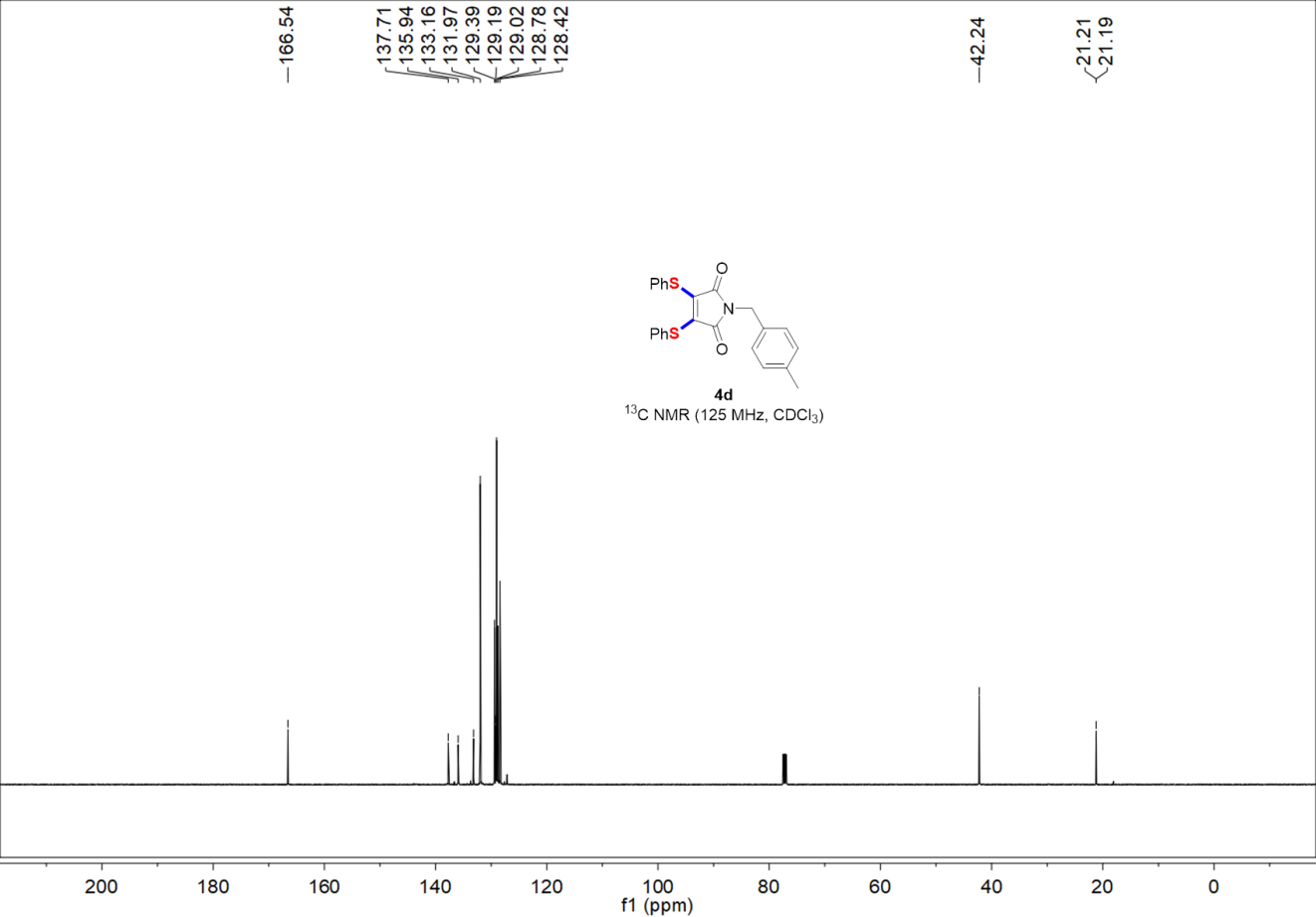
**

**
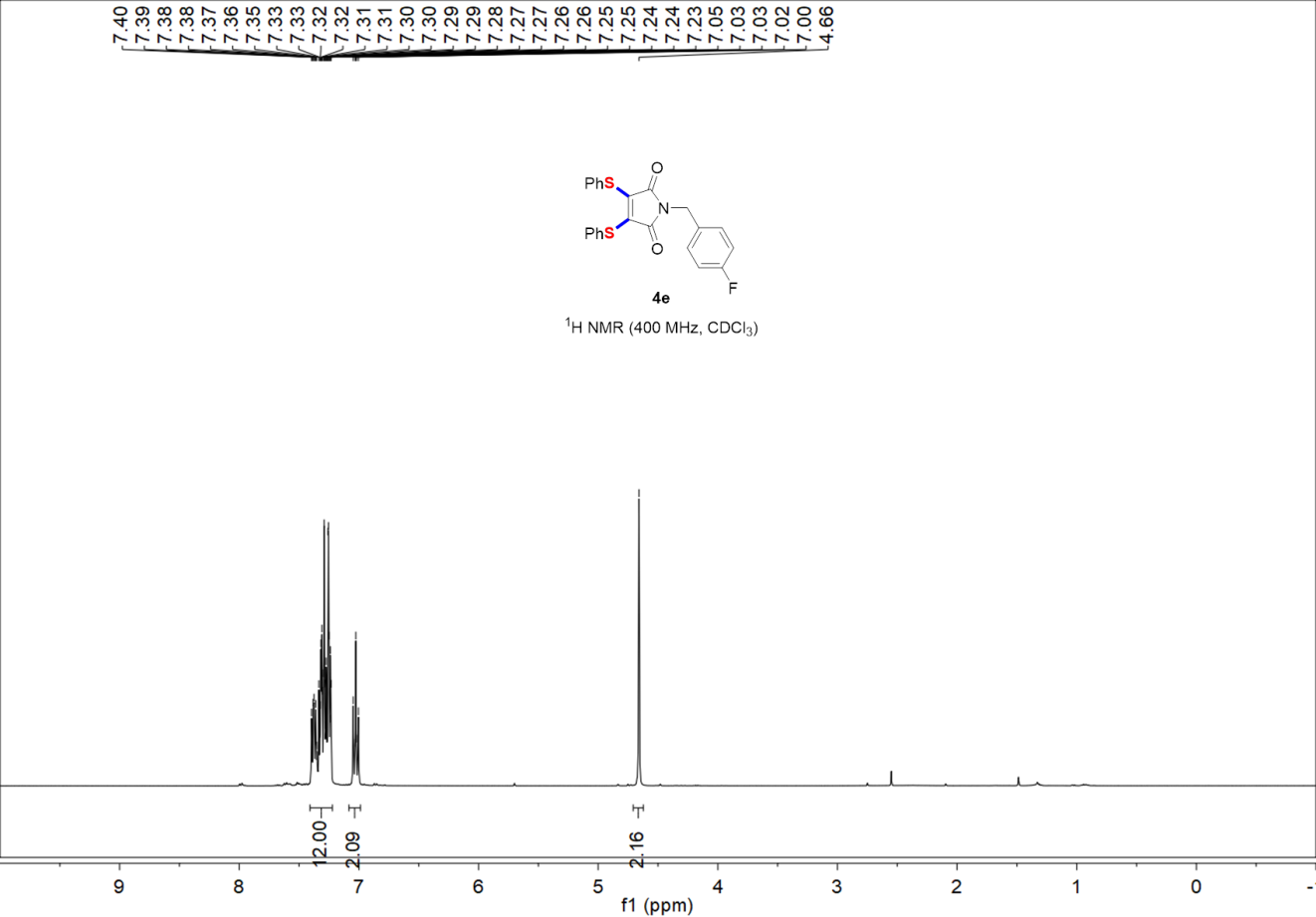
**

**
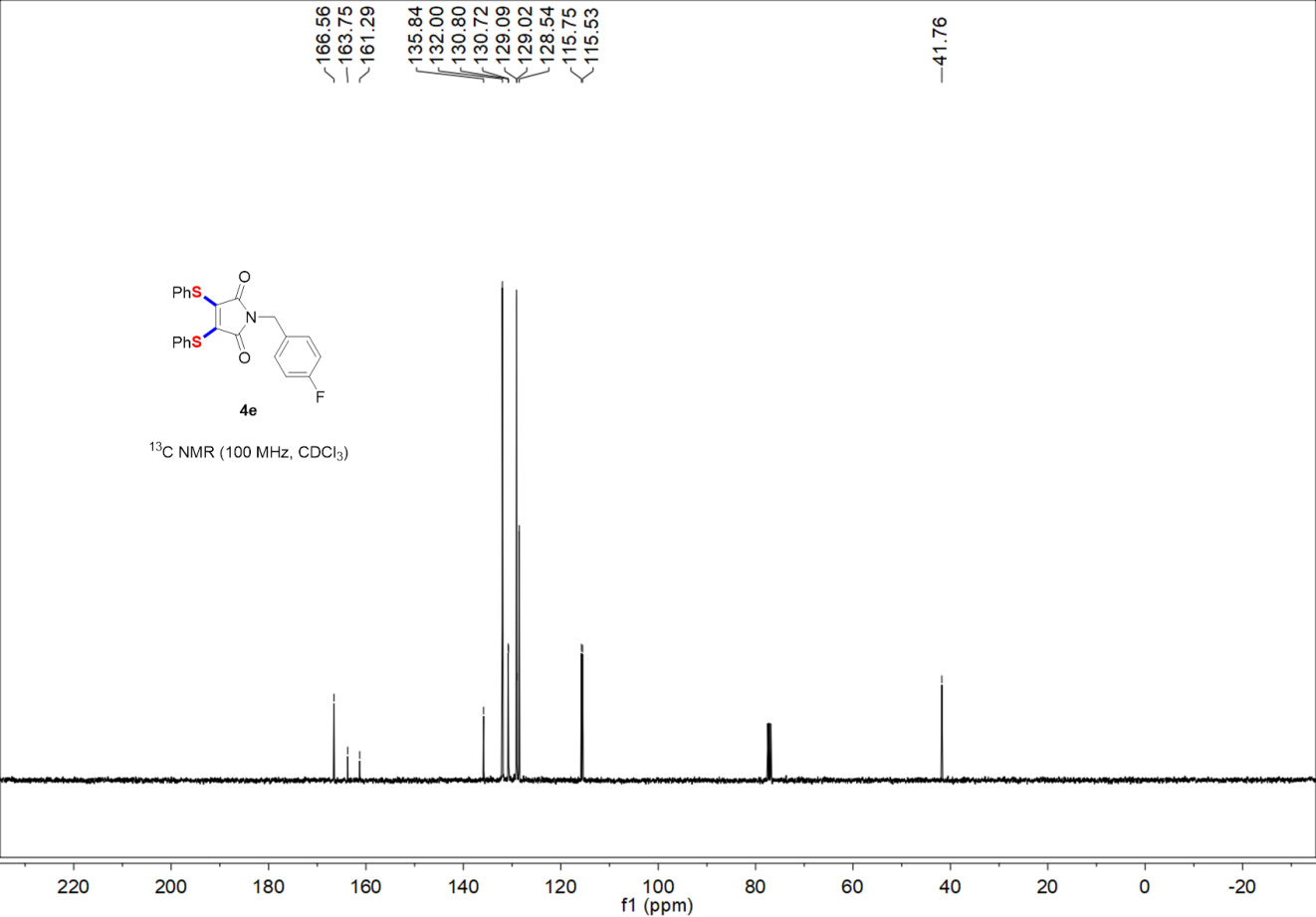
**

**
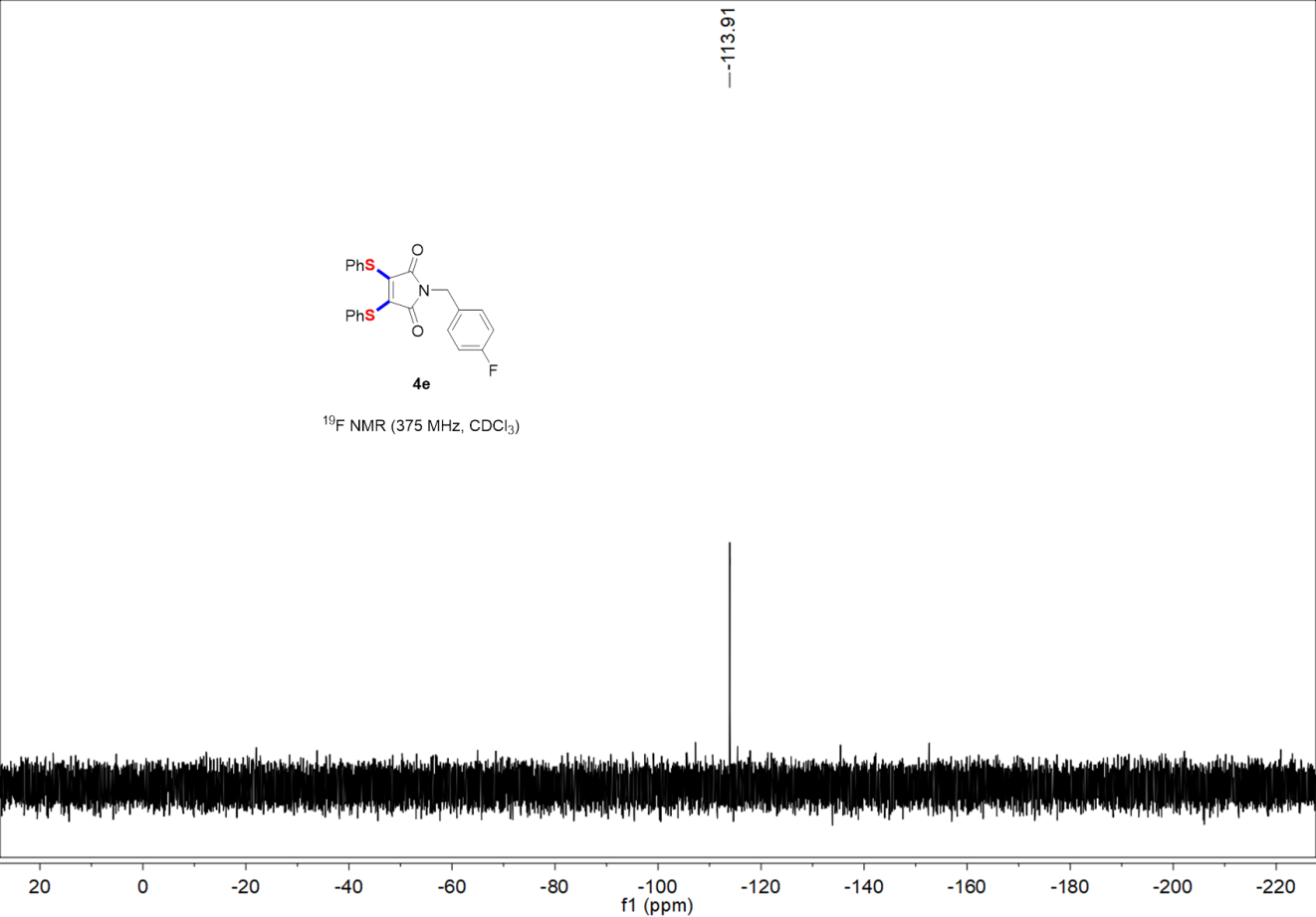
**

**
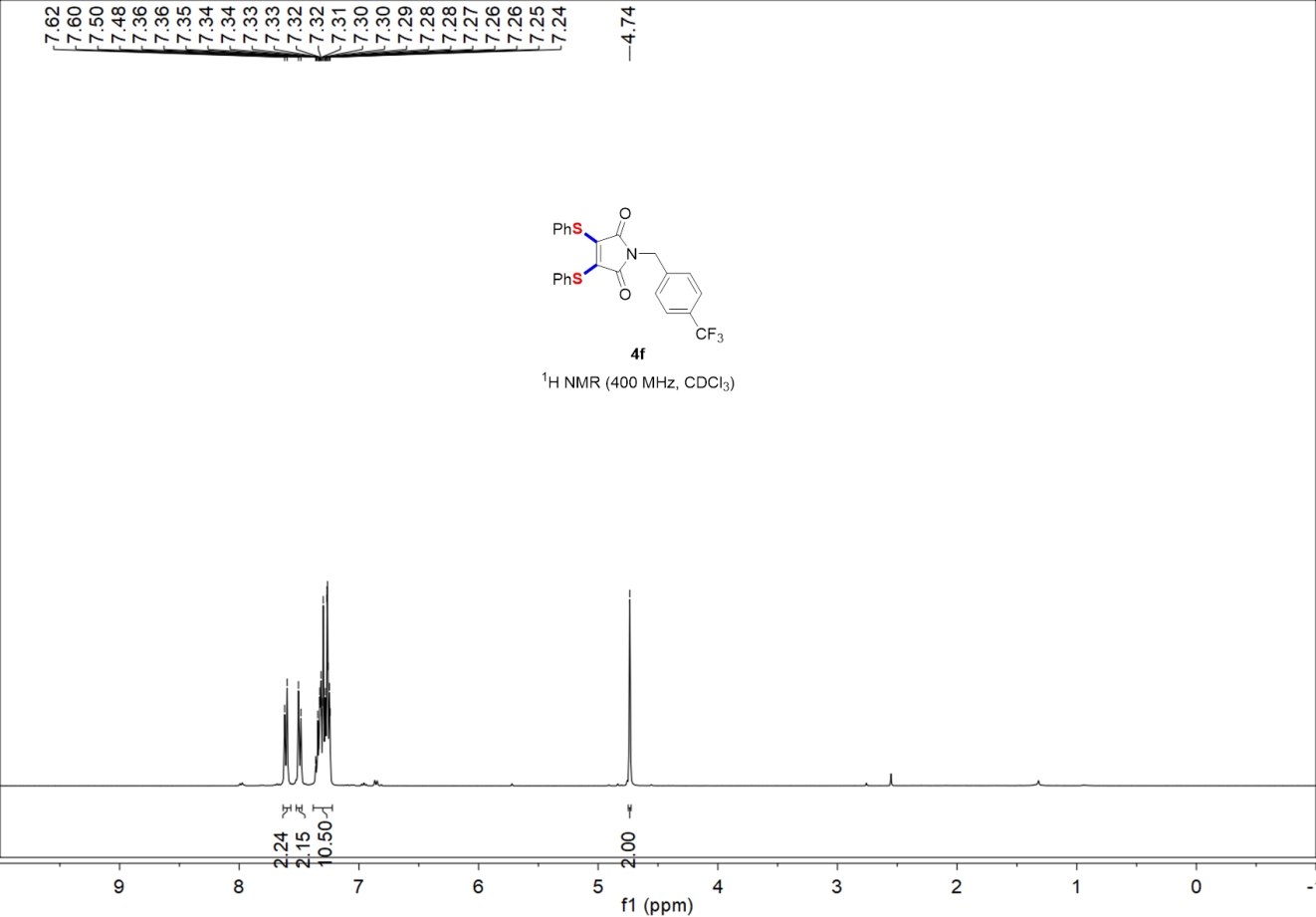
**

**
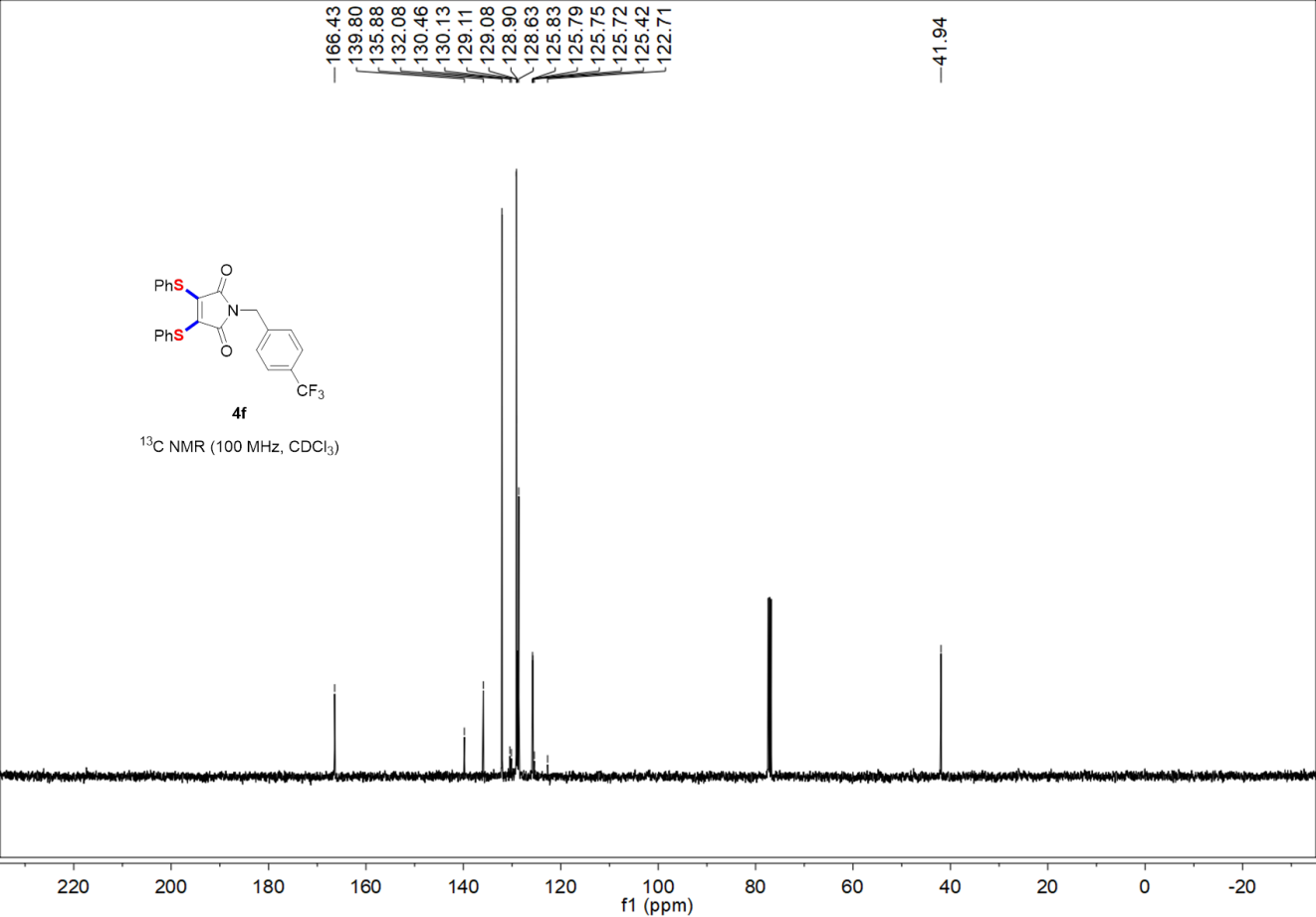
**

**
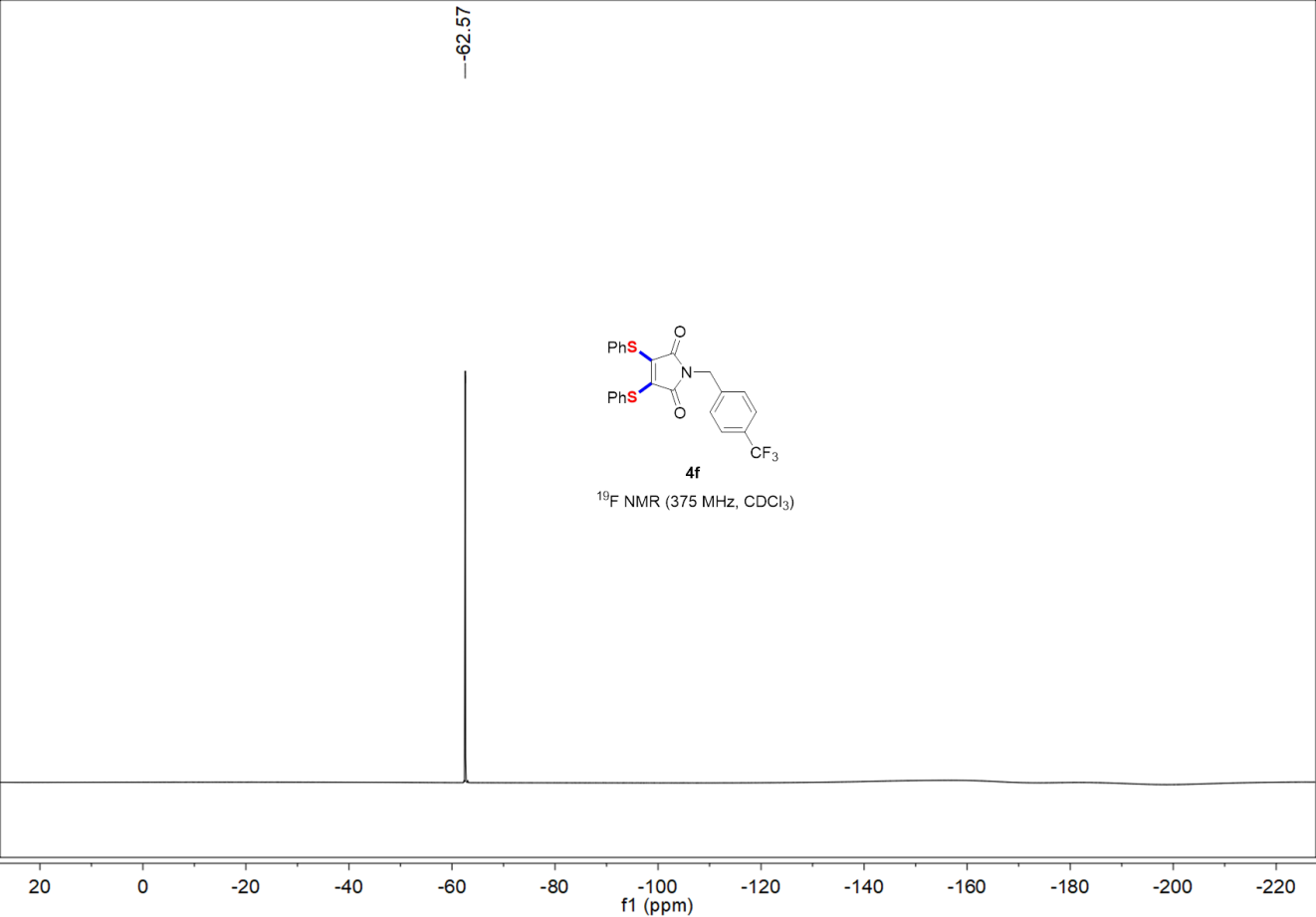
**

**
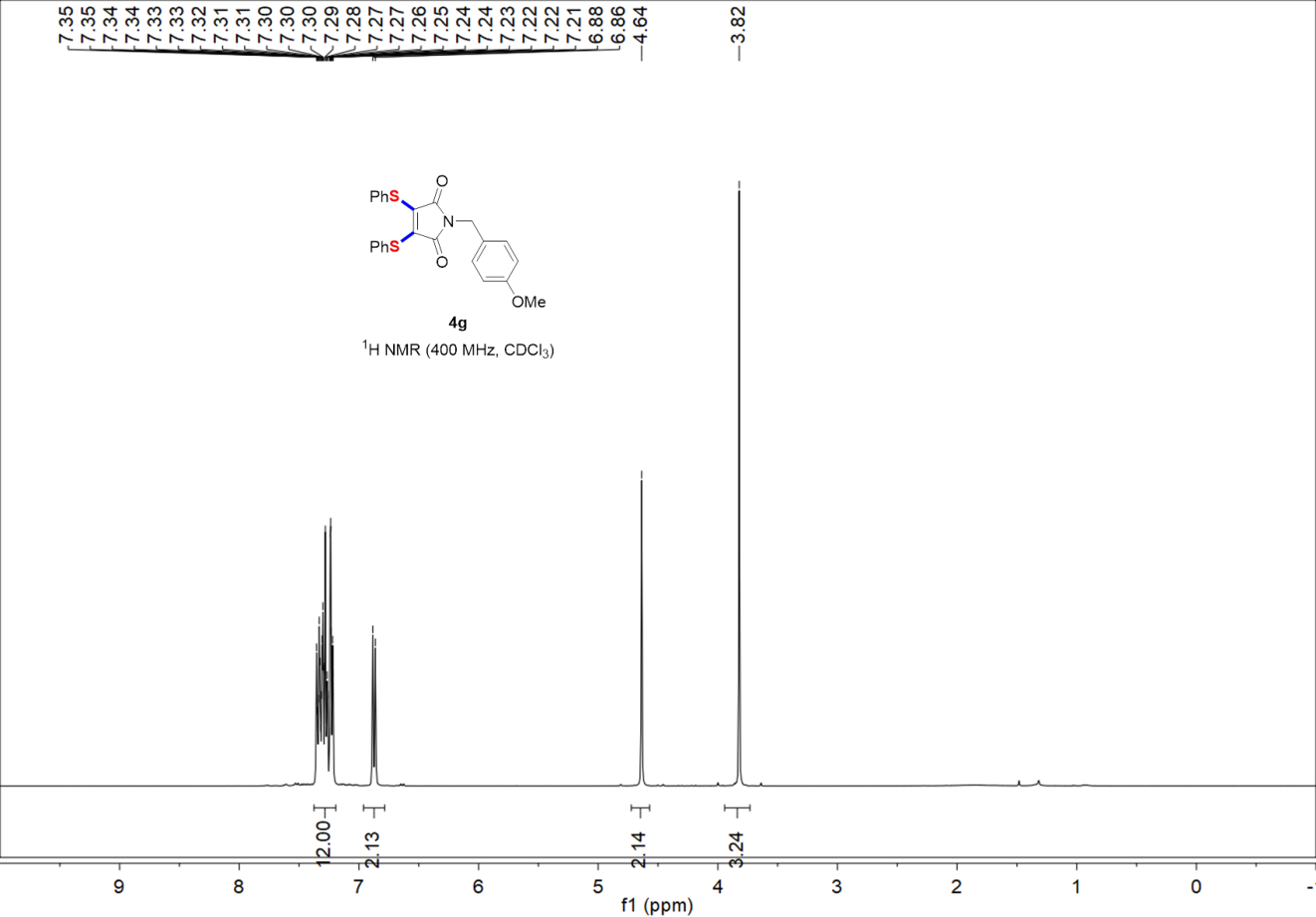
**

**
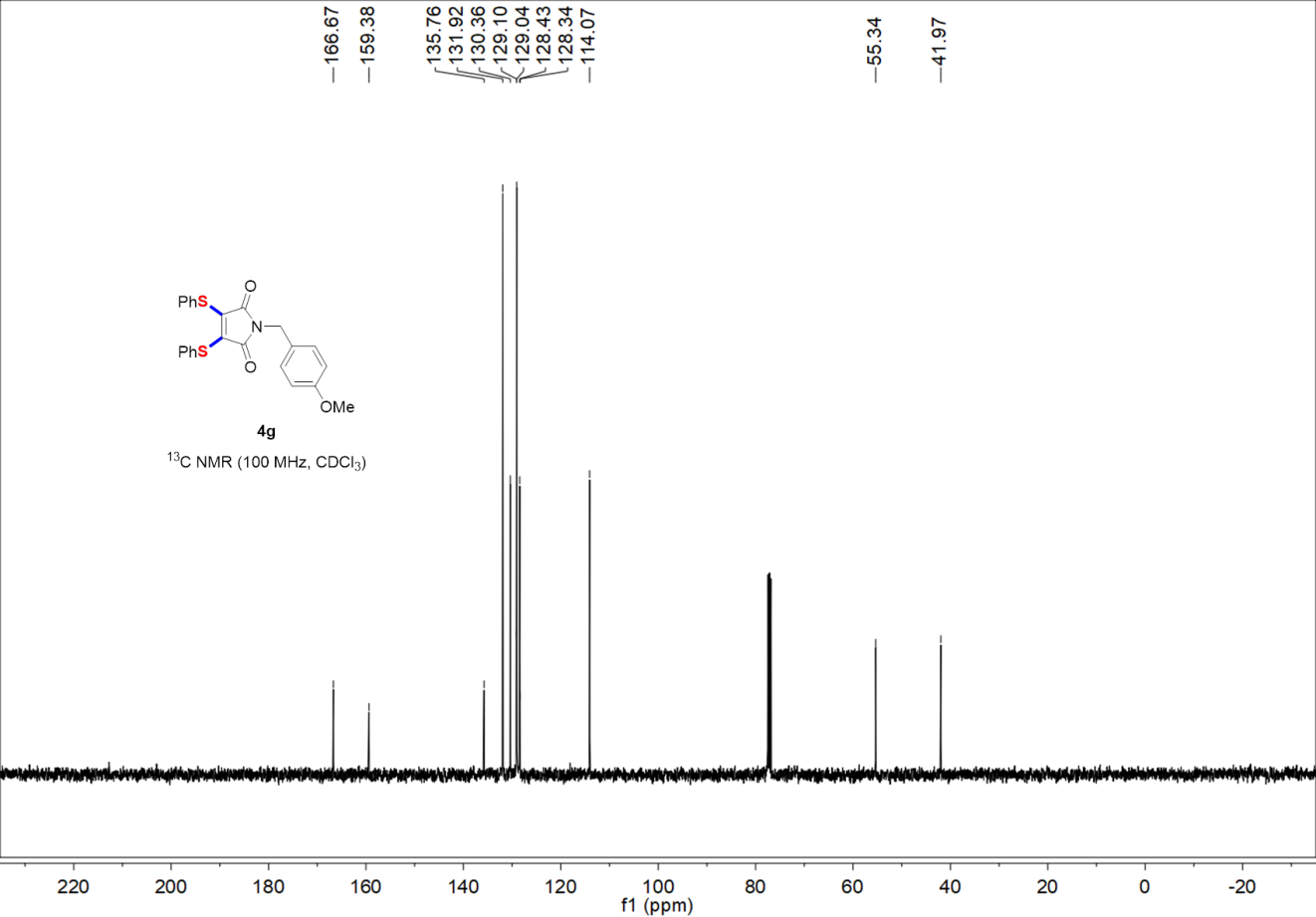
**

**
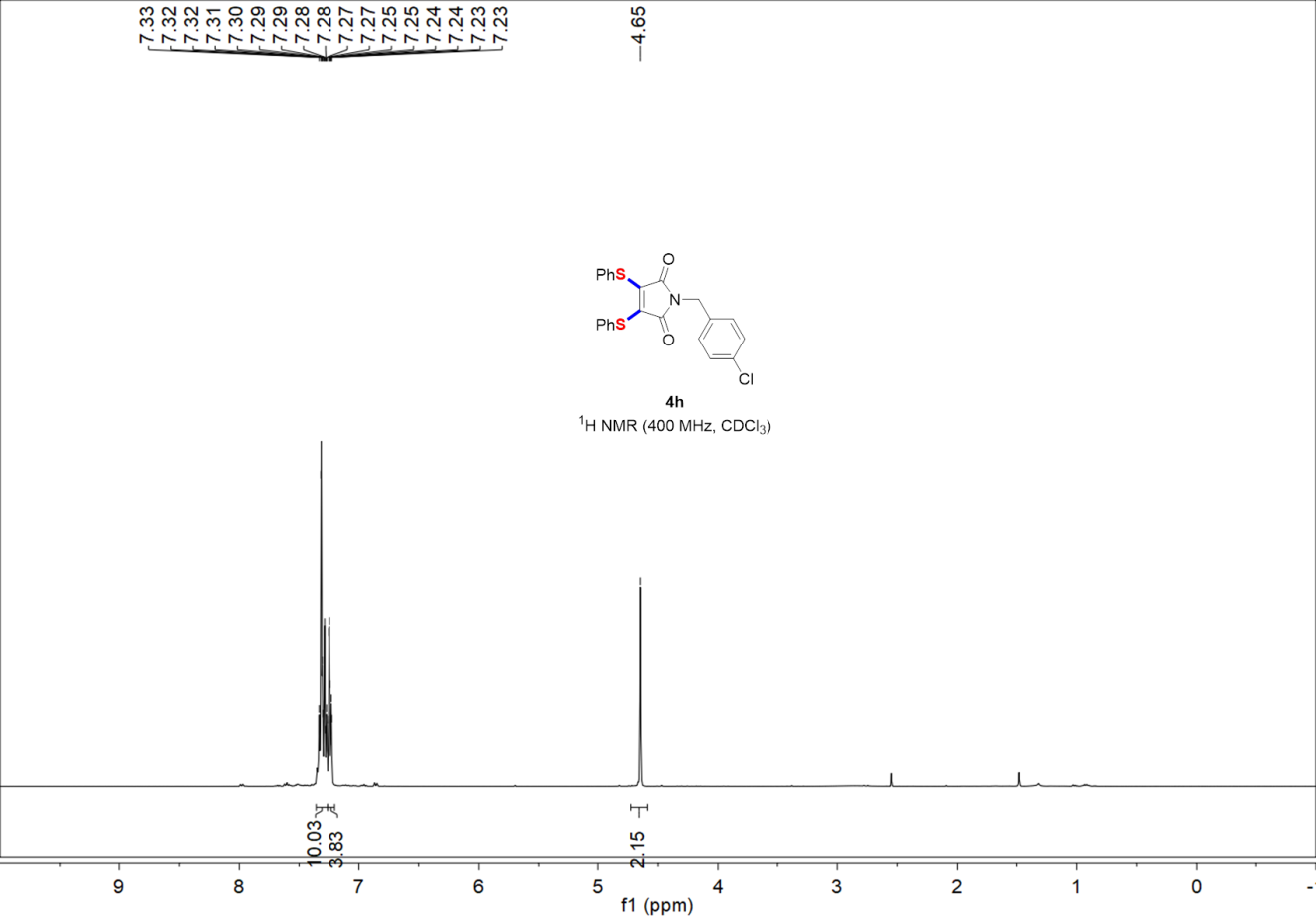
**

**
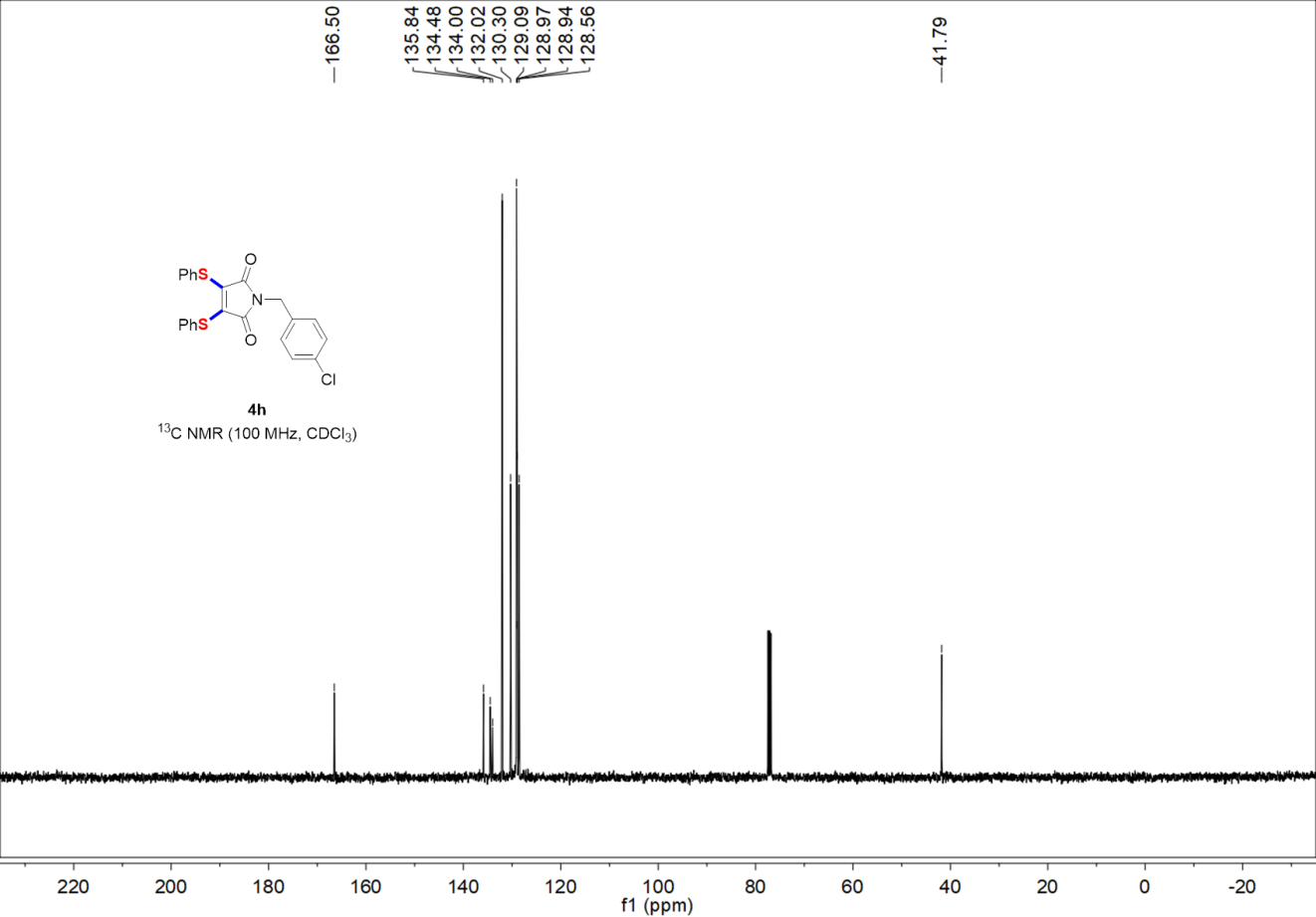
**

**
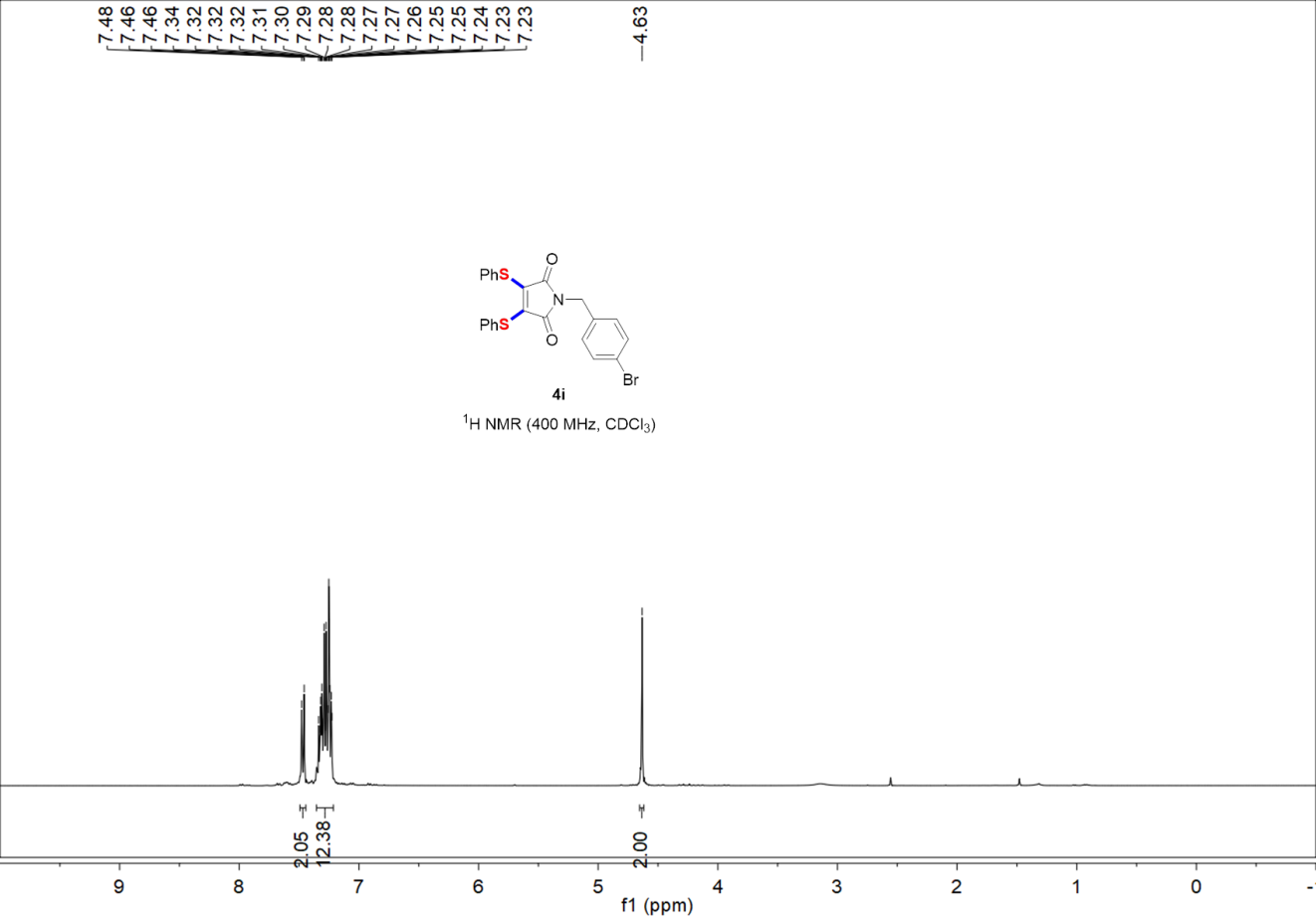
**

**
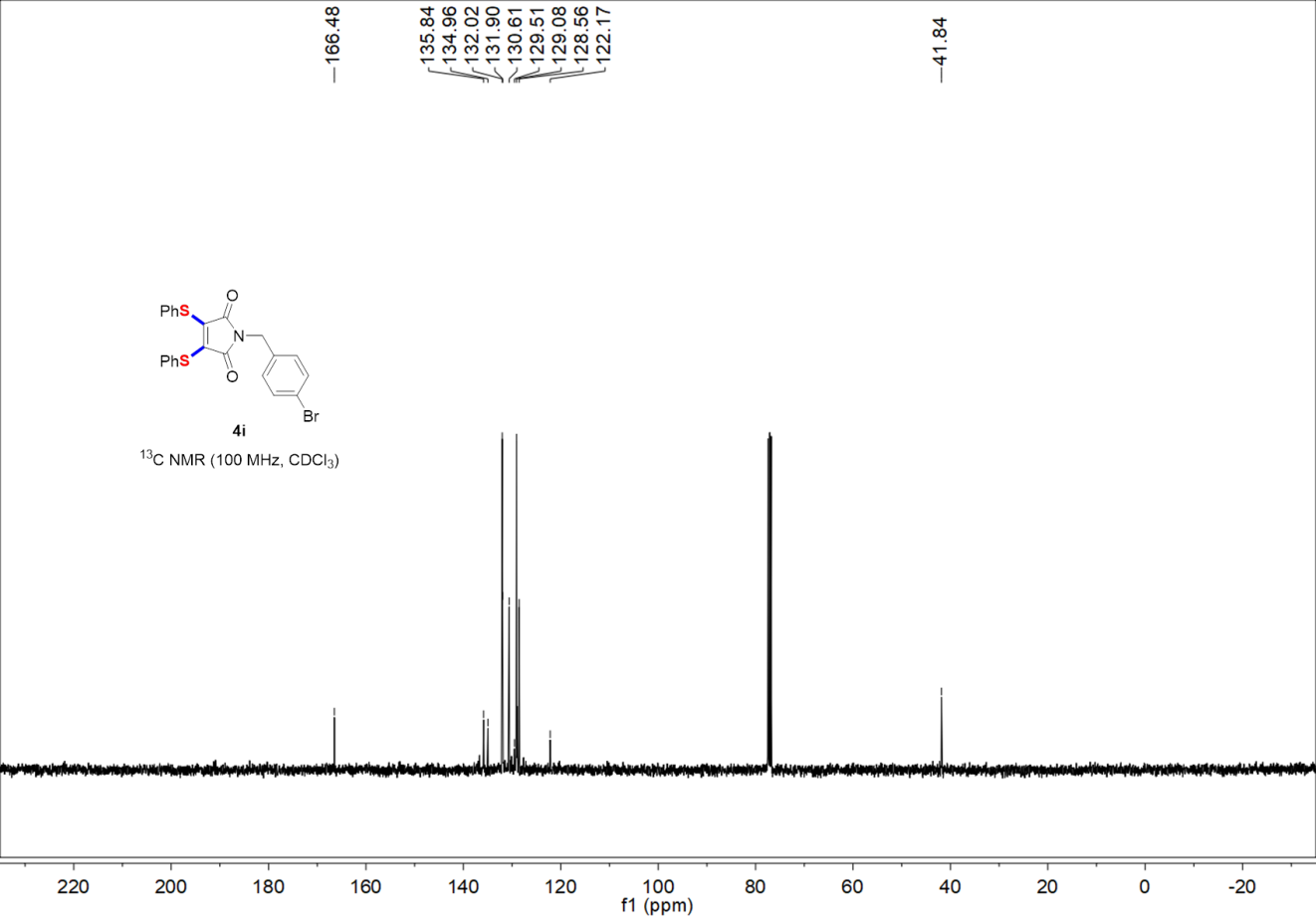
**

**
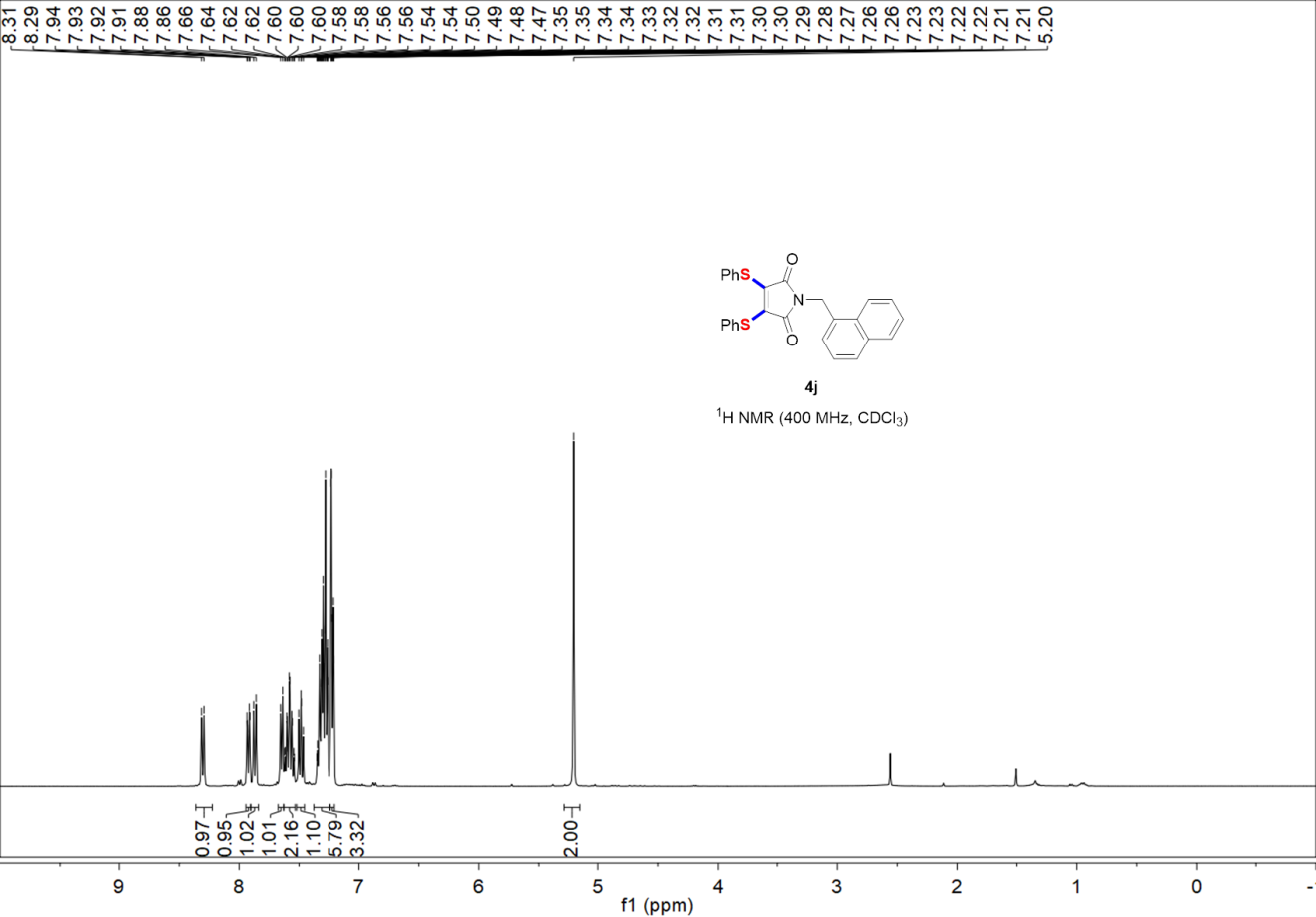
**

**
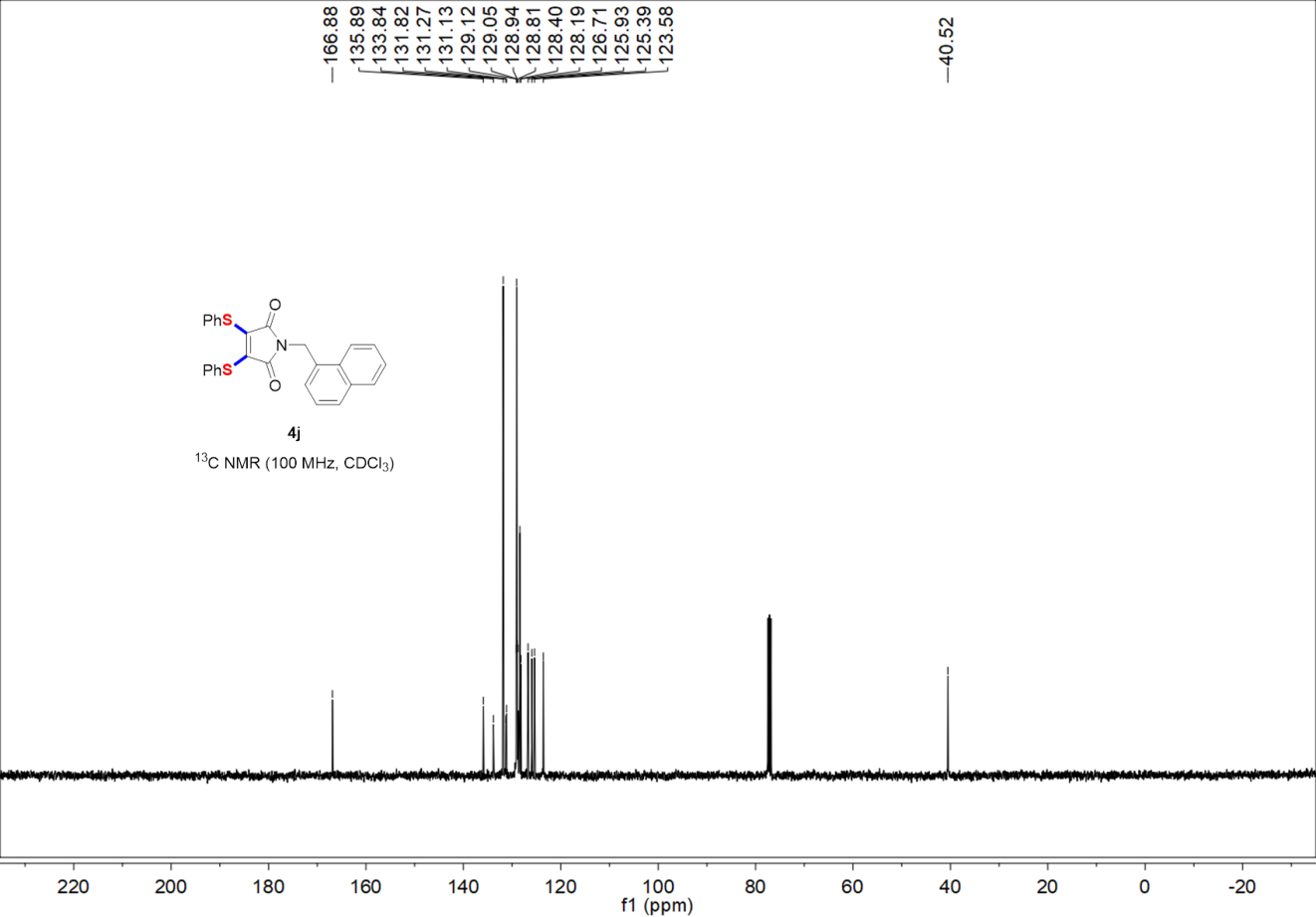
**

**
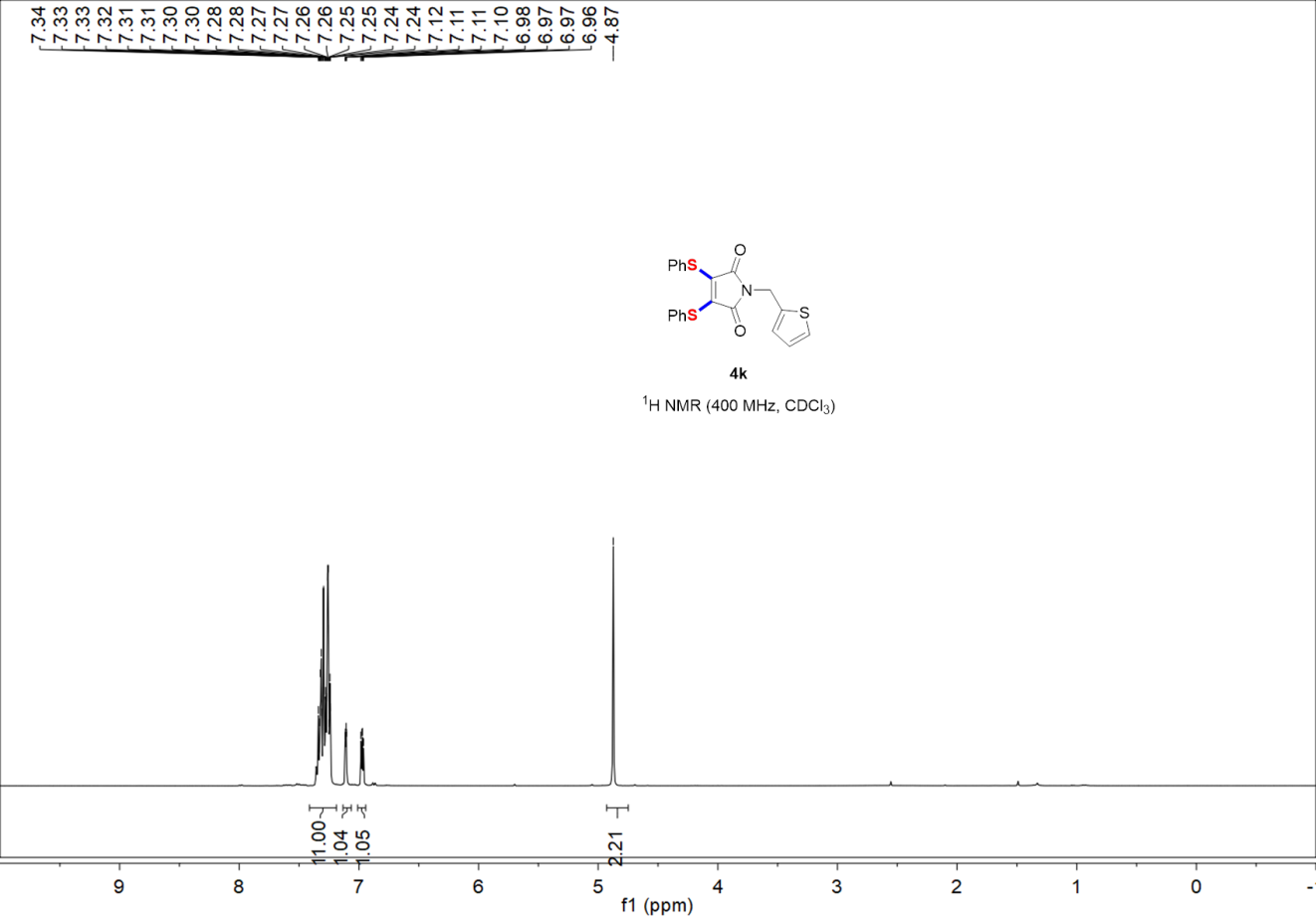
**

**
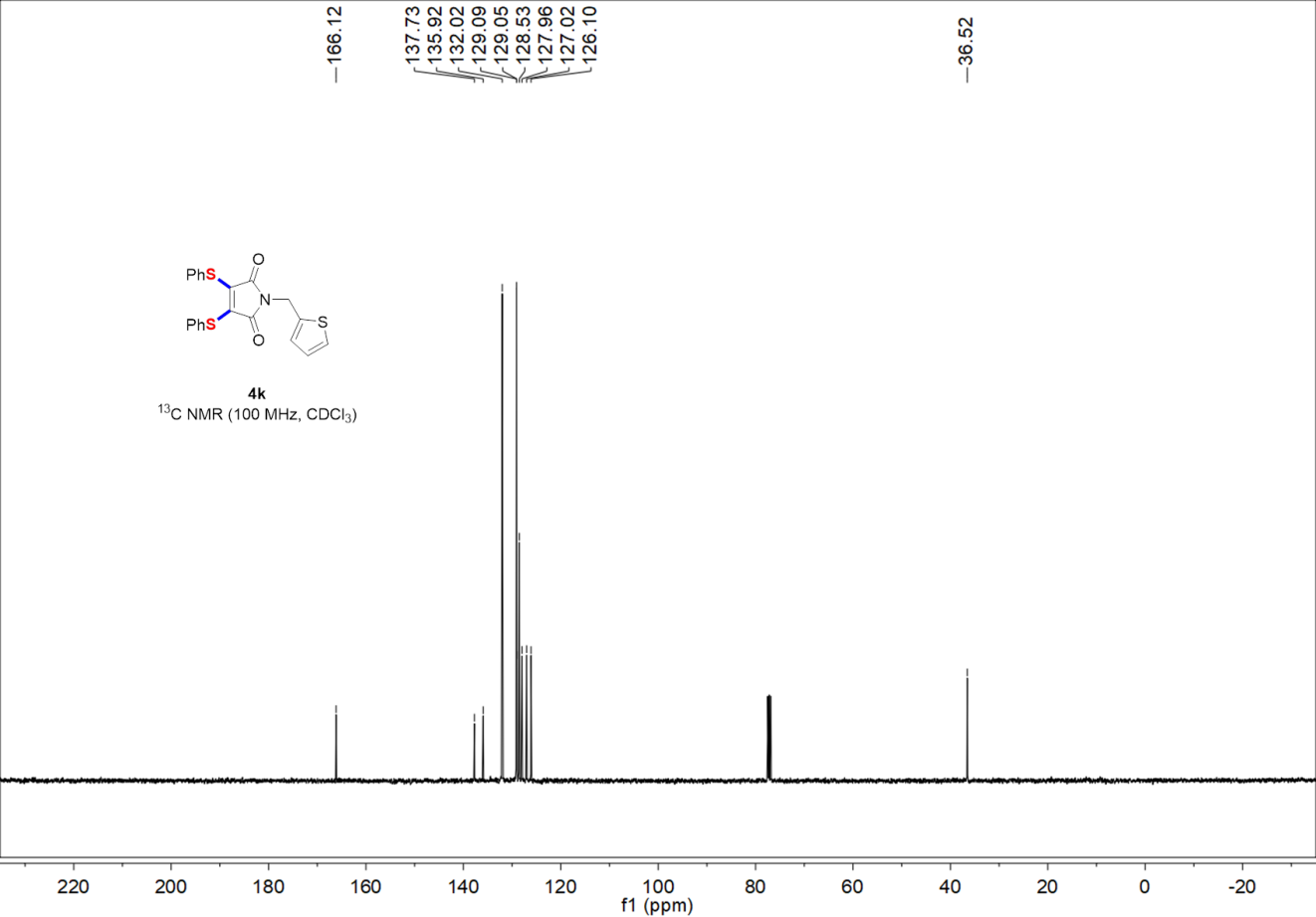
**

**
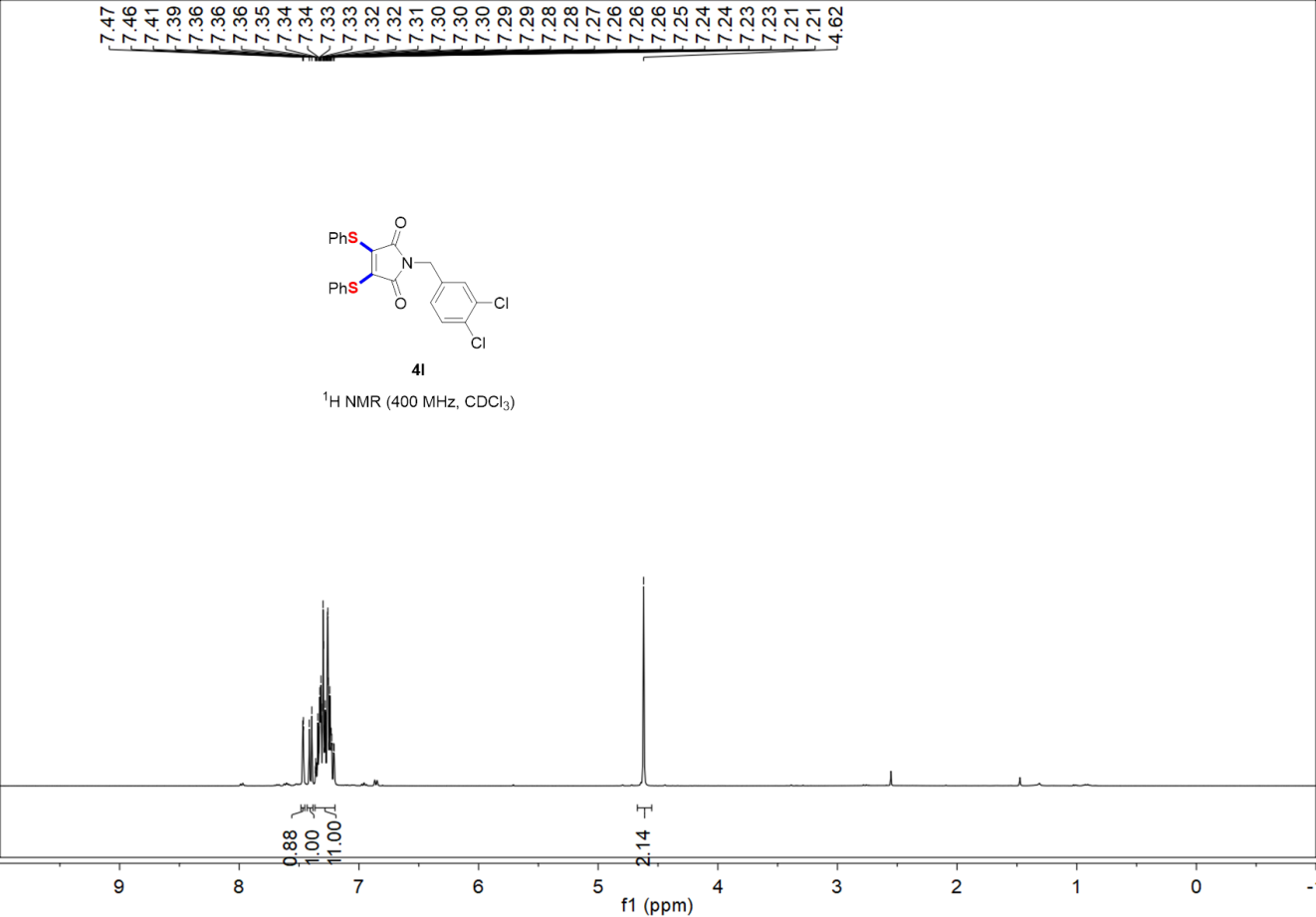
**

**
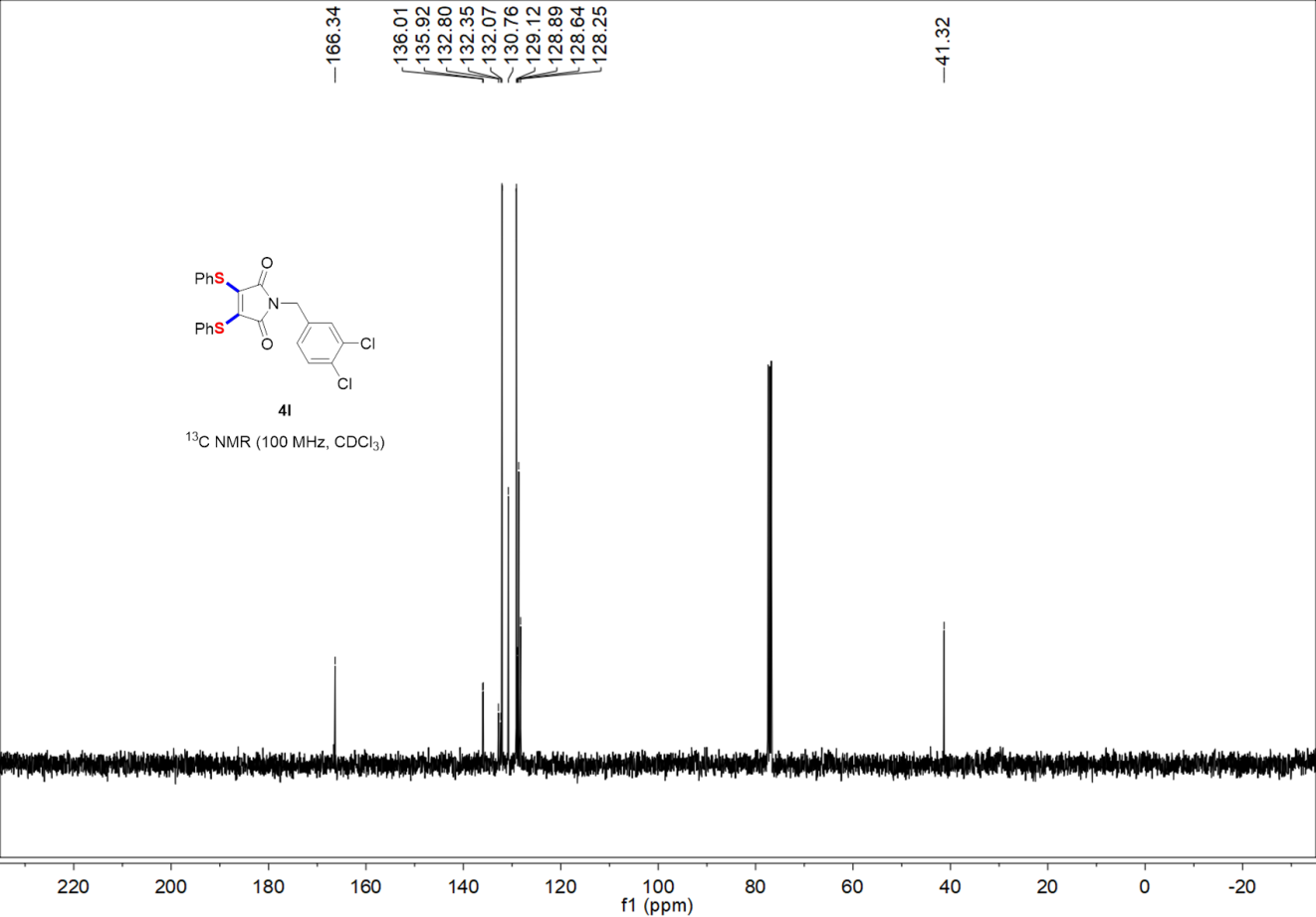
**

**
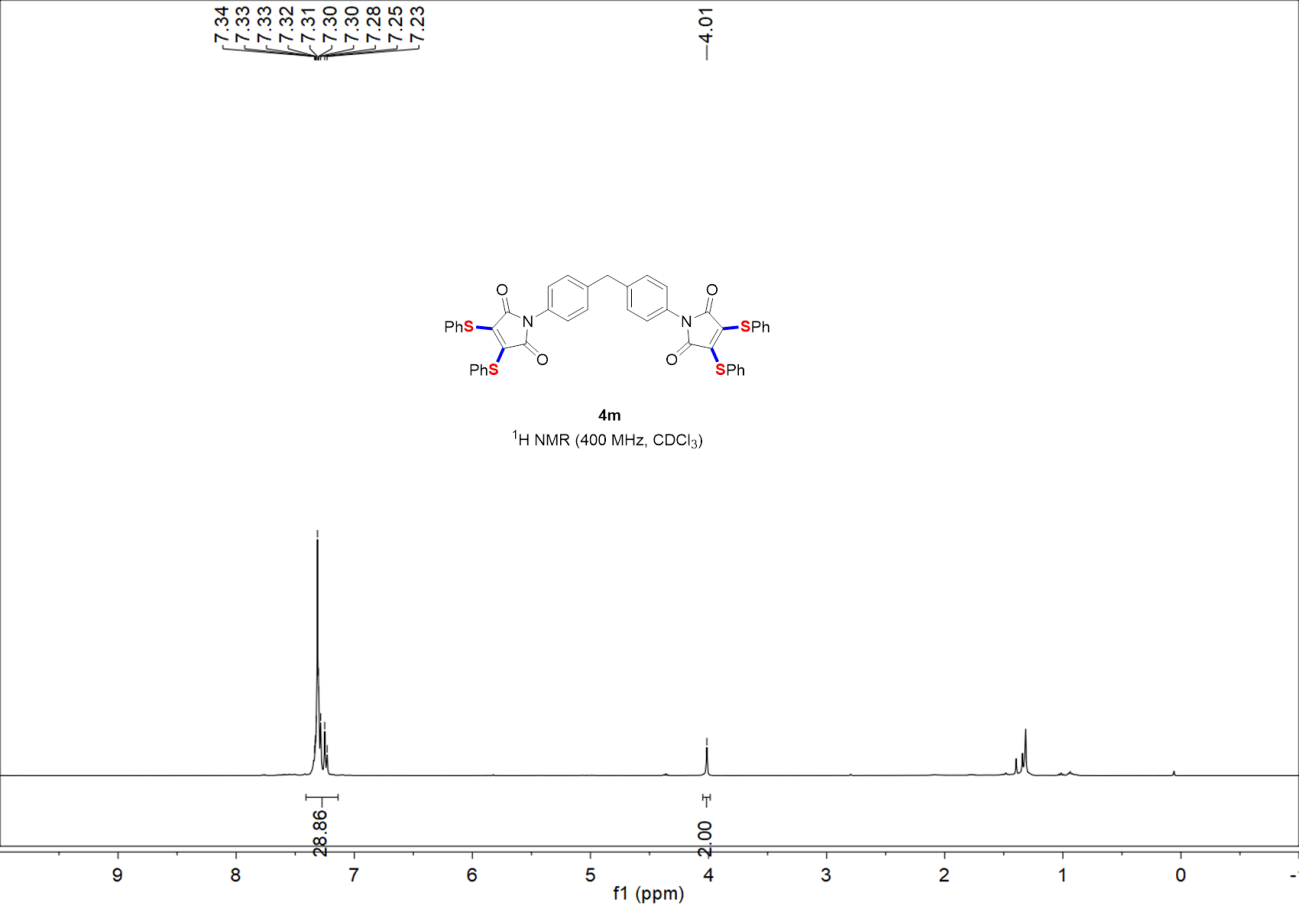
**

**
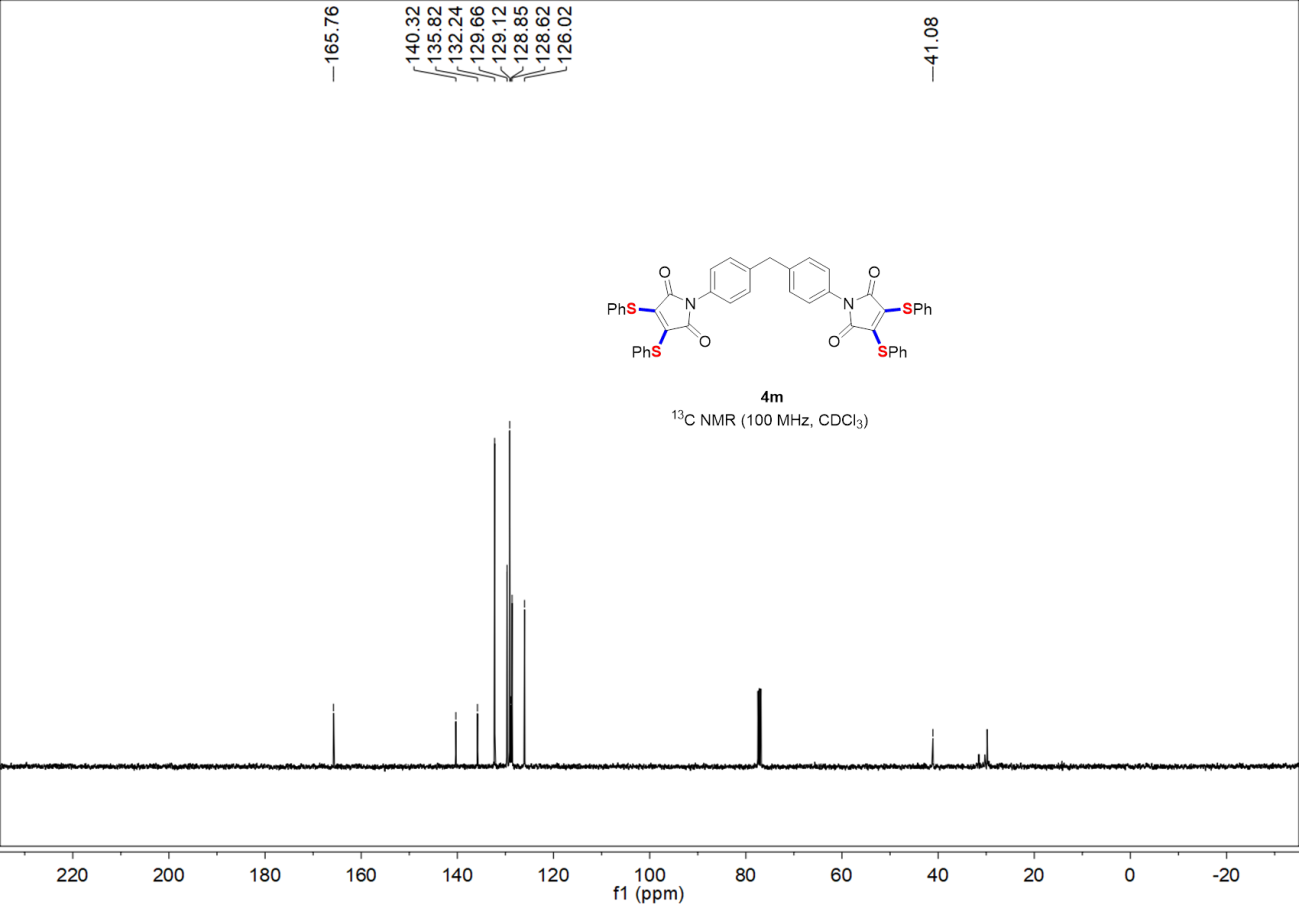
**

**
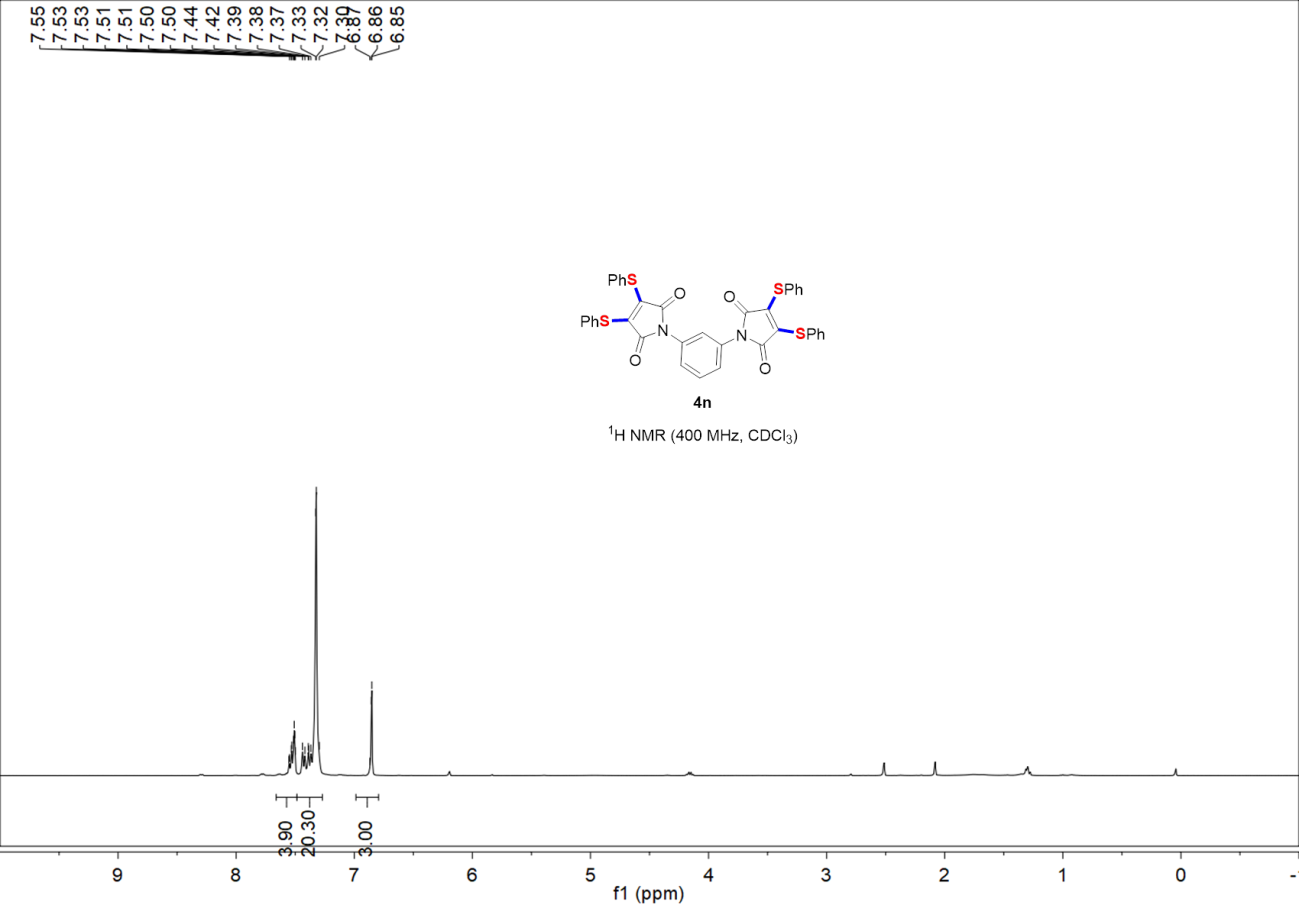
**

**
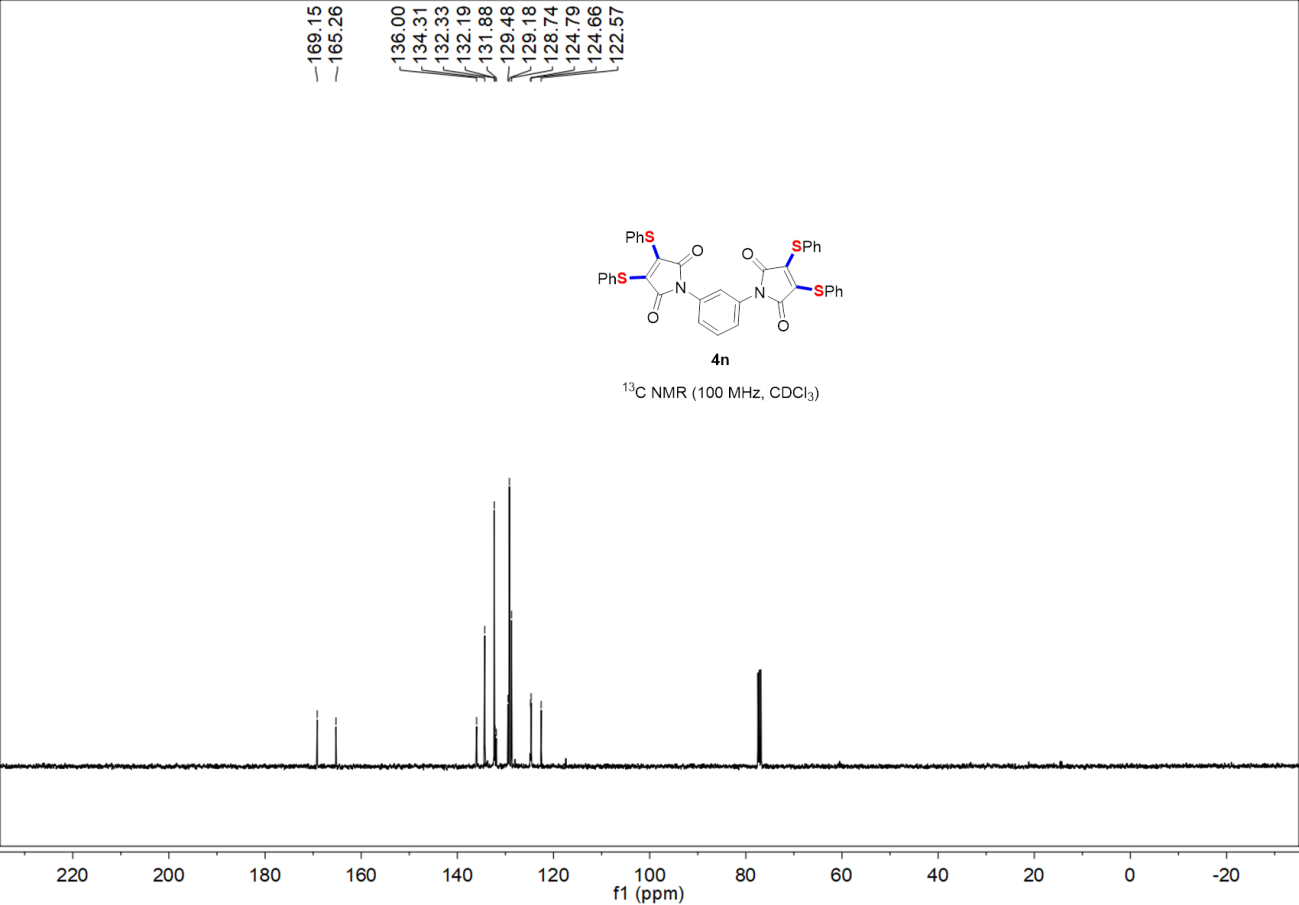
**

**
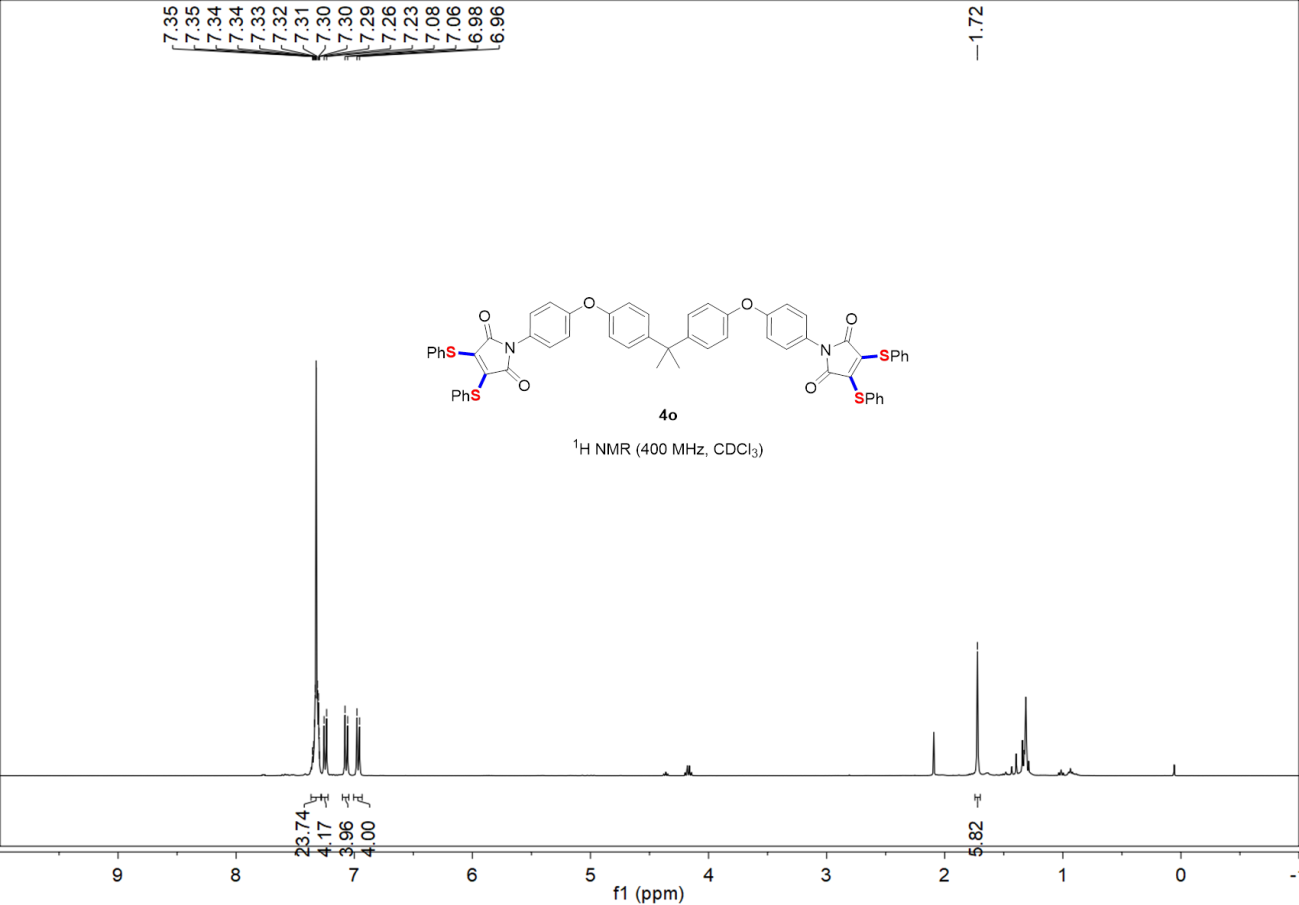
**

**
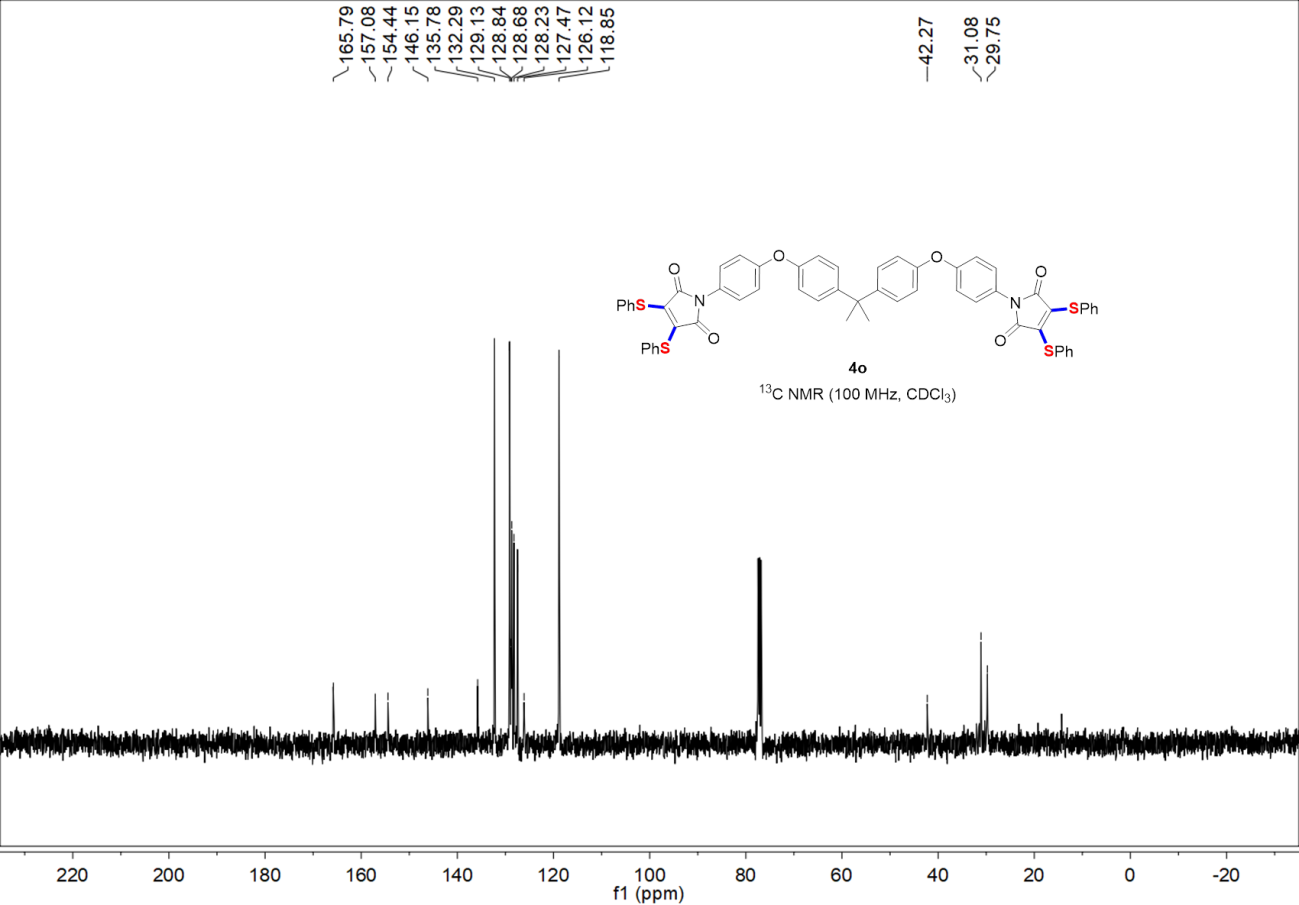
**
